# Supplementary material for: One-to-one befriending for people with intellectual disability and symptoms of depression: protocol for a pilot randomised controlled trial
Source: BMJ Open. 2020 Jun 21;10(6):e033989. doi: 10.1136/bmjopen-2019-033989 (PMC7311030; doi:10.1136/bmjopen-2019-033989)
Supplement: Author's manuscript [file bmjopen-2019-033989.draft_revisions.pdf]

BMJ Open

BMJ Open is committed to open peer review. As part of this commitment we make the peer review history of every article we publish publicly available.

When an article is published we post the peer reviewers' comments and the authors' responses online. We also post the versions of the paper that were used during peer review. These are the versions that the peer review comments apply to.

The versions of the paper that follow are the versions that were submitted during the peer review process. They are not the versions of record or the final published versions. They should not be cited or distributed as the published version of this manuscript.

BMJ Open is an open access journal and the full, final, typeset and author-corrected version of record of the manuscript is available on our site with no access controls, subscription charges or pay-per-view fees (http://bmjopen.bmj.com).

If you have any questions on BMJ Open's open peer review process please email info.bmjopen@bmj.com

One to one befriending for people with intellectual disability and symptoms of depression: A protocol for a pilot randomised controlled trial.

Journal:	*BMJ Open*
Manuscript ID	bmjopen-2019-033989
Article Type:	Protocol
Date Submitted by the Author:	31-Aug-2019

Complete List of Authors:	Ali , Afia; University College London, Division of Psychiatry<br>McKenzie, Emma; North East London NHS Foundation Trust Goodmayes Hospital<br>HASSIOTIS, ANGELA; ROYAL FREEand UNIVERSITY COLLEGE MEDICAL SCHOOL, PSYCHIATRY and BEHAVIOURAL SCIENCES<br>Priebe, Stefan; Barts and the London School of Medicine and Dentistry, University of London, Unit of Social and Community Psychiatry<br>Lloyd-Evans, Brynmor ; UCL, Division of Psychiatry<br>Omar, Rumana; University College London<br>Jones, Rebecca; UCL<br>Panca, Monica; University College London, Primary Care and Population Health<br>Fernandez, Vincent; Outward<br>Finning, Sally; Outward<br>Moore, Shirley; The Befriending Scheme<br>O'Connor, Danielle; The befriending Scheme<br>Roe, Christine; The Befriending Scheme<br>King, Michael; University College Medical School, Division of Psychiatry

Keywords:	Depression & mood disorders < PSYCHIATRY, QUALITATIVE RESEARCH, Delirium & cognitive disorders < PSYCHIATRY

SCHOLARONE™ Manuscripts

I, the Submitting Author has the right to grant and does grant on behalf of all authors of the Work (as defined in the below author licence), an exclusive licence and/or a non-exclusive licence for contributions from authors who are: i) UK Crown employees; ii) where BMJ has agreed a CC-BY licence shall apply, and/or iii) in accordance with the terms applicable for US Federal Government officers or employees acting as part of their official duties; on a worldwide, perpetual, irrevocable, royalty-free basis to BMJ Publishing Group Ltd ("BMJ") its licensees and where the relevant Journal is co-owned by BMJ to the co-owners of the Journal, to publish the Work in this journal and any other BMJ products and to exploit all rights, as set out in our licence.

The Submitting Author accepts and understands that any supply made under these terms is made by BMJ to the Submitting Author unless you are acting as an employee on behalf of your employer or a postgraduate student of an affiliated institution which is paying any applicable article publishing charge ("APC") for Open Access articles. Where the Submitting Author wishes to make the Work available on an Open Access basis (and intends to pay the relevant APC), the terms of reuse of such Open Access shall be governed by a Creative Commons licence – details of these licences and which Creative Commons licence will apply to this Work are set out in our licence referred to above.

Other than as permitted in any relevant BMJ Author's Self Archiving Policies, I confirm this Work has not been accepted for publication elsewhere, is not being considered for publication elsewhere and does not duplicate material already published. I confirm all authors consent to publication of this Work and authorise the granting of this licence.

For peer review only

One to one befriending for people with intellectual disability and symptoms of depression: A protocol for a pilot randomised controlled trial.

Afia Ali, *[1] Emma McKenzie [2], Angela Hassiotis [1], Stefan Priebe [3], Brynmor Lloyd-Evans [1], Rumana Omar [4], Rebecca Jones [1,5], Monica Panca [5], Vincent Fernandez [6], Sally Finning[6], Shirley Moore [7], Danielle O'Connor [7] Christine Roe [7], Michael King [1]

[1]Division of Psychiatry,

University College of London, UK

afia.ali@ucl.ac.uk

michael.king@ucl.ac.uk

b.lloyd-evans@ucl.ac.uk

a.hassiotis@ucl.ac.uk

reb.jones@ucl.ac.uk

[2] Research and Development Office,

Goodmayes Hospital, North East London NHS Foundation Trust, UK

Emma.mckenzie@nelft.nhs.uk

[3]Unit for Social and Community Psychiatry, (WHO Collaborating Centre for Mental Health Services Development),

Queen Mary University of London, UK

s.priebe@qmul.ac.uk.

[4] Department of Statistical Science

University College London, UK

r.omar@ucl.ac.uk

[5] PRIMENT Clinical Trials Unit,

University College London, UK

reb.jones@ucl.ac.uk

For peer review only

For peer review only - http://bmjopen.bmj.com/site/about/guidelines.xhtml

m.panca@ucl.ac.uk

[6]Outward, Newlon House

4 Daneland Walk, Hale Village, London,

N17 9FE

VFernandez@outward.org.uk

sfinning@outward.org.uk

[7] The Befriending Scheme,

1, The Croft, Sudbury,

Co10 1HN

danielle.oconnor@thebefriendingscheme.org.uk

christine.roe@thebefriendingscheme.org.uk

shirley.moore@thebefriendingscheme.org.uk

* Corresponding author

Afia Ali

Division of Psychiatry,

University College of London,

6th Floor, Maple House,

149 Tottenham court Road,

W1T 7NF

Email: afia.ali@ucl.ac.uk

Telephone: 0207 679 9334

Word count: 4919

For peer review only - http://bmjopen.bmj.com/site/about/guidelines.xhtml

Abstract

Introduction

People with intellectual disability (ID) are more likely to experience loneliness and have smaller social networks, which increases vulnerability to depression. Befriending may reduce depressive symptoms in other populations but randomised controlled trials (RCTs) have not been carried out in this population. This pilot study aims to assess the acceptability and feasibility of carrying out a full RCT of one to one befriending by volunteers for people with ID, compared to an active control group.

Methods and Analysis

The trial aims to recruit 50 participants with ID. Participants in the intervention arm will receive weekly visits from a volunteer over six months. Community befriending schemes will recruit, train, supervise volunteers and match them to individuals with ID. Both groups will receive a booklet about local activities and have access to usual care. Health and social outcomes will be measured at the end of the intervention and 6 months follow up. The following outcomes will be assessed: 1. recruitment and retention of individuals with ID and volunteers in the trial; 2. adverse events related to the intervention; 3. the acceptability of the intervention; 4. whether the intervention is delivered as intended; 5. changes in health and social outcomes and 6. the feasibility of carrying out a cost effectiveness analysis in a full trial. Qualitative data from participants, volunteers, staff and carers will identify barriers and facilitators of a future full trial.

Ethics and Dissemination

The study has been approved by the London- City and East Research Ethics Committee (reference 18/LO/2188). The findings will be presented at conferences and published in a peer reviewed journal and the National Institute of Health Research journals library. A public engagement seminar will be held at the end of the study aimed at key stakeholders.

Trial registration Number: ISRCTN63779614

Strengths and Limitations of the study

- This is the first pilot randomised controlled trial of one to one befriending in people with ID.
- This pilot study will examine the feasibility and acceptability of carrying out a future randomised controlled trial of befriending compared to an active control group.
- Community befriending services will be responsible for all aspects of the delivery of intervention
- Availability of resources within befriending services may influence the delivery of the intervention
- The findings will inform whether a full trial is feasible and what modifications would need to be made to overcome potential barriers.

For peer review only - http://bmjopen.bmj.com/site/about/guidelines.xhtml

Introduction

Intellectual disability (ID) is a life-long condition characterised by impaired cognitive and adaptive functioning, arising before the age of 18 (1). The UK prevalence of ID is 1-2% (2). People with ID have the same or higher prevalence of depression than the general population, but are more likely to experience chronic depression (3-4). They have greater exposure to social disadvantage (5), experience social exclusion because of stigma (6), have markedly smaller social networks (7-8) and a higher prevalence of loneliness compared to the general population (9). These factors have been associated with depressive symptoms in this group (10-13).

Conceptualisation of befriending

Befriending is “a relationship between two or more individuals, initiated, supported and monitored by an agency. The relationship is non-judgmental, mutual, purposeful, and there is a commitment over time” (14). Key attributes are that it is a one to one friendship-like relationship, it is an organised intervention, and that there is a negotiation of power (15). There is a wide variation in the concept and practice of befriending (16). At one extreme, befriending is very close to a friendship, characterised as being reciprocal and is delivered by lay volunteers, and at the other end it is a professional and therapeutic relationship, focused on the befriendee attaining goals and aspirations (mentoring). Most types of befriending relationships lie midway on this spectrum. Schemes are usually run by voluntary organisations in the community and offer training, supervision and on-going support to volunteers (16).

Befriending aims to help people who are lonely, isolated and have limited opportunities for social participation by increasing social and emotional support and by enhancing social networks and community participation. The causal mechanisms of befriending are unclear but social support is thought to be important. Social support may act as a buffer to stress and it may mediate genetic and environmental vulnerabilities to depression through its effects on neurobiological factors and other psychosocial factors (e.g. coping strategies) (17)The Befriender may enhance social support and link the befriendee into social activities, which may be sustainable outside of, and beyond the end of the befriending relationship, which may result in longer term benefits. Befriending may also improve health outcomes thorough its effects on social networks (18).

There are also potential benefits for befrienders. Motivation for befriending often includes a desire for “giving” something back to the community (e.g. helping others) and “getting” something in return (e.g. acquiring new skills or meeting new people) (19, 20)). Positive benefits reported by befrienders include increased self-esteem and confidence, feeling that they have gained a genuine friend themselves and improved attitudes towards people with mental illness (19). Volunteering in general has beneficial effects on depression, psychological wellbeing and life satisfaction, and is associated with lower risk of mortality, although the causal mechanisms for these associations are unclear (21).

However, the benefits of befriending may be short-lived and people with ID have reported feeling distressed following the termination of their befriending relationship (22). Other risks include the emotional turbulence that is associated with a natural friendship, or harmful

For peer review only - http://bmjopen.bmj.com/site/about/guidelines.xhtml

effects if the befriender is not adequately trained or supervised. There may also be undue burden placed on the befriender to take on excessive responsibility (16).

Effectiveness of befriending

One meta-analysis found that befriending in people with mental or physical health problems, had a statistically significant but modest effect on reducing symptoms of depression when compared to no treatment or treatment as usual in both the short and long term (23). Another systematic review examined a range of health and social outcomes in studies where befriending was delivered by volunteers only (24). Befriending was associated with better patient reported outcomes when all primary outcomes were combined but the effect size was small (24). However, in contrast to the previous review, there was limited evidence for the effectiveness of befriending on individual outcomes such as depression, loneliness or quality of life when the studies were combined (24). A recently published randomised controlled trial (RCT) of befriending by volunteers in people with psychosis provides further evidence for the potential beneficial effects of befriending (25). Participants in the intervention arm had significantly more social contacts at the end of the 12 month intervention and at six months follow up, suggesting that befriending may help to reduce social isolation in this is group.

The effectiveness of befriending in people with ID has not been evaluated in a randomised trial. An unpublished single arm feasibility study of one to one befriending by volunteers, conducted by a voluntary organisation, (26) recruited 24 volunteers, of which 15 were matched with an individual with ID. Positive change was reported in 60% of individuals with ID; 53% reported a decrease in isolation and 40% reported an increase in confidence. One Australian study examined the feasibility of using active mentoring to improve the participation of older adults with ID in mainstream community groups (27). The intervention comprised 29 individuals receiving the intervention and a matched comparison group. The participants in the intervention reported better social satisfaction compared to the comparison group but there were no significant changes in the other outcomes.

Given the dearth of studies examining befriending in people with ID there is a clear rationale for carrying out a pilot study prior to a full randomised controlled trial.

Aims and Objectives

The main aim of the study is to determine the feasibility and acceptability of a full scale randomised controlled trial of one to one befriending by volunteers for people with ID in addition to usual care, compared to an active control arm.

The objectives are to:

- examine the recruitment and retention of individuals with ID and volunteers in the trial and the number of successfully matched pairs within the six month study recruitment period
- Record any negative consequences/adverse effects of befriending
- Measure the extent to which the intervention is delivered as intended by volunteers and the befriending schemes
- Examine the acceptability of the intervention and study procedures by exploring the views of individuals, volunteers, carers and befriending services.

For peer review only - http://bmjopen.bmj.com/site/about/guidelines.xhtml

- Examine changes in health and social outcomes by carrying out exploratory analyses of the impact of befriending on depressive symptoms measured by the Glasgow Depression Scale (GDS (28)) at 12 months and other outcomes (psychological distress, self-esteem, loneliness, quality of life, and social participation) at 6 and 12 months post randomisation.
- Carry out exploratory analyses of the impact of befriending on volunteers' wellbeing, self-esteem, loneliness and attitudes towards people with intellectual disability at 6 and 12 months.
- Estimate the sample size required and determine the final trial design for a full scale randomised controlled trial.
- Assess the feasibility of collecting data that would inform a future analysis of cost effectiveness.

Methods

i. Design

This is a two arm, parallel group, researcher blind pilot RCT with 1:1 allocation. Fifty participants with ID who are eligible for the study will be randomly allocated to either the intervention arm (one to one befriending by a volunteer) or an active control arm. Both groups will have access to usual care and a booklet of local resources. The duration of the intervention will be 6 months. Outcome assessments will be carried out at baseline, six months and 12 months post randomisation. A process evaluation, based on mixed methods, will be carried out to examine the delivery and adherence to intervention, and stakeholder views on the acceptability of the intervention and barriers and facilitators that may affect the implementation of a full scale trial.

Sample Size

We have selected a sample size of 50 participants with ID based on pragmatic reasons. We do not have any estimates of the number of people with ID who are eligible and are likely to consent to taking part in the trial. A sample size of 50 will allow us to estimate an expected recruitment rate of 80% from those who are eligible, with a 95% confidence interval (CI) of 68.9% to 91.1%. A sample size of 40 recruited people with ID would allow us to estimate a 30% drop out rate in the trial with a 95% CI of 25.7% to 54.3 %. The recruitment period is 6 months. There are two participating befriending services and therefore we will need to recruit 4 participants with ID per month at each site.

Participants

Inclusion Criteria

We will include: individuals with ID aged 18 or over, who have mild or moderate intellectual disability (IQ of 35-60) assessed using the Wechsler Abbreviated Scale of Intelligence (WASI-II; (29)); should not be attending college/education or a day service for three or more days a week; will have a score of 5 or more on the Glasgow Depression Scale for People with Learning Disability (GDS-LD (28)), indicating the presence of depressive symptoms but

For peer review only - http://bmjopen.bmj.com/site/about/guidelines.xhtml

do not need to have a clinical diagnosis of depression. Participants will need to be able to speak English and provide informed consent.

Volunteers aged 18 or over, who can agree to being available once a week for at least one hour over a period of six months.

Exclusion Criteria

Individuals with ID will be excluded if they have limited communication and comprehension and therefore would not be able to complete the questionnaires.

Volunteers will be excluded if they have a criminal record (any documented offence due to the vulnerability of this population) recorded on their Disclosure and Barring Service (DBS) check or are unable to provide two references or have unsuitable references.

Recruitment

I. Participants with ID

Two befriending services (based in Hackney, North London and Suffolk) will be taking part in the study and will be responsible for overseeing the befriending intervention.

Participants with ID will be recruited from existing and new referrals to the befriending services or recruited from the caseload of clinicians working in community learning disability services in North London and Suffolk. Volunteer coordinators and clinicians will screen referrals and caseloads for participants who may be eligible for the study and will make the initial approach. If the individual is interested in the study, and consents to his/her details being passed on to the research team, a referral from will be completed and emailed to the research team. The research assistant will then contact the individual and carry out an eligibility assessment. If the individual is eligible, they will need to provide written consent before completing the baseline assessment.

II. Volunteers

The befriending services will advertise and recruit volunteers through newspaper advertisements, befriending and job websites, social media and recruitment events at colleges and universities. Volunteers will be recruited directly from the study website hosted by the UCL Division of Psychiatry and through the Division's twitter account. Interested volunteers will complete an application form and will be invited to an informal interview to assess their suitability and motivation for taking part in the scheme. A DBS check will be completed to ensure that they have no criminal records and references will be obtained. People with ID are potentially a vulnerable group and therefore volunteers with any previous offence, including minor offences, will not be included in the study. Successful candidates will be invited to take part in the study. They will receive an information sheet and asked to sign a consent form, followed by completion of the baseline assessment.

Randomisation

Randomisation will be carried out after the baseline assessments have taken place. Participants will be randomised into the study by an unblinded member of the research team who will enter the patients' details into a web based randomisation system, which is hosted on a secure server by Sealed Envelope. This system will randomly allocate the participant to either the intervention

For peer review only - http://bmjopen.bmj.com/site/about/guidelines.xhtml

or control arm. The unblinded researcher will notify the befriending service and participants with ID of their allocation.

Randomisation will be blocked using randomly varying block sizes, stratified by centre. The allocation schedule will be concealed through the use of this central web-based randomisation service. The randomisation protocol will be created by the trial statistician and the set up of the service will be overseen by the Priment Clinical Trials Unit.

Intervention group

Participants with ID will meet with the volunteering coordinator to obtain information about their hobbies, interests and activities they would like support to participate in. Based on this information, participants with ID will be matched to a volunteer that can accommodate the person's interests.

i. The befriending intervention

The befriending intervention has been adapted from the existing models used by the two participating befriending services and from other studies of befriending (26, 30). The purpose of the befriending relationship will be to provide friendship and emotional support, and to assist the individual to access activities in the community that they may be unable to do alone. As participants become more confident, they will be encouraged to access activities in the community on their own, in order to promote sustained social activities beyond the befriending relationship.

The volunteer (befriender) and person with ID will meet once a week for one hour, for six months, although some breaks are anticipated due to holidays or illness. They will receive a booklet detailing local activities and amenities, which they can use to plan activities. The emphasis will be on assisting the individual to make choices about the activities that they wish to pursue. The volunteer is not expected to carry out personal care, administer medication or accompany the individual to medical appointments. Contacts by phone/social media can take place in addition to face to face contacts. The pair can spend some sessions in the person's home but this should not exceed 50% of the total number of sessions. Sessions may take place during evenings/weekends depending on the pair's availability. They will keep a record of their activities in a structured log that will be provided (whether they attended each session, reasons for cancellation, what they did in each session and duration of activity), and record of other types of contact. Volunteers will be reimbursed travel expenses but other expenses will need to be agreed with the befriending service. Participants with ID will not be reimbursed travel expenses or the costs of activities.

ii. Introduction and monitoring of the befriending relationship

The volunteering coordinator will arrange a face-to-face meeting where the pair will be introduced to each other. If they agree to continue, the pair will arrange to meet on their own; if they decide that the pairing is unsuitable, they will be re-matched. If the volunteer or individual with ID drops out of the relationship once it has become established, attempts will be made to re-match them. Volunteers may be matched to more than one participant with ID.

For peer review only - http://bmjopen.bmj.com/site/about/guidelines.xhtml

The volunteering coordinator will arrange a face-to-face meeting after six weeks and four weeks thereafter to monitor the progress of the relationship. A further meeting will be held at the end of the six months to obtain general feedback about the befriending intervention, to discuss ending the relationship and to support the individual with ID with coming to terms with the ending. The pair may continue their relationship if they wish after the six month period but arrangements for monitoring the relationship will vary depending on the befriending service. Information will be obtained on any relationships continuing beyond six months and the monitoring that has been provided.

iii. Training and supervision of volunteers

The volunteers will attend training delivered face-to-face or as e-learning. The training will cover: the benefits of befriending and issues related to confidentiality and lone working; advice on how to plan meetings effectively; health and safety; safeguarding ; learning disability awareness, and professional boundaries, which covers dealing with sensitive issues, ending relationships and expectations of the role of the volunteer. Volunteers will also receive slides and a manual developed by the research team, with information about the study and core aspects of the training.

Volunteers will have access to group or individual supervision delivered face to face or over the phone, once a month, by the volunteer coordinator, which will address issues that may have arisen from the relationship, for example, concerns about mental health or behaviour.

Control Group

Participants in the control arm will receive the activities booklet. They will meet with a member of the research team who will discuss the booklet with them (and their carer, if present) and encourage them to engage with activities. This is to control for the effects of participants in the treatment arm receiving more information about local activities.

Both the control and intervention arms will also have access to “usual care”. This will include access to multidisciplinary input from community intellectual disability services. Participants can continue to take their usual medication and can access other community and hospital health services and day services. Information about usual care will be collected as part of the baseline assessment.

Outcomes

i. Recruitment and retention of participants:

We will examine the proportion of participants with ID recruited from among those eligible; and the proportion of volunteers recruited from among people expressing an interest over a six months period; the proportion of participants with ID who are successfully matched with a volunteer; the proportion of participants with ID and volunteers who drop out of the intervention arm and the proportion of participants and volunteers who complete subsequent follow up assessments.

ii. Adverse events

For peer review only - http://bmjopen.bmj.com/site/about/guidelines.xhtml

Adverse events will be collected at each follow up assessment using open-ended questions and will also be reported directly to the Chief Investigator by the befriending services, including concerns about safeguarding. All adverse events will be recorded in the medical records and CRFs. Serious adverse events (e.g. safeguarding concerns, hospitalisation) will be recorded in the Serious Adverse Effects (SAE) log.

iii. Acceptability of the intervention

This will be informed by data on retention/ drop out of volunteers and participants, the extent of engagement with the intervention by participants and volunteers (based on number of sessions attended) and qualitative data obtained from volunteers, participants with ID, carers of people with ID and staff from the befriending service.

iv. Adherence to the intervention

Data will be collected on volunteer training, uptake of supervision and the frequency of monitoring checks carried out by volunteer coordinators from routine records at each site in order to assess fidelity to the intervention by the befriending services. Structured logs provided by volunteers will be analysed to assess fidelity to the intervention by volunteers. We will examine: a) how many sessions were attended by each volunteer, reasons for non-attendance, and how many attended at least 10 sessions during the 6-month intervention period; and b) for how many participants the minimum threshold of at least 50% of meetings being outside the participant's home was achieved.

v. Depression and other health and social outcome measures in participants with ID

All the health and social outcome measures have been validated in people with ID and will be assessed with the participant at baseline, at the end of the intervention and at six months follow up. (see table 1: schedule of assessments). Assessments will be carried out by a research assistant blind to group allocation and will take place face to face at the befriending service or the participants' homes.

- Depressive symptoms will be measure using the GDS-LD (44). Scores at 12 months will be the main health outcome of interest.
- Self-esteem will be measured using the adapted Rosenberg self-esteem scale for people with intellectual disabilities (31)
- Quality of life will be measured using the Maslow Assessment of Needs Scale-Learning Disability (MANS-LD; (32)) and five items from the adapted World Health Organisation Quality of Life questionnaire (WHO-QOL-8; (33)).
- Loneliness and social satisfaction will be measured using the Modified Worker Loneliness Questionnaire (MWLQ (34),
- Social support will be measured using the Social Support Self Report for intellectually disabled adults (SSSR) (35)
- Social participation will be measured at using the Guernsey Community Participation and Leisure Assessment (GCPLA) (36).

vi. Volunteer outcome measures

For peer review only - http://bmjopen.bmj.com/site/about/guidelines.xhtml

The following outcome assessments will be carried out in volunteers prior to matching and after six months (see table 2 schedule of assessments):

- Self-esteem will be measured using the 10 item Rosenberg self-esteem scale (37)
- Psychological wellbeing and quality of life will be measured using the Warwick-Edinburgh mental wellbeing scale (WEMWBS; 38).
- Loneliness will be measured using the UCLA loneliness scale (39).
- Attitudes of volunteers will be assessed using the 67 item, Attitudes Towards Intellectual Disability Questionnaire (ATTID; 40)

vii. Feasibility of carrying out a cost effectiveness analysis

The preliminary health economic analysis will inform planning of future economic analyses, sources of data required and how best to collect these data. The following measures will be recorded at baseline, six months and 12 months post randomisation.

- Health related quality of life will be measured using the EuroQol-Youth (EQ-5D-Y) (41). We will assess the feasibility of using the EQ-5D-Y to calculate Quality adjusted life years (QALYs). QALYs will be calculated using the EQ-5D-3 L tariff, as there are no value sets for the EQ-5 D-Y (Ref).
- Client Services Receipt Inventory (CSRI) for people with intellectual disabilities adapted for the study (42), completed with carers where possible, will assess the feasibility of collecting participant reported service use over the previous six months.

Process evaluation

A process evaluation will be undertaken, based on MRC guidance (43). The aim will be to examine whether the different components of the intervention were consistently followed; the extent to which volunteers delivered the intervention as intended; to understand the perceived value, benefits and unintended consequences of the intervention so that these are fully measured in a full trial, contextual factors influencing how the intervention was delivered; and to develop an understanding of the likely mechanisms of action of the intervention. We will interview 16 participants with ID after their 12 month follow up (8 per site including) and carry out two focus groups at each site with 5-8 volunteers, staff from each befriending service, and 5-8 carers at each site. We will use purposive sampling in order to include people with a range of demographic characteristics (e.g. gender, age, ethnicity, living arrangements), and both participants and volunteers for whom the befriending relationship broke down, as well as people who completed the intervention. All respondents will be asked about what aspects of the intervention were perceived to be helpful, what aspects require modification and suggestions for improvement, views about trial procedures and the perceived barriers and facilitators to delivering a larger trial. The interviews and focus groups will be audio-taped and transcribed.

In order to understand how the befriending intervention was delivered, the location, content of the meetings and the range of activities undertaken will be described based on an analysis of structured log books. A framework will be developed to categorise different types of activity to enable different types of befriending support to be distinguished and quantified. Test procedures will be developed for collecting quantitative process data, which, in a future

For peer review only - http://bmjopen.bmj.com/site/about/guidelines.xhtml

larger trial, could be used to explore the relationships between process variables and outcomes. Findings from the process evaluation will inform any necessary refinements to the study intervention or procedures.

Analysis

Feasibility outcomes such as the number of participants who were screened and eligible, the proportion of eligible participants with ID and interested volunteers recruited to the study, and the proportion successfully matched in befriending relationships, will be reported. The number (proportion) of drop-outs of volunteers and participants from the intervention arm and from both arms at each follow up assessment, will also be reported, including reasons why where possible. This information will be presented in a Consort diagram describing the flow of participants through the study. (http://www.consort-statement.org/).

The characteristics and outcomes of participants by trial arm will be summarised using means and standard deviations or medians and interquartile ranges for continuous variables and count and percentages for categorical variables. Appropriate regression models depending on the type of outcome, adjusted for baseline values and centre will be used to estimate the intervention effect on health and social outcomes where possible. The results will be presented as estimates with 95% CI. All analyses will be carried out as randomised with available data (intention to treat principle) and will be used to inform the definitive trial. The characteristics of patients with missing outcome data will be investigated.

Economic Evaluation

We will assess the feasibility of gathering information for a cost-effectiveness analysis for a full RCT including testing the suitability of calculating quality-adjusted life years (QALYs) using the EQ-5D-Y. We will calculate the costs of delivering the befriending intervention (training, supervision and expenses), which will be obtained from each participating befriending service. Mean resource use/ costs (standard deviation (SD)) per participant at baseline and follow-up, based on completion of the CSRI, will be presented.

Qualitative analysis

Transcripts from the focus groups and interviews will be analysed by the study team using thematic analysis supported by computer software (NVivo 9). The analytic strategy will focus on identifying barriers and facilitators to implementing the intervention successfully, its benefits to participants and mechanisms of effect. Analysis will also allow consideration of themes that arise more inductively from the data. Validity will be enhanced by a collaborative analytic strategy involving members of the research team and the advisory group who will meet together to review the coding framework and agree on the themes.

Patient and Public Involvement

There will be advisory groups comprising carers, current volunteers and individuals with ID who will provide advice about the study information sheets, consent forms, topic guides for the qualitative interviews and focus groups, results of the study findings and the final study report. Members will be invited to be part of the Trial Management Group (TMG) and will be involved in carrying out the qualitative interviews and focus groups as part of the process

For peer review only - http://bmjopen.bmj.com/site/about/guidelines.xhtml

evaluation. They will receive training and support for this role. We will also invite members to participate in the public engagement seminar at the end of the study.

Ethics and Dissemination

Amendments to the study protocol and documents will be approved by the sponsor (UCL) and the Ethics Committee. Priment Clinical Trials Unit will ensure that the trial procedures meet the requirements of Good Clinical Practice (GCP) and will complete data quality assurance checks. An independent trial steering committee will provide overall supervision of the trial and will report to the funders. Data confidentiality will be maintained by assigning participants study identification numbers and data will be entered anonymously and stored in a secure web-based database.

The findings of the study will be presented at conferences and published in an open access peer reviewed journal as well as the National Institute of Health Research (NIHR) Public Health Research Journal. A public engagement seminar targeted at relevant stakeholders will take place at the end of the study to discuss the study findings and implications. A summary report will be developed for participants and volunteers taking part in the study and will be published on the study website.

Study timeline

Recruitment of participants began in April 2019. There will be a six month recruitment period, a further 12 months to complete the follow up assessment and three months to complete the process evaluation, analyse the results and write up the study findings.

Discussion

This is the first pilot RCT of one to one befriending, monitored by community befriending services, in people with ID. The trial will provide data on whether a full trial will be feasible, in terms of recruitment and retention of volunteers and people with ID and data on potential beneficial and adverse effects, acceptability and the extent to which the intervention is delivered as intended by volunteers and the befriending services. It will help to inform modifications that need to be made to enable a future trial to overcome barriers and challenges that may be encountered in this pilot.

There is currently limited evidence supporting the choice of a primary health outcome but the use of depressive symptoms is supported by one systematic review (24). This pilot study will help to determine whether measuring depressive symptoms is the most appropriate primary outcome for a future trial.

One of the main challenges of this study will be to ensure that the intervention and the recruitment, training and supervision of volunteers are carried out according to the trial protocol as these aspects vary between befriending services, and was an issue in a recent trial of befriending in people with psychosis (25) While both befriending services have committed to the study, there are resource implications for these services as the intervention costs are not covered by the research funding. External factors such as funding cuts to these services could influence the delivery of the intervention and the success of the trial. The

For peer review only - http://bmjopen.bmj.com/site/about/guidelines.xhtml

process data will allow us to examine whether a future trial would need to be more pragmatic and whether the befriending intervention should permit more flexibility.

Author Contributions: AA is the chief investigator and is responsible for the overall study design and conduct. AA, SP, MK, AH, BL-E and RO have contributed to the design of the study and methodology. SF, VF, DO, CR, SM are responsible for designing and delivering the intervention. EM will carry out the data collection. RO, RJ and MP have contributed to the analysis plan. All the authors have reviewed and approved the manuscript.

Funding statement: The study/ project is funded by the National Institute of Health Research (NIHR) (Public Health Research Funding Stream (project reference: 16/122/57). The views expressed are the views of the authors and are not necessarily those of the NIHR of the Department of Health and Social Care.

Competing Interests statement: There are no conflicting interests to declare.

For peer review only

Table 1: Schedule of assessments in participants with ID

Assessment/Outcome	Screening and baseline assessment	End of intervention assessment	6 month follow up
Eligibility confirmation:<br>WASI-II<br>GDS-LD	x		
Informed consent	x		
Socio-demographic questionnaire	x		
Adapted Rosenberg self-esteem scale	x	x	x
MANS-LD	x	x	x
WHO-QOL-8	x	x	x
MWLQ	x	x	x
SSSR	x	x	x
GCPLA	x	x	x
EQ-5D-Y	x	x	x
CSRI	x	x	x
Adverse effects review	x	x	x
Concomitant medication	x	x	x
Semi-structured interview (optional)			x

WASI-II (Wechsler Abbreviated Scale of Intelligence, 2nd edition); GDS-LD (Glasgow Depression Scale for People with Learning Disability); MANS-LD (Maslow Assessment of Needs Scale learning Disability); WHO-QOL-8 (World Health Organisation Quality of Life questionnaire); MWLQ (Modified Worker Loneliness Questionnaire; SSSR (Social Support Self Report for intellectually disabled adults); GCPLA (Guernsey Community Participation and Leisure Assessment); EQ-5D-Y (EuroQol-Youth); CSRI (Client Services Receipt Interview).

Table 2: Schedule of assessments with volunteers

Assessment/ outcome	Screening and baseline	End of intervention assessment	6 month follow up
Eligibility check:<br>Informal interview;<br>DBS check<br>references	x		
Informed consent	x		
Rosenberg self-esteem scale	x	x	x
WEMWBS	x	x	x
UCLA Loneliness Scale	x	x	x
ATTID	x	x	x
Focus group (optional)			x

WEMWBS Warwick- Edinburgh mental wellbeing scale; ATTID (Attitudes Towards Intellectual Disability Questionnaire)

For peer review only - http://bmjopen.bmj.com/site/about/guidelines.xhtml

References

1. *American Psychiatric Association*. *Diagnostic and Statistical Manual of Mental Disorders (5th ed.).* Arlington, VA: American psychiatric Publishing; 2013.

2. Emerson E, Hatton C, Robertson J, Roberts H, Baines S, Glover Gyles. *People with learning disabilities in England 2010: services and supports.* Improving Health and Lives Learning Disability Observatory, 2010 (accessed from. http://www.improvinghealthandlives.org.uk/uploads/doc/vid_9244_IHAL2011-02PWLD2010.pdf

3. Cooper S-A, Smiley E, Morrison J, Williamson A, Allan L. An epidemiological investigation of affective disorders with a population-based cohort of 1,023 adults with intellectual disabilities. *Psychological Medicine*, 2007;37:873–82.

4. Richards M, Maughan B, Hardy R, et al. Long-term affective disorder in people with mild learning disability. *British Journal of Psychiatry*, 2001;179:523-7.

5. Emerson E and Einfeld SL. *Challenging Behaviour*. Cambridge: Cambridge University Press; 2011

6. Ali A, Hassiots A, Strydom A, King M. Self stigma in people with intellectual disabilities and courtesy stigma in family carers: A systematic review. *Research in Developmental Disabilities*, 2012; 33: 2122-2140

7. Verdonschot M.M.L,deWitte L.P, Reichrath E, Buntinx W.H.E, &Curfs L.N.G. Impact of environmental factors on community participation of persons with an intellectual disability: A systematic review. *Journal of Intellectual Disability Research*, 2009; 53(1), 54–64.

8. Hill RA & Dunbar RIM. Social network size in humans. *Human Nature*, 2003; 14(1):53-72.

9. Petroutsou A, Ali A, Hassiotis A. Loneliness in people with intellectual and developmental disorders (IDD) across the life span: a systematic review of prevalence and interventions. *Journal of Applied Research in Intellectual Disability* (published online Feb 2018).

10. Ali A, King M, Strydom S, Hassiotis A. Self-reported stigma and symptoms of anxiety and depression in people with intellectual disabilities: Findings from a cross sectional study in England. *Journal of Affective Disorders*, 2015, 187:224-231

11. Emerson E. Self reported exposure to disablism is associated with poor self reported health and wellbeing among adults with intellectual disabilities in England: a cross sectional survey. *Public Health*, 2010; 124, 682-689

For peer review only - http://bmjopen.bmj.com/site/about/guidelines.xhtml

12. Ailey S H, Miller A M, Heller T, & Smith E V. Evaluating an interpersonal model of depression among adults with Down syndrome. *Research and Theory for Nursing Practice*, 2006; *20*(3), 229-246.

13. Merrick, J., Merrick, E., Lunsky, Y., & Kandel, I. A review of suicidality in persons with intellectual disability. *The Israel Journal of Psychiatry and Related Sciences*, 2006; *43*(4), 258-264.

14. Dean, J. & Goodlad, R.*Supporting Community Participation: the role and impact of befriending.* Brighton: Pavilion Publishing & Joseph Rowntree Foundation, 1998.

15. Balaam MC. A concept analysis of befriending. J Adv Nurs, 2015, 71(1): 24-34.

16. Thompson R, Valenti E, Siette J, Priebe S. To befriend or to be a friend: a systematic review of the meaning and practice of "befriending" in mental health care. *J Men Health*, 2016, 25(1):71-77.

17. Ozbay F, Johnson DC, Dimoulas E, Charney D, and Southwick S. Social Support and Resilience to Stress. From Neurobiology to Clinical Practice. *Psychiatry (Edgmont),* 2007; 4(5): 35–40.

18. Berkman L, Glass T, Brissette I, Seeman T. From social integration to health: Durkheim in the new millennium. *Social Science and Medicine*, 2000; 51; 6:843-57

19. Klug G, Klug G, Toner S, Fabisch K, Priebe S. Characteristics and motivations of volunteers providing one-to-one support for people with mental illness: a survey in Austria. *Social Psychiatry and Psychiatric Epidemiology*. 2018; 53:841-847

20 Toner S, Hickling LM, Pinto da Costa M, Cassidy M, Priebe S. Characteristics, motivations and experiences of volunteer befrienders for people with mental illness: a systematic review and narrative synthesis. *BMC Psychiatry*. 2018;18:378

21. Jenkinson CE, Dickens AP, Jones K, Thompson-Coon J, Taylor RS, Rogers M, Bambra CL, Lang I, Richards SH. Is volunteering a public health intervention? A systematic review and meta-analysis of the health and survival of volunteers. *BMC Public Health*. 2013; 13:773.

22. Heslop P. Good practice in befriending services for people with learning disability. *British Journal of Learning Disabilities*, 2005; 33:27-33.

23. Mead N, Lester H, Chew-Graham C, Gask L, Bower P et al. Effects of befriending on depressive symptoms and distress: systematic review and meta-analysis. *The British Journal of Psychiatry,* 2010, 196: 96-101.

For peer review only - http://bmjopen.bmj.com/site/about/guidelines.xhtml

24. Siette J, Cassidy M, Priebe S. Effectiveness of befriending interventions: a systematic review and meta-analysis. *BMJ Open*, 2017, 7:e014304.

25. Priebe S[1], Chevalier A[2], Hamborg T[3], Golden E[4], King M[5], Pistrang N[6]. Effectiveness of a volunteer befriending programme for patients with schizophrenia: randomised controlled trial. ;British Journal of Psychiatry. 2019; 4:1-7

26. Florides T. *Running a befriending project for people with learning disabilities. Final project report.* Circle Support; 2012 (accessed from http://www.mandbf.org/wp-content/uploads/2012/05/Running-a-Befriending-Project-for-Adults-with-Learning-Disabilites-The-Final-Report.pdf)

27. Stancliffe R J., Bigby C, Balandin S, Wilson N J, Craig D. Transition to retirement and participation in mainstream community groups using active mentoring: a feasibility and outcomes evaluation with a matched comparison group. *Journal of Intellectual Disability Research, 2015; 59*(8), 703–718.

28. Cuthill FM, Espie CA, Cooper SA. Development and psychometric properties of the Glasgow Depression Scale for people with a Learning Disability. Individual and carer supplement versions. *British Journal of Psychiatry*, 2003;182:347–53.

29. Wechsler D. Wechsler Abbreviated Scale of Intelligence, Second Edition (WASI-II). San Antonio, TX: NCS Pearson; 2011.

30. Priebe S, Pavlickova H, Eldridge S, Golden E, McCrone P, Ockenden N, Pistrang N, King M. Effectiveness of one-to-one volunteer support for patients with psychosis: protocol of a randomised controlled trial. *BMJ Open*, 2016, 6:e011582.

31. Dagnan D, Sandhu S. Social comparison, self esteem and depression in people with intellectual disability. *Journal of Intellectual Disability Research*, 1999: 372-379.

32. Skirrow P, Perry E. The Maslow Assessment of Needs Scales, 2009; Mersey Care NHS Trust, Liverpool.

33. World Health Organisation Quality of Life Assessment group. Development and general psychometric properties. Social Science and Medicine, 1998; 46: 1569-1585

34. Stancliffe RJ, Keane S. Outcomes and costs of community living: a matched comparison of group homes and semi-independent living. *Journal of Intellectual & developmental Disability,* 2000; 25: 281-305.

35. Lunsky Y, Benson B A, Reliability of ratings of consumers with mental retardation and their staff on multiple measures of social support. American Journal on Mental Retardation *1997;* 102, 280–4.

For peer review only - http://bmjopen.bmj.com/site/about/guidelines.xhtml

36. Baker PA. Measurement of community participation and use of leisure by service users with intellectual disabilities: The Guernsey community participation and leisure assessment (GCPLA). *Journal of Applied Research in Intellectual Disabilities*, 2000; 13:169-185

37. Rosenberg M. *Society and the adolescent self-image*. Princeton, NJ: Princeton University Press; 1965

38. Tennant R, Hiller L, Fishwick R, Platt S, Joseph S, Weich S et al. The Warwick-Edinburgh mental well-being scale (WEMWBS): development and UK validation. *Health and Quality of Life Outcomes*, 2007, 5(1): 63.

39. Russell, D., Peplau, L.A., & Cutrona, C.E. (1980). The revised UCLA Loneliness Scale:Concurrent and discriminant validity evidence. *Journal of Personality and Social Psychology,* 39, 472-480.

40. Morin D, Crocker AG, Beaulieu-Bergeron R, Caron J. Validation of the attitudes toward intellectual disability: ATTID questionnaire. *Journal of Intellectual Disability Research,* 2013; 57(3):268-78

41. Wille N, Badia X, Bonsel G, Burstrom K, Cavrini G, Devlin N, Egmar AC, Greiner W, Gusi N, Herdman M, Jelsma J, Kind P, Scalone L, Ravens-Sieberer U. Development of the EQ-5D-Y: a child-friendly version of the EQ-5D. *Quality of Life Research*, 2010; 19:875–886. https://euroqol.org/eq-5d-instruments/eq-5d-y-about/

42 Beecham J, Knapp M. Costing psychiatric interventions. In Measuring Mental Health Needs 2nd ed (ed G Thornicroft): 200–24. Gaskell, 2001

43. Moore GF, Audrey S, Barker M, Bond L, Bonell C, Hardeman W, Moore L, O'Cathain A, Tinati T, Wight D, Baird J. Process Evaluation of complex interventions: Medical Research Council Guidance. *British Medical Journal*, 2015; 350:h1258

For peer review only - http://bmjopen.bmj.com/site/about/guidelines.xhtml

SPIRIT
STANDARD PROTOCOL ITEMS: RECOMMENDATIONS FOR INTERVENTIONAL TRIALS

SPIRIT 2013 Checklist: Recommended items to address in a clinical trial protocol and related documents*

Section/item	Item No	Description	Addressed on page number
**Administrative information**			
Title	1	Descriptive title identifying the study design, population, interventions, and, if applicable, trial acronym	1
Trial registration	2a	Trial identifier and registry name. If not yet registered, name of intended registry	2
	2b	All items from the World Health Organization Trial Registration Data Set	
Protocol version	3	Date and version identifier	N/A
Funding	4	Sources and types of financial, material, and other support	14
Roles and responsibilities	5a	Names, affiliations, and roles of protocol contributors	1, 13/14
	5b	Name and contact information for the trial sponsor	13
	5c	Role of study sponsor and funders, if any, in study design; collection, management, analysis, and interpretation of data; writing of the report; and the decision to submit the report for publication, including whether they will have ultimate authority over any of these activities	14
	5d	Composition, roles, and responsibilities of the coordinating centre, steering committee, endpoint adjudication committee, data management team, and other individuals or groups overseeing the trial, if applicable (see Item 21a for data monitoring committee)	13

For peer review only

**Introduction**			
Background and rationale	6a	Description of research question and justification for undertaking the trial, including summary of relevant studies (published and unpublished) examining benefits and harms for each intervention	4, 5,
	6b	Explanation for choice of comparators	9
Objectives	7	Specific objectives or hypotheses	5,6
Trial design	8	Description of trial design including type of trial (eg, parallel group, crossover, factorial, single group), allocation ratio, and framework (eg, superiority, equivalence, noninferiority, exploratory)	7
**Methods: Participants, interventions, and outcomes**			
Study setting	9	Description of study settings (eg, community clinic, academic hospital) and list of countries where data will be collected. Reference to where list of study sites can be obtained	7
Eligibility criteria	10	Inclusion and exclusion criteria for participants. If applicable, eligibility criteria for study centres and individuals who will perform the interventions (eg, surgeons, psychotherapists)	6,7
Interventions	11a	Interventions for each group with sufficient detail to allow replication, including how and when they will be administered	8,9
	11b	Criteria for discontinuing or modifying allocated interventions for a given trial participant (eg, drug dose change in response to harms, participant request, or improving/worsening disease)	-
	11c	Strategies to improve adherence to intervention protocols, and any procedures for monitoring adherence (eg, drug tablet return, laboratory tests)	-
	11d	Relevant concomitant care and interventions that are permitted or prohibited during the trial	9
Outcomes	12	Primary, secondary, and other outcomes, including the specific measurement variable (eg, systolic blood pressure), analysis metric (eg, change from baseline, final value, time to event), method of aggregation (eg, median, proportion), and time point for each outcome. Explanation of the clinical relevance of chosen efficacy and harm outcomes is strongly recommended	9-11
Participant timeline	13	Time schedule of enrolment, interventions (including any run-ins and washouts), assessments, and visits for participants. A schematic diagram is highly recommended (see Figure)	Table 2

For peer review only - http://bmjopen.bmj.com/site/about/guidelines.xhtml

Sample size	14	Estimated number of participants needed to achieve study objectives and how it was determined, including clinical and statistical assumptions supporting any sample size calculations	6
Recruitment	15	Strategies for achieving adequate participant enrolment to reach target sample size	-

Methods: Assignment of interventions (for controlled trials)

Allocation:			
Sequence generation	16a	Method of generating the allocation sequence (eg, computer-generated random numbers), and list of any factors for stratification. To reduce predictability of a random sequence, details of any planned restriction (eg, blocking) should be provided in a separate document that is unavailable to those who enrol participants or assign interventions	7
Allocation concealment mechanism	16b	Mechanism of implementing the allocation sequence (eg, central telephone; sequentially numbered, opaque, sealed envelopes), describing any steps to conceal the sequence until interventions are assigned	7
Implementation	16c	Who will generate the allocation sequence, who will enrol participants, and who will assign participants to interventions	7
Blinding (masking)	17a	Who will be blinded after assignment to interventions (eg, trial participants, care providers, outcome assessors, data analysts), and how	10
	17b	If blinded, circumstances under which unblinding is permissible, and procedure for revealing a participant's allocated intervention during the trial	N/A

Methods: Data collection, management, and analysis

Data collection methods	18a	Plans for assessment and collection of outcome, baseline, and other trial data, including any related processes to promote data quality (eg, duplicate measurements, training of assessors) and a description of study instruments (eg, questionnaires, laboratory tests) along with their reliability and validity, if known. Reference to where data collection forms can be found, if not in the protocol	10-11
	18b	Plans to promote participant retention and complete follow-up, including list of any outcome data to be collected for participants who discontinue or deviate from intervention protocols	

For peer review only - http://bmjopen.bmj.com/site/about/guidelines.xhtml

Data management	19	Plans for data entry, coding, security, and storage, including any related processes to promote data quality (eg, double data entry; range checks for data values). Reference to where details of data management procedures can be found, if not in the protocol	13
Statistical methods	20a	Statistical methods for analysing primary and secondary outcomes. Reference to where other details of the statistical analysis plan can be found, if not in the protocol	12
	20b	Methods for any additional analyses (eg, subgroup and adjusted analyses)	N/A
	20c	Definition of analysis population relating to protocol non-adherence (eg, as randomised analysis), and any statistical methods to handle missing data (eg, multiple imputation)	N/A
**Methods: Monitoring**			
Data monitoring	21a	Composition of data monitoring committee (DMC); summary of its role and reporting structure; statement of whether it is independent from the sponsor and competing interests; and reference to where further details about its charter can be found, if not in the protocol. Alternatively, an explanation of why a DMC is not needed	
	21b	Description of any interim analyses and stopping guidelines, including who will have access to these interim results and make the final decision to terminate the trial	N/A
Harms	22	Plans for collecting, assessing, reporting, and managing solicited and spontaneously reported adverse events and other unintended effects of trial interventions or trial conduct	9/10
Auditing	23	Frequency and procedures for auditing trial conduct, if any, and whether the process will be independent from investigators and the sponsor	N/A
**Ethics and dissemination**			
Research ethics approval	24	Plans for seeking research ethics committee/institutional review board (REC/IRB) approval	13
Protocol amendments	25	Plans for communicating important protocol modifications (eg, changes to eligibility criteria, outcomes, analyses) to relevant parties (eg, investigators, REC/IRBs, trial participants, trial registries, journals, regulators)	

Consent or assent	26a	Who will obtain informed consent or assent from potential trial participants or authorised surrogates, and how (see Item 32)	7
	26b	Additional consent provisions for collection and use of participant data and biological specimens in ancillary studies, if applicable	N/A
Confidentiality	27	How personal information about potential and enrolled participants will be collected, shared, and maintained in order to protect confidentiality before, during, and after the trial	13
Declaration of interests	28	Financial and other competing interests for principal investigators for the overall trial and each study site	14
Access to data	29	Statement of who will have access to the final trial dataset, and disclosure of contractual agreements that limit such access for investigators	-
Ancillary and post-trial care	30	Provisions, if any, for ancillary and post-trial care, and for compensation to those who suffer harm from trial participation	N/A
Dissemination policy	31a	Plans for investigators and sponsor to communicate trial results to participants, healthcare professionals, the public, and other relevant groups (eg, via publication, reporting in results databases, or other data sharing arrangements), including any publication restrictions	13
	31b	Authorship eligibility guidelines and any intended use of professional writers	N/A
	31c	Plans, if any, for granting public access to the full protocol, participant-level dataset, and statistical code	N/A
**Appendices**			
Informed consent materials	32	Model consent form and other related documentation given to participants and authorised surrogates	included
Biological specimens	33	Plans for collection, laboratory evaluation, and storage of biological specimens for genetic or molecular analysis in the current trial and for future use in ancillary studies, if applicable	N/A

*It is strongly recommended that this checklist be read in conjunction with the SPIRIT 2013 Explanation & Elaboration for important clarification on the items. Amendments to the protocol should be tracked and dated. The SPIRIT checklist is copyrighted by the SPIRIT Group under the Creative Commons "Attribution-NonCommercial-NoDerivs 3.0 Unported" license.

For peer review only - http://bmjopen.bmj.com/site/about/guidelines.xhtml

For peer review only

BMJ Open

One to one befriending for people with intellectual disability and symptoms of depression: A protocol for a pilot randomised controlled trial.

Journal:	*BMJ Open*
Manuscript ID	bmjopen-2019-033989.R1
Article Type:	Protocol
Date Submitted by the Author:	20-Nov-2019
Complete List of Authors:	Ali , Afia; University College London, Division of Psychiatry<br>McKenzie, Emma; North East London NHS Foundation Trust Goodmayes Hospital<br>HASSIOTIS, ANGELA; ROYAL FREEand UNIVERSITY COLLEGE MEDICAL SCHOOL, PSYCHIATRY and BEHAVIOURAL SCIENCES<br>Priebe, Stefan; Barts and the London School of Medicine and Dentistry, University of London, Unit of Social and Community Psychiatry<br>Lloyd-Evans, Brynmor ; UCL, Division of Psychiatry<br>Omar, Rumana; University College London<br>Jones, Rebecca; UCL<br>Panca, Monica; University College London, Primary Care and Population Health<br>Fernandez, Vincent; Outward<br>Finning, Sally; Outward<br>Moore, Shirley; The Befriending Scheme<br>O'Connor, Danielle; The befriending Scheme<br>Roe, Christine; The Befriending Scheme<br>King, Michael; University College Medical School, Division of Psychiatry
**Primary Subject Heading**:	Mental health
Secondary Subject Heading:	Public health
Keywords:	Depression & mood disorders < PSYCHIATRY, QUALITATIVE RESEARCH, Delirium & cognitive disorders < PSYCHIATRY

SCHOLARONE™
Manuscripts

I, the Submitting Author has the right to grant and does grant on behalf of all authors of the Work (as defined in the below author licence), an exclusive licence and/or a non-exclusive licence for contributions from authors who are: i) UK Crown employees; ii) where BMJ has agreed a CC-BY licence shall apply, and/or iii) in accordance with the terms applicable for US Federal Government officers or employees acting as part of their official duties; on a worldwide, perpetual, irrevocable, royalty-free basis to BMJ Publishing Group Ltd ("BMJ") its licensees and where the relevant Journal is co-owned by BMJ to the co-owners of the Journal, to publish the Work in this journal and any other BMJ products and to exploit all rights, as set out in our licence.

The Submitting Author accepts and understands that any supply made under these terms is made by BMJ to the Submitting Author unless you are acting as an employee on behalf of your employer or a postgraduate student of an affiliated institution which is paying any applicable article publishing charge ("APC") for Open Access articles. Where the Submitting Author wishes to make the Work available on an Open Access basis (and intends to pay the relevant APC), the terms of reuse of such Open Access shall be governed by a Creative Commons licence – details of these licences and which Creative Commons licence will apply to this Work are set out in our licence referred to above.

Other than as permitted in any relevant BMJ Author's Self Archiving Policies, I confirm this Work has not been accepted for publication elsewhere, is not being considered for publication elsewhere and does not duplicate material already published. I confirm all authors consent to publication of this Work and authorise the granting of this licence.

For peer review only

One to one befriending for people with intellectual disability and symptoms of depression: A protocol for a pilot randomised controlled trial.

Afia Ali, *[1] Emma McKenzie [2], Angela Hassiotis [1], Stefan Priebe [3], Brynmor Lloyd-Evans [1], Rumana Omar [4], Rebecca Jones [1,5], Monica Panca [5], Vincent Fernandez [6], Sally Finning[6], Shirley Moore [7], Danielle O'Connor [7] Christine Roe [7], Michael King [1]

[1]Division of Psychiatry,

University College of London, UK

afia.ali@ucl.ac.uk

michael.king@ucl.ac.uk

b.lloyd-evans@ucl.ac.uk

a.hassiotis@ucl.ac.uk

reb.jones@ucl.ac.uk

[2] Research and Development Office,

Goodmayes Hospital, North East London NHS Foundation Trust, UK

Emma.mckenzie@nelft.nhs.uk

[3]Unit for Social and Community Psychiatry, (WHO Collaborating Centre for Mental Health Services Development),

Queen Mary University of London, UK

s.priebe@qmul.ac.uk.

[4] Department of Statistical Science

University College London, UK

r.omar@ucl.ac.uk

[5] PRIMENT Clinical Trials Unit,

University College London, UK

reb.jones@ucl.ac.uk

For peer review only - http://bmjopen.bmj.com/site/about/guidelines.xhtml

m.panca@ucl.ac.uk

[6]Outward, Newlon House

4 Daneland Walk, Hale Village, London,

N17 9FE

VFernandez@outward.org.uk

sfinning@outward.org.uk

[7] The Befriending Scheme,

1, The Croft, Sudbury,

Co10 1HN

danielle.oconnor@thebefriendingscheme.org.uk

christine.roe@thebefriendingscheme.org.uk

shirley.moore@thebefriendingscheme.org.uk

* Corresponding author

Afia Ali

Division of Psychiatry,

University College of London,

6th Floor, Maple House,

149 Tottenham court Road,

W1T 7NF

Email: afia.ali@ucl.ac.uk

Telephone: 0207 679 9334

Word count: 4919

For peer review only

Abstract

Introduction

People with intellectual disability (ID) are more likely to experience loneliness and have smaller social networks, which increases vulnerability to depression. Befriending may reduce depressive symptoms in other populations but randomised controlled trials (RCTs) have not been carried out in this population. This pilot study aims to assess the acceptability and feasibility of carrying out a full RCT of one to one befriending by volunteers for people with ID, compared to an active control group.

Methods and Analysis

The trial aims to recruit 50 participants with ID. Participants in the intervention arm will receive weekly visits from a volunteer over six months. Community befriending schemes will recruit, train, supervise volunteers and match them to individuals with ID. Both groups will receive a booklet about local activities and have access to usual care. Health and social outcomes will be measured at the end of the intervention and 6 months follow up. The following outcomes will be assessed: 1. recruitment and retention of individuals with ID and volunteers in the trial; 2. adverse events related to the intervention; 3. the acceptability of the intervention; 4. whether the intervention is delivered as intended; 5. changes in health and social outcomes and 6. the feasibility of carrying out a cost effectiveness analysis in a full trial. Qualitative data from participants, volunteers, staff and carers will identify barriers and facilitators of a future full trial.

Ethics and Dissemination

The study has been approved by the London- City and East Research Ethics Committee (reference 18/LO/2188). The findings will be presented at conferences and published in a peer reviewed journal and the National Institute of Health Research journals library. A public engagement seminar will be held at the end of the study aimed at key stakeholders.

Trial registration Number: ISRCTN63779614

Strengths and Limitations of the study

- This is the first pilot randomised controlled trial of one to one befriending in people with ID.
- This pilot study will examine the feasibility and acceptability of carrying out a future randomised controlled trial of befriending compared to an active control group.
- Community befriending services will be responsible for all aspects of the delivery of intervention
- Availability of resources within befriending services may influence the delivery of the intervention
- The findings will inform whether a full trial is feasible and what modifications would need to be made to overcome potential barriers.

For peer review only - http://bmjopen.bmj.com/site/about/guidelines.xhtml

Introduction

Intellectual disability (ID) is a life-long condition characterised by impaired cognitive and adaptive functioning, arising before the age of 18 (1). The UK prevalence of ID is 1-2% (2). People with ID have the same or higher prevalence of depression than the general population, but are more likely to experience chronic depression (3-4). They have greater exposure to social disadvantage (5), experience social exclusion because of stigma (6), have markedly smaller social networks (7-8) and a higher prevalence of loneliness compared to the general population (9). These factors have been associated with depressive symptoms in this group (10-13).

Conceptualisation of befriending

Befriending is “a relationship between two or more individuals, initiated, supported and monitored by an agency. The relationship is non-judgmental, mutual, purposeful, and there is a commitment over time” (14). Key attributes are that it is a one to one friendship-like relationship, it is an organised intervention, and that there is a negotiation of power (15). There is a wide variation in the concept and practice of befriending (16). At one extreme, befriending is very close to a friendship, characterised as being reciprocal and is delivered by lay volunteers, and at the other end it is a professional and therapeutic relationship, focused on the befriendee attaining goals and aspirations (mentoring). Most types of befriending relationships lie midway on this spectrum. Schemes are usually run by voluntary organisations in the community and offer training, supervision and on-going support to volunteers (16).

Befriending aims to help people who are lonely, isolated and have limited opportunities for social participation by increasing social and emotional support and by enhancing social networks and community participation. The causal mechanisms of befriending are unclear but social support is thought to be important. Social support may act as a buffer to stress and it may mediate genetic and environmental vulnerabilities to depression through its effects on neurobiological factors and other psychosocial factors (e.g. coping strategies) (17)The Befriender may enhance social support and link the befriendee into social activities, which may be sustainable outside of, and beyond the end of the befriending relationship, which may result in longer term benefits. Befriending may also improve health outcomes thorough its effects on social networks (18).

There are also potential benefits for befrienders. Motivation for befriending often includes a desire for “giving” something back to the community (e.g. helping others) and “getting” something in return (e.g. acquiring new skills or meeting new people) (19, 20)). Positive benefits reported by befrienders include increased self-esteem and confidence, feeling that they have gained a genuine friend themselves and improved attitudes towards people with mental illness (19). Volunteering in general has beneficial effects on depression, psychological wellbeing and life satisfaction, and is associated with lower risk of mortality, although the causal mechanisms for these associations are unclear (21).

However, the benefits of befriending may be short-lived and people with ID have reported feeling distressed following the termination of their befriending relationship (22). Other risks include the emotional turbulence that is associated with a natural friendship, or harmful

For peer review only - http://bmjopen.bmj.com/site/about/guidelines.xhtml

effects if the befriender is not adequately trained or supervised. There may also be undue burden placed on the befriender to take on excessive responsibility (16).

Effectiveness of befriending

One meta-analysis found that befriending in people with mental or physical health problems, (delivered by social and health care professionals as well as lay volunteers) had a statistically significant but modest effect on reducing symptoms of depression when compared to no treatment or treatment as usual in both the short and long term (23). Another systematic review examined a range of health and social outcomes in studies where befriending was delivered by volunteers only (24). Befriending was associated with better patient reported outcomes when all primary outcomes were combined but the effect size was small (24). However, in contrast to the previous review, there was limited evidence for the effectiveness of befriending on individual outcomes such as depression, loneliness or quality of life when the studies were combined (24). A recently published randomised controlled trial (RCT) of befriending by volunteers in people with psychosis provides further evidence for the potential beneficial effects of befriending (25). Participants in the intervention arm had significantly more social contacts at the end of the 12 month intervention and at six months follow up, suggesting that befriending may help to reduce social isolation in this is group.

The effectiveness of befriending in people with ID has not been evaluated in a randomised trial. An unpublished single arm feasibility study of one to one befriending by volunteers, conducted by a voluntary organisation, (26) recruited 24 volunteers, of which 15 were matched with an individual with ID. Positive change was reported in 60% of individuals with ID; 53% reported a decrease in isolation and 40% reported an increase in confidence. One Australian study examined the feasibility of using "active mentoring", whereby members of existing community groups were trained to act as mentors to older adults with ID, in order to provide social support and to encourage participation in community groups (27). The intervention comprised 29 individuals receiving the intervention and a matched comparison group. The participants in the intervention reported better social satisfaction compared to the comparison group but there were no significant changes in the other outcomes.

Given the dearth of studies examining befriending in people with ID there is a clear rationale for carrying out a pilot study prior to a full randomised controlled trial.

Aims and Objectives

The main aim of the study is to determine the feasibility and acceptability of a full scale randomised controlled trial of one to one befriending by volunteers for people with ID in addition to usual care, compared to an active control arm.

The objectives are to:

- examine the recruitment and retention of individuals with ID and volunteers in the trial and the number of successfully matched pairs within the six month study recruitment period
- Record any negative consequences/adverse effects of befriending
- Measure the extent to which the intervention is delivered as intended by volunteers and the befriending schemes

For peer review only - http://bmjopen.bmj.com/site/about/guidelines.xhtml

- Examine the acceptability of the intervention and study procedures by exploring the views of individuals, volunteers, carers and befriending services.
- Examine changes in health and social outcomes by carrying out exploratory analyses of the impact of befriending on depressive symptoms measured by the Glasgow Depression Scale (GDS (28)) at 12 months and other outcomes (psychological distress, self-esteem, loneliness, quality of life, and social participation) at 6 and 12 months post randomisation.
- Carry out exploratory analyses of the impact of befriending on volunteers' wellbeing, self-esteem, loneliness and attitudes towards people with intellectual disability at 6 and 12 months.
- Estimate the sample size required and determine the final trial design for a full scale randomised controlled trial.
- Assess the feasibility of collecting data that would inform a future analysis of cost effectiveness.

Methods

Design

This is a two arm, parallel group, researcher blind pilot RCT with 1:1 allocation. Fifty participants with ID who are eligible for the study will be randomly allocated to either the intervention arm (one to one befriending by a volunteer) or an active control arm. Both groups will have access to usual care and a booklet of local resources. The duration of the intervention will be 6 months. Outcome assessments will be carried out at baseline, post intervention and at six months follow-up. A process evaluation, based on mixed methods, will be carried out to examine the delivery and adherence to intervention, and stakeholder views on the acceptability of the intervention and barriers and facilitators that may affect the implementation of a full scale trial.

Sample Size

We do not have any estimates of the number of people with ID who are eligible and are likely to consent to taking part in the trial. If we approach 50 participants who are eligible to take part, this will allow us to estimate an expected recruitment rate of 80% (40 people), with a 95% confidence interval (CI) of 68.9% to 91.1%. A sample size of 40 recruited people with ID would allow us to estimate a 30% drop out rate in the trial with a 95% CI of 25.7% to 54.3 %. The recruitment period is 6 months. There are two participating befriending services and therefore we will need to recruit 3.3 participants with ID per month at each site.

Participants

Inclusion Criteria

We will include: individuals with ID aged 18 or over, who have mild or moderate intellectual disability (IQ of 35-60) assessed using the Wechsler Abbreviated Scale of Intelligence (WASI-II; (29)); should not be attending college/education or a day service for three or more days a week; will have a score of 5 or more on the Glasgow Depression Scale for People with Learning Disability (GDS-LD (28)), indicating the presence of depressive symptoms but

For peer review only - http://bmjopen.bmj.com/site/about/guidelines.xhtml

do not need to have a clinical diagnosis of depression. Participants will need to be able to speak English and provide informed consent.

Volunteers aged 18 or over, who can agree to being available once a week for at least one hour over a period of six months. Volunteers do not need to have any prior experience of supporting people with ID.

Exclusion Criteria

Individuals with ID will be excluded if they have limited communication and comprehension and therefore would not be able to complete the questionnaires.

Volunteers will be excluded if they have a criminal record (any documented offence due to the vulnerability of this population) recorded on their Disclosure and Barring Service (DBS) check or are unable to provide two references or have unsuitable references.

Recruitment

Befriending services

Two befriending services ("Outward", based in Hackney, North London and "the befriending scheme" in Suffolk) will be taking part in the study and will be responsible for overseeing the befriending intervention. Both services have experience supporting befriending relationships with people with ID including matching individuals with volunteers and monitoring relationships.

Participants with ID

Participants with ID will be recruited from existing and new referrals to the befriending services or recruited from the caseload of clinicians working in community learning disability services in North London and Suffolk. Volunteer coordinators and clinicians will screen referrals and caseloads for participants who may be eligible for the study and will make the initial approach. If the individual is interested in the study, and consents to his/her details being passed on to the research team, a referral form will be completed and emailed to the research team. The research assistant will then contact the individual and carry out an eligibility assessment. If the individual is eligible, they will need to provide written consent before completing the baseline assessment.

Volunteers

The befriending services will advertise and recruit volunteers through newspaper advertisements, befriending and job websites, social media and recruitment events at colleges and universities. Volunteers will be recruited directly from the study website hosted by the UCL Division of Psychiatry and through the Division's twitter account. Interested volunteers will complete an application form and will be invited to an informal interview to assess their suitability and motivation for taking part in the scheme. A DBS check will be completed to ensure that they have no criminal records and references will be obtained. People with ID are potentially a vulnerable group and therefore volunteers with any previous offence, including minor offences, will not be included in the study. Successful candidates

For peer review only - http://bmjopen.bmj.com/site/about/guidelines.xhtml

will be invited to take part in the study. They will receive an information sheet and asked to sign a consent form, followed by completion of the baseline assessment.

Randomisation

Randomisation will be carried out after the baseline assessments have taken place. Participants will be randomised into the study by an unblinded member of the research team who will enter the patients' details into a web based randomisation system, which is hosted on a secure server by Sealed Envelope. This system will randomly allocate the participant to either the intervention or control arm. The unblinded researcher will notify the befriending service and participants with ID of their allocation.

Randomisation will be blocked using randomly varying block sizes, stratified by centre. The allocation schedule will be concealed through the use of this central web-based randomisation service. The randomisation protocol will be created by the trial statistician and the set up of the service will be overseen by the Priment Clinical Trials Unit.

Intervention group

Participants with ID will meet with the volunteering coordinator to obtain information about their hobbies, interests and activities they would like support to participate in. Based on this information, participants with ID will be matched to a volunteer that can accommodate the person's interests.

i. The befriending intervention

The befriending intervention has been adapted from the existing models used by the two participating befriending services and from other studies of befriending (26, 30). The purpose of the befriending relationship will be to provide friendship and emotional support, and to assist the individual to access activities in the community that they may be unable to do alone. As participants become more confident, they will be encouraged to access activities in the community on their own, in order to promote sustained social activities beyond the befriending relationship.

The volunteer (befriender) and person with ID will meet once a week for at least one hour, for six months, although some breaks are anticipated due to holidays or illness. They will receive a booklet detailing local activities and amenities, which they can use to plan activities. The emphasis will be on assisting the individual to make choices about the activities that they wish to pursue. The volunteer is not expected to carry out personal care, administer medication or accompany the individual to medical appointments. Contacts by phone/social media can take place in addition to face to face contacts. The pair can spend some sessions in the person's home but this should not exceed 50% of the total number of sessions. Sessions may take place during evenings/weekends depending on the pair's availability. They will keep a record of their activities in a structured log that will be provided (whether they attended each session, reasons for cancellation, what they did in each session and duration of activity), and record of other types of contact. Volunteers will be reimbursed travel expenses but other expenses will need to be agreed with the befriending service. Participants with ID will not be reimbursed travel expenses or the costs of activities.

ii. Introduction and monitoring of the befriending relationship

For peer review only - http://bmjopen.bmj.com/site/about/guidelines.xhtml

The volunteering coordinator will arrange a face-to-face meeting where the pair will be introduced to each other. If they agree to continue, the pair will arrange to meet on their own; if they decide that the pairing is unsuitable, they will be re-matched. If the volunteer or individual with ID drops out of the relationship once it has become established, attempts will be made to re-match them. Volunteers may be matched to more than one participant with ID.

The volunteering coordinator will arrange a face-to-face meeting with the pair after six weeks and will maintain contact with each person by telephone/ face to face contact every four weeks thereafter to monitor the progress of the relationship. A further meeting will be held with the pair at the end of the six months to obtain general feedback about the befriending intervention, to discuss ending the relationship and to support the individual with ID with coming to terms with the ending. The pair may continue their relationship if they wish after the six month period but arrangements for monitoring the relationship will vary depending on the befriending service. Information will be obtained on any relationships continuing beyond six months and the monitoring that has been provided.

iii. Training and supervision of volunteers

The volunteers will attend training delivered face-to-face and as e-learning. The training will cover: the benefits of befriending and issues related to confidentiality and lone working; advice on how to plan meetings effectively; health and safety; safeguarding ; learning disability awareness, and professional boundaries, which covers dealing with sensitive issues, ending relationships and expectations of the role of the volunteer. Volunteers will also receive slides and a manual developed by the research team, with information about the study and additional information about communication, challenging behaviour and mental health problems.

Volunteers will have access to group or individual supervision delivered face to face or over the phone, once a month, by the volunteer coordinator, which will address issues that may have arisen from the relationship, for example, concerns about mental health or behaviour.

iv. Training and support for befriending services

Participating befriending services will complete Good Medical Practice (GCP) Training and undergo training on the study research processes and procedures as part of the site initiation visit. Volunteer coordinators will receive support from the research team through regular email and telephone contact and they will attend Trial Management Group (TMG) meetings.

Control Group

Participants in the control arm will receive the activities booklet. They will meet with a member of the research team who will discuss the booklet with them (and their carer, if present) and encourage them to engage with activities. This is to control for the effects of participants in the treatment arm receiving more information about local activities.

For peer review only - http://bmjopen.bmj.com/site/about/guidelines.xhtml

Both the control and intervention arms will also have access to "usual care". This will include access to multidisciplinary input from community intellectual disability services. Participants can continue to take their usual medication and can access other community and hospital health services and day services. Information about usual care will be collected as part of the baseline assessment.

Outcomes

i. Recruitment and retention of participants:
We will examine the proportion of participants with ID recruited from among those eligible; and the proportion of volunteers recruited from among people expressing an interest over a six months period; the proportion of participants with ID who are successfully matched with a volunteer; the proportion of participants with ID and volunteers who drop out of the intervention arm and the proportion of participants and volunteers who complete subsequent follow up assessments.

ii. Adverse events
Adverse events will be collected at each follow up assessment using open-ended questions and will also be reported directly to the Chief Investigator by the befriending services, including concerns about safeguarding. Volunteers will follow a protocol if they have concerns about the participant's mental health, which will involve informing the volunteer coordinator, who will in turn notify the CI and the referring clinician or a health professional involved in the person's care. If a serious incident (e.g. self -harm, challenging behaviour) occurs out of hours, the carer will be notified and emergency services will be contacted if appropriate. All adverse events will be recorded in the medical records and CRFs. Serious adverse events (e.g. safeguarding concerns, hospitalisation) will be recorded in the Serious Adverse Effects (SAE) log.

iii. Acceptability of the intervention
This will be informed by data on retention/ drop out of volunteers and participants, the extent of engagement with the intervention by participants and volunteers (based on number of sessions attended) and qualitative data obtained from volunteers, participants with ID, carers of people with ID and staff from the befriending service.

i*v. Adherence to the intervention*
Data will be collected on volunteer training, uptake of supervision and the frequency of monitoring checks carried out by volunteer coordinators from routine records at each site in order to assess fidelity to the intervention by the befriending services. Structured logs provided by volunteers will be analysed to assess fidelity to the intervention by volunteers. We will examine: a) how many sessions were attended by each volunteer, reasons for non-attendance, and how many attended at least 10 sessions during the 6-month intervention period; and b) for how many participants the minimum threshold of at least 50% of meetings being outside the participant's home was achieved.

v. Depression and other health and social outcome measures in participants with ID

For peer review only - http://bmjopen.bmj.com/site/about/guidelines.xhtml

All the health and social outcome measures have been validated in people with ID and will be assessed with the participant at baseline, at the end of the intervention and at six months follow up. (see table 1: schedule of assessments). Assessments will be carried out by a research assistant (RA) blind to group allocation and will take place face to face at the befriending service or the participants' homes.

- Depressive symptoms will be measure using the GDS-LD (28). Scores at 12 months will be the main health outcome of interest.
- Self-esteem will be measured using the adapted Rosenberg self-esteem scale for people with intellectual disabilities (31)
- Quality of life will be measured using the Maslow Assessment of Needs Scale-Learning Disability (MANS-LD; (32)) and five items from the adapted World Health Organisation Quality of Life questionnaire (WHO-QOL-8; (33)).
- Loneliness and social satisfaction will be measured using the Modified Worker Loneliness Questionnaire (MWLQ (34),
- Social support will be measured using the Social Support Self Report for intellectually disabled adults (SSSR) (35)
- Social participation will be measured at using the Guernsey Community Participation and Leisure Assessment (GCPLA) (36).

vi. Volunteer outcome measures

The following outcome assessments will be carried out in volunteers at baseline (prior to matching) and post intervention (six months after the baseline) and at six months follow-up (see table 2 schedule of assessments):

- Self-esteem will be measured using the 10 item Rosenberg self-esteem scale (37)
- Psychological wellbeing and quality of life will be measured using the Warwick-Edinburgh mental wellbeing scale (WEMWBS; 38).
- Loneliness will be measured using the UCLA loneliness scale (39).
- Attitudes of volunteers will be assessed using the 67 item, Attitudes Towards Intellectual Disability Questionnaire (ATTID; 40)

vii. Feasibility of carrying out a cost effectiveness analysis

The preliminary health economic analysis will inform planning of future economic analyses, sources of data required and how best to collect these data. The following measures will be recorded at baseline, post intervention (six months after randomisation) and at six months follow-up.

- Health related quality of life will be measured using the EuroQol-Youth (EQ-5D-Y) (41). We will assess the feasibility of using the EQ-5D-Y to calculate Quality adjusted life years (QALYs). QALYs will be calculated using the EQ-5D-3 L tariff, as there are no value sets for the EQ-5 D-Y (42).
- Client Services Receipt Inventory (CSRI) for people with intellectual disabilities adapted for the study (43), completed with carers where possible, will assess the feasibility of collecting participant reported service use over the previous six months.

For peer review only - http://bmjopen.bmj.com/site/about/guidelines.xhtml

Process evaluation

A process evaluation will be undertaken, based on MRC guidance (44). The aim will be to examine whether the different components of the intervention were consistently followed; the extent to which volunteers delivered the intervention as intended; to understand the perceived value, benefits and unintended consequences of the intervention so that these are fully measured in a full trial, contextual factors influencing how the intervention was delivered; and to develop an understanding of the likely mechanisms of action of the intervention. We will interview 16 participants with ID after their 12 month follow up (8 per site) and carry out two focus groups at each site with 5-8 volunteers, staff from each befriending service, and 5-8 carers.. We will use purposive sampling in order to include people with a range of demographic characteristics (e.g. gender, age, ethnicity, living arrangements), and both participants and volunteers for whom the befriending relationship broke down, as well as people who completed the intervention. All respondents will be asked about what aspects of the intervention were perceived to be helpful, what aspects require modification and suggestions for improvement, views about trial procedures and the perceived barriers and facilitators to delivering a larger trial. The interviews and focus groups will be audio-taped and transcribed.

In order to understand how the befriending intervention was delivered, the location, content of the meetings and the range of activities undertaken will be described based on an analysis of structured log books. A framework will be developed to categorise different types of activity to enable different types of befriending support to be distinguished and quantified. Test procedures will be developed for collecting quantitative process data, which, in a future larger trial, could be used to explore the relationships between process variables and outcomes. Findings from the process evaluation will inform any necessary refinements to the study intervention or procedures.

Analysis

Feasibility outcomes such as the number of participants who were screened and eligible, the proportion of eligible participants with ID and interested volunteers recruited to the study, and the proportion successfully matched in befriending relationships, will be reported. The number (proportion) of drop-outs of volunteers and participants from the intervention arm and from both arms at each follow up assessment, will also be reported, including reasons why where possible. This information will be presented in a Consort diagram describing the flow of participants through the study. (http://www.consort-statement.org/).

The characteristics and outcomes of participants by trial arm will be summarised using means and standard deviations or medians and inter-quartile ranges for continuous variables and count and percentages for categorical variables. Appropriate regression models depending on the type of outcome, adjusted for baseline values and centre will be used to estimate the intervention effect on health and social outcomes where possible. The results will be presented as estimates with 95% CI. All analyses will be carried out as randomised with available data (intention to treat principle) and will be used to inform the definitive trial. The characteristics of patients with missing outcome data will be investigated.

For peer review only - http://bmjopen.bmj.com/site/about/guidelines.xhtml

Economic Evaluation

We will assess the feasibility of gathering information for a cost-effectiveness analysis for a full RCT including testing the suitability of calculating quality-adjusted life years (QALYs) using the EQ-5D-Y. We will calculate the costs of delivering the befriending intervention (training, supervision and expenses), which will be obtained from each participating befriending service. Mean resource use/ costs (standard deviation (SD)) per participant at baseline and follow-up, based on completion of the CSRI, will be presented.

Qualitative analysis

Transcripts from the focus groups and interviews will be analysed by the study team using thematic analysis supported by computer software (NVivo 9). The analytic strategy will focus on identifying barriers and facilitators to implementing the intervention successfully, its benefits to participants and mechanisms of effect. Analysis will also allow consideration of themes that arise more inductively from the data. Validity will be enhanced by a collaborative analytic strategy involving members of the research team and the advisory group who will meet together to review the coding framework and agree on the themes.

Patient and Public Involvement

Volunteers, people with ID and befriending schemes were involved in the design of the study. During the study, there will be advisory groups comprising carers, current volunteers and individuals with ID who will provide advice about the study information sheets, consent forms, topic guides for the qualitative interviews and focus groups, results of the study findings and the final study report. Members will be invited to be part of the TMG and will be involved in carrying out the qualitative interviews and focus groups as part of the process evaluation. They will receive training and support for this role. We will also invite members to participate in the public engagement seminar at the end of the study.

Ethics, governance and dissemination

Amendments to the study protocol and documents will be approved by the sponsor (UCL) and the Ethics Committee. Priment Clinical Trials Unit (CTU) will ensure that the trial procedures meet the requirements of Good Clinical Practice (GCP) and will complete dataquality assurance checks. The RA will have experience working with people with ID and will receive training on assessing capacity and carrying out assessments. Supervision of the RA will be provided by the Chief Investigator (AA). The study team, co-applicants, PPI advisors and the CTU will be part of the TMG and will meet every 10-12 weeks to discuss the progress of the study. An independent trial steering committee will provide overall supervision of the trial and will report to the funders. Data confidentiality will be maintained by assigning participants study identification numbers and data will be entered anonymously and stored in a secure web-based database.

The findings of the study will be presented at conferences and published in an open access peer reviewed journal as well as the National Institute of Health Research (NIHR) Public Health Research Journal. A public engagement seminar targeted at relevant stakeholders will take place at the end of the study to discuss the study findings and implications. A summary report will be developed for participants and volunteers taking part in the study and will be published on the study website.

For peer review only - http://bmjopen.bmj.com/site/about/guidelines.xhtml

Study timeline

Recruitment of participants began in April 2019. There will be a six month recruitment period, a further 12 months to complete the follow up assessment and three months to complete the process evaluation, analyse the results and write up the study findings.

Discussion

This is the first pilot RCT of one to one befriending, monitored by community befriending services, in people with ID. The trial will provide data on whether a full trial will be feasible, in terms of recruitment and retention of volunteers and people with ID and data on potential beneficial and adverse effects, acceptability and the extent to which the intervention is delivered as intended by volunteers and the befriending services. It will help to inform modifications that need to be made to enable a future trial to overcome barriers and challenges that may be encountered in this pilot.

There is currently limited evidence supporting the choice of a primary health outcome but the use of depressive symptoms is supported by one systematic review (24). This pilot study will help to determine whether measuring depressive symptoms is the most appropriate primary outcome for a future trial.

One of the main challenges of this study will be to ensure that the intervention and the recruitment, training and supervision of volunteers are carried out according to the trial protocol as these aspects vary between befriending services, and was an issue in a recent trial of befriending in people with psychosis (25) While both befriending services have committed to the study, there are resource implications for these services as the intervention costs are not covered by the research funding. External factors such as funding cuts to these services could influence the delivery of the intervention and the success of the trial. The process data will allow us to examine whether a future trial would need to be more pragmatic and whether the befriending intervention should permit more flexibility.

Author Contributions: AA is the chief investigator and is responsible for the overall study design and conduct. AA, SP, MK, AH, BL-E and RO have contributed to the design of the study and methodology. SF, VF, DO, CR, SM are responsible for designing and delivering the intervention. EM will carry out the data collection. RO, RJ and MP have contributed to the analysis plan. All the authors have reviewed and approved the manuscript.

Funding statement: The study/ project is funded by the National Institute of Health Research (NIHR) (Public Health Research Funding Stream (project reference: 16/122/57). The views expressed are the views of the authors and are not necessarily those of the NIHR of the Department of Health and Social Care.

Competing Interests statement: There are no conflicting interests to declare.

For peer review only - http://bmjopen.bmj.com/site/about/guidelines.xhtml

Table 1: Schedule of assessments in participants with ID

Assessment/Outcome	Screening and baseline assessment	Post intervention assessment	6 months follow up
Eligibility confirmation:<br>WASI-II<br>GDS-LD	x		
Informed consent	x		
Socio-demographic questionnaire	x		
Adapted Rosenberg self-esteem scale	x	x	x
MANS-LD	x	x	x
WHO-QOL-8	x	x	x
MWLQ	x	x	x
SSSR	x	x	x
GCPLA	x	x	x
EQ-5D-Y	x	x	x
CSRI	x	x	x
Adverse effects review	x	x	x
Concomitant medication	x	x	x
Semi-structured interview (optional)			x

WASI-II (Wechsler Abbreviated Scale of Intelligence, 2nd edition); GDS-LD (Glasgow Depression Scale for People with Learning Disability); MANS-LD (Maslow Assessment of Needs Scale learning Disability); WHO-QOL-8 (World Health Organisation Quality of Life questionnaire); MWLQ (Modified Worker Loneliness Questionnaire; SSSR (Social Support Self Report for intellectually disabled adults); GCPLA (Guernsey Community Participation and Leisure Assessment); EQ-5D-Y (EuroQol-Youth); CSRI (Client Services Receipt Interview).

For peer review only - http://bmjopen.bmj.com/site/about/guidelines.xhtml

Table 2: Schedule of assessments with volunteers

Assessment/ outcome	Screening and baseline	Post intervention assessment	6 months follow up
Eligibility check:<br>Informal interview;<br>DBS check<br>references	x		
Informed consent	x		
Rosenberg self-esteem scale	x	x	x
WEMWBS	x	x	x
UCLA Loneliness Scale	x	x	x
ATTID	x	x	x
Focus group (optional)			x

WEMWBS Warwick- Edinburgh mental wellbeing scale; ATTID (Attitudes Towards Intellectual Disability Questionnaire)

For peer review only

For peer review only - http://bmjopen.bmj.com/site/about/guidelines.xhtml

References

1. *American Psychiatric Association. Diagnostic and Statistical Manual of Mental Disorders (5th ed.).* Arlington, VA: American psychiatric Publishing; 2013.

2. Emerson E, Hatton C, Robertson J, Roberts H, Baines S, Glover Gyles. *People with learning disabilities in England 2010: services and supports.* Improving Health and Lives Learning Disability Observatory, 2010 (accessed from. http://www.improvinghealthandlives.org.uk/uploads/doc/vid_9244_IHAL2011-02PWLD2010.pdf

3. Cooper S-A, Smiley E, Morrison J, Williamson A, Allan L. An epidemiological investigation of affective disorders with a population-based cohort of 1,023 adults with intellectual disabilities. *Psychological Medicine*, 2007;37:873–82.

4. Richards M, Maughan B, Hardy R, et al. Long-term affective disorder in people with mild learning disability. *British Journal of Psychiatry*, 2001;179:523-7.

5. Emerson E and Einfeld SL. *Challenging Behaviour*. Cambridge: Cambridge University Press; 2011

6. Ali A, Hassiots A, Strydom A, King M. Self stigma in people with intellectual disabilities and courtesy stigma in family carers: A systematic review. *Research in Developmental Disabilities*, 2012; 33: 2122-2140

7. Verdonschot M.M.L,deWitte L.P, Reichrath E, Buntinx W.H.E, &Curfs L.N.G. Impact of environmental factors on community participation of persons with an intellectual disability: A systematic review. *Journal of Intellectual Disability Research*, 2009; 53(1), 54–64.

8. Hill RA & Dunbar RIM. Social network size in humans. *Human Nature*, 2003; 14(1):53-72.

9. Petroutsou A, Ali A, Hassiotis A. Loneliness in people with intellectual and developmental disorders (IDD) across the life span: a systematic review of prevalence and interventions. *Journal of Applied Research in Intellectual Disability* (published online Feb 2018).

10. Ali A, King M, Strydom S, Hassiotis A. Self-reported stigma and symptoms of anxiety and depression in people with intellectual disabilities: Findings from a cross sectional study in England. *Journal of Affective Disorders*, 2015, 187:224-231

11. Emerson E. Self reported exposure to disablism is associated with poor self reported health and wellbeing among adults with intellectual disabilities in England: a cross sectional survey. *Public Health*, 2010; 124, 682-689

12. Ailey S H, Miller A M, Heller T, & Smith E V. Evaluating an interpersonal model of depression among adults with Down syndrome. *Research and Theory for Nursing Practice*,

For peer review only - http://bmjopen.bmj.com/site/about/guidelines.xhtml

2006; *20*(3), 229-246.

13. Merrick, J., Merrick, E., Lunsky, Y., & Kandel, I. A review of suicidality in persons with intellectual disability. *The Israel Journal of Psychiatry and Related Sciences*, 2006; *43*(4), 258-264.

14. Dean, J. & Goodlad, R.*Supporting Community Participation: the role and impact of befriending.* Brighton: Pavilion Publishing & Joseph Rowntree Foundation, 1998.

15. Balaam MC. A concept analysis of befriending. J Adv Nurs, 2015, 71(1): 24-34.

16. Thompson R, Valenti E, Siette J, Priebe S. To befriend or to be a friend: a systematic review of the meaning and practice of "befriending" in mental health care. *J Men Health*, 2016, 25(1):71-77.

17. Ozbay F, Johnson DC, Dimoulas E, Charney D, and Southwick S. Social Support and Resilience to Stress. From Neurobiology to Clinical Practice. *Psychiatry (Edgmont),* 2007; 4(5): 35–40.

18. Berkman L, Glass T, Brissette I, Seeman T. From social integration to health: Durkheim in the new millennium. *Social Science and Medicine*, 2000; 51; 6:843-57

19. Klug G, Klug G, Toner S, Fabisch K, Priebe S. Characteristics and motivations of volunteers providing one-to-one support for people with mental illness: a survey in Austria. *Social Psychiatry and Psychiatric Epidemiology*. 2018; 53:841-847

20 Toner S, Hickling LM, Pinto da Costa M, Cassidy M, Priebe S. Characteristics, motivations and experiences of volunteer befrienders for people with mental illness: a systematic review and narrative synthesis. *BMC Psychiatry*. 2018;18:378

21. Jenkinson CE, Dickens AP, Jones K, Thompson-Coon J, Taylor RS, Rogers M, Bambra CL, Lang I, Richards SH. Is volunteering a public health intervention? A systematic review and meta-analysis of the health and survival of volunteers. *BMC Public Health*. 2013; 13:773.

22. Heslop P. Good practice in befriending services for people with learning disability. *British Journal of Learning Disabilities*, 2005; 33:27-33.

23. Mead N, Lester H, Chew-Graham C, Gask L, Bower P et al. Effects of befriending on depressive symptoms and distress: systematic review and meta-analysis. *The British Journal of Psychiatry,* 2010, 196: 96-101.

24. Siette J, Cassidy M, Priebe S. Effectiveness of befriending interventions: a systematic review and meta-analysis. *BMJ Open*, 2017, 7:e014304.

For peer review only - http://bmjopen.bmj.com/site/about/guidelines.xhtml

25. Priebe S[1], Chevalier A[2], Hamborg T[3], Golden E[4], King M[5], Pistrang N[6]. Effectiveness of a volunteer befriending programme for patients with schizophrenia: randomised controlled trial. ;British Journal of Psychiatry. 2019; 4:1-7

26. Florides T. *Running a befriending project for people with learning disabilities. Final project report.* Circle Support; 2012 (accessed from http://www.mandbf.org/wp-content/uploads/2012/05/Running-a-Befriending-Project-for-Adults-with-Learning-Disabilites-The-Final-Report.pdf)

27. Stancliffe R J., Bigby C, Balandin S, Wilson N J, Craig D. Transition to retirement and participation in mainstream community groups using active mentoring: a feasibility and outcomes evaluation with a matched comparison group. *Journal of Intellectual Disability Research, 2015; 59*(8), 703–718.

28. Cuthill FM, Espie CA, Cooper SA. Development and psychometric properties of the Glasgow Depression Scale for people with a Learning Disability. Individual and carer supplement versions. *British Journal of Psychiatry*, 2003;182:347–53.

29. Wechsler D. Wechsler Abbreviated Scale of Intelligence, Second Edition (WASI-II). San Antonio, TX: NCS Pearson; 2011.

30. Priebe S, Pavlickova H, Eldridge S, Golden E, McCrone P, Ockenden N, Pistrang N, King M. Effectiveness of one-to-one volunteer support for patients with psychosis: protocol of a randomised controlled trial. *BMJ Open*, 2016, 6:e011582.

31. Dagnan D, Sandhu S. Social comparison, self esteem and depression in people with intellectual disability. *Journal of Intellectual Disability Research*, 1999: 372-379.

32. Skirrow P, Perry E. The Maslow Assessment of Needs Scales, 2009; Mersey Care NHS Trist, Liverpool.

33. World Health Organisation Quality of Life Assessment group. Development and general psychometric properties. Social Science and Medicine, 1998; 46: 1569-1585

34. Stancliffe RJ, Keane S. Outcomes and costs of community living: a matched comparison of group homes and semi-independent living. *Journal of Intellectual & developmental Disability,* 2000; 25: 281-305.

35. Lunsky Y, Benson B A, Reliability of ratings of consumers with mental retardation and their staff on multiple measures of social support. American Journal on Mental Retardation *1997;* 102, 280–4.

36. Baker PA. Measurement of community participation and use of leisure by service users with intellectual disabilities: The Guernsey community participation and leisure

For peer review only - http://bmjopen.bmj.com/site/about/guidelines.xhtml

assessment (GCPLA). *Journal of Applied Research in Intellectual Disabilities*, 2000; 13:169-185

37. Rosenberg M. *Society and the adolescent self-image*. Princeton, NJ: Princeton University Press; 1965

38. Tennant R, Hiller L, Fishwick R, Platt S, Joseph S, Weich S et al. The Warwick-Edinburgh mental well-being scale (WEMWBS): development and UK validation. *Health and Quality of Life Outcomes*, 2007, 5(1): 63.

39. Russell, D., Peplau, L.A., & Cutrona, C.E. (1980). The revised UCLA Loneliness Scale:Concurrent and discriminant validity evidence. *Journal of Personality and Social Psychology,* 39, 472-480.

40. Morin D, Crocker AG, Beaulieu-Bergeron R, Caron J. Validation of the attitudes toward intellectual disability: ATTID questionnaire. *Journal of Intellectual Disability Research,* 2013; 57(3):268-78

41. Wille N, Badia X, Bonsel G, Burstrom K, Cavrini G, Devlin N, Egmar AC, Greiner W, Gusi N, Herdman M, Jelsma J, Kind P, Scalone L, Ravens-Sieberer U. Development of the EQ-5D-Y: a child-friendly version of the EQ-5D. *Quality of Life Research*, 2010; 19:875–886. https://euroqol.org/eq-5d-instruments/eq-5d-y-about/

42. EQ-5D-Y (Youth) About. https://euroqol.org/eq-5d-instruments/eq-5d-y-about/. Accessed November 2019.

43 Beecham J, Knapp M. Costing psychiatric interventions. In Measuring Mental Health Needs 2nd ed (ed G Thornicroft): 200–24. Gaskell, 2001

44. Moore GF, Audrey S, Barker M, Bond L, Bonell C, Hardeman W, Moore L, O'Cathain A, Tinati T, Wight D, Baird J. Process Evaluation of complex interventions: Medical Research Council Guidance. *British Medical Journal*, 2015; 350:h1258

For peer review only

STANDARD PROTOCOL ITEMS: RECOMMENDATIONS FOR INTERVENTIONAL TRIALS

SPIRIT 2013 Checklist: Recommended items to address in a clinical trial protocol and related documents*

Section/item	Item No	Description	Addressed on page number
**Administrative information**			
Title	1	Descriptive title identifying the study design, population, interventions, and, if applicable, trial acronym	1
Trial registration	2a	Trial identifier and registry name. If not yet registered, name of intended registry	2
	2b	All items from the World Health Organization Trial Registration Data Set	
Protocol version	3	Date and version identifier	N/A
Funding	4	Sources and types of financial, material, and other support	14
Roles and responsibilities	5a	Names, affiliations, and roles of protocol contributors	1, 13/14
	5b	Name and contact information for the trial sponsor	13
	5c	Role of study sponsor and funders, if any, in study design; collection, management, analysis, and interpretation of data; writing of the report; and the decision to submit the report for publication, including whether they will have ultimate authority over any of these activities	14
	5d	Composition, roles, and responsibilities of the coordinating centre, steering committee, endpoint adjudication committee, data management team, and other individuals or groups overseeing the trial, if applicable (see Item 21a for data monitoring committee)	13

For peer review only

For peer review only - http://bmjopen.bmj.com/site/about/guidelines.xhtml

**Introduction**			
Background and rationale	6a	Description of research question and justification for undertaking the trial, including summary of relevant studies (published and unpublished) examining benefits and harms for each intervention	4, 5,
	6b	Explanation for choice of comparators	9
Objectives	7	Specific objectives or hypotheses	5,6
Trial design	8	Description of trial design including type of trial (eg, parallel group, crossover, factorial, single group), allocation ratio, and framework (eg, superiority, equivalence, noninferiority, exploratory)	7
**Methods: Participants, interventions, and outcomes**			
Study setting	9	Description of study settings (eg, community clinic, academic hospital) and list of countries where data will be collected. Reference to where list of study sites can be obtained	7
Eligibility criteria	10	Inclusion and exclusion criteria for participants. If applicable, eligibility criteria for study centres and individuals who will perform the interventions (eg, surgeons, psychotherapists)	6,7
Interventions	11a	Interventions for each group with sufficient detail to allow replication, including how and when they will be administered	8,9, 10
	11b	Criteria for discontinuing or modifying allocated interventions for a given trial participant (eg, drug dose change in response to harms, participant request, or improving/worsening disease)	-
	11c	Strategies to improve adherence to intervention protocols, and any procedures for monitoring adherence (eg, drug tablet return, laboratory tests)	-
	11d	Relevant concomitant care and interventions that are permitted or prohibited during the trial	10
Outcomes	12	Primary, secondary, and other outcomes, including the specific measurement variable (eg, systolic blood pressure), analysis metric (eg, change from baseline, final value, time to event), method of aggregation (eg, median, proportion), and time point for each outcome. Explanation of the clinical relevance of chosen efficacy and harm outcomes is strongly recommended	10-11
Participant timeline	13	Time schedule of enrolment, interventions (including any run-ins and washouts), assessments, and visits for participants. A schematic diagram is highly recommended (see Figure)	15/16

For peer review only - http://bmjopen.bmj.com/site/about/guidelines.xhtml

Sample size	14	Estimated number of participants needed to achieve study objectives and how it was determined, including clinical and statistical assumptions supporting any sample size calculations	6
Recruitment	15	Strategies for achieving adequate participant enrolment to reach target sample size	-
**Methods: Assignment of interventions (for controlled trials)**			
Allocation:			
Sequence generation	16a	Method of generating the allocation sequence (eg, computer-generated random numbers), and list of any factors for stratification. To reduce predictability of a random sequence, details of any planned restriction (eg, blocking) should be provided in a separate document that is unavailable to those who enrol participants or assign interventions	8
Allocation concealment mechanism	16b	Mechanism of implementing the allocation sequence (eg, central telephone; sequentially numbered, opaque, sealed envelopes), describing any steps to conceal the sequence until interventions are assigned	8
Implementation	16c	Who will generate the allocation sequence, who will enrol participants, and who will assign participants to interventions	8
Blinding (masking)	17a	Who will be blinded after assignment to interventions (eg, trial participants, care providers, outcome assessors, data analysts), and how	11
	17b	If blinded, circumstances under which unblinding is permissible, and procedure for revealing a participant's allocated intervention during the trial	N/A
**Methods: Data collection, management, and analysis**			
Data collection methods	18a	Plans for assessment and collection of outcome, baseline, and other trial data, including any related processes to promote data quality (eg, duplicate measurements, training of assessors) and a description of study instruments (eg, questionnaires, laboratory tests) along with their reliability and validity, if known. Reference to where data collection forms can be found, if not in the protocol	10-11
	18b	Plans to promote participant retention and complete follow-up, including list of any outcome data to be collected for participants who discontinue or deviate from intervention protocols	

Data management	19	Plans for data entry, coding, security, and storage, including any related processes to promote data quality (eg, double data entry; range checks for data values). Reference to where details of data management procedures can be found, if not in the protocol	13
Statistical methods	20a	Statistical methods for analysing primary and secondary outcomes. Reference to where other details of the statistical analysis plan can be found, if not in the protocol	12/13
	20b	Methods for any additional analyses (eg, subgroup and adjusted analyses)	N/A
	20c	Definition of analysis population relating to protocol non-adherence (eg, as randomised analysis), and any statistical methods to handle missing data (eg, multiple imputation)	N/A
**Methods: Monitoring**			
Data monitoring	21a	Composition of data monitoring committee (DMC); summary of its role and reporting structure; statement of whether it is independent from the sponsor and competing interests; and reference to where further details about its charter can be found, if not in the protocol. Alternatively, an explanation of why a DMC is not needed	
	21b	Description of any interim analyses and stopping guidelines, including who will have access to these interim results and make the final decision to terminate the trial	N/A
Harms	22	Plans for collecting, assessing, reporting, and managing solicited and spontaneously reported adverse events and other unintended effects of trial interventions or trial conduct	9/10
Auditing	23	Frequency and procedures for auditing trial conduct, if any, and whether the process will be independent from investigators and the sponsor	N/A
**Ethics and dissemination**			
Research ethics approval	24	Plans for seeking research ethics committee/institutional review board (REC/IRB) approval	13
Protocol amendments	25	Plans for communicating important protocol modifications (eg, changes to eligibility criteria, outcomes, analyses) to relevant parties (eg, investigators, REC/IRBs, trial participants, trial registries, journals, regulators)	

For peer review only - http://bmjopen.bmj.com/site/about/guidelines.xhtml

Consent or assent	26a	Who will obtain informed consent or assent from potential trial participants or authorised surrogates, and how (see Item 32)	7
	26b	Additional consent provisions for collection and use of participant data and biological specimens in ancillary studies, if applicable	N/A
Confidentiality	27	How personal information about potential and enrolled participants will be collected, shared, and maintained in order to protect confidentiality before, during, and after the trial	13
Declaration of interests	28	Financial and other competing interests for principal investigators for the overall trial and each study site	14
Access to data	29	Statement of who will have access to the final trial dataset, and disclosure of contractual agreements that limit such access for investigators	-
Ancillary and post-trial care	30	Provisions, if any, for ancillary and post-trial care, and for compensation to those who suffer harm from trial participation	N/A
Dissemination policy	31a	Plans for investigators and sponsor to communicate trial results to participants, healthcare professionals, the public, and other relevant groups (eg, via publication, reporting in results databases, or other data sharing arrangements), including any publication restrictions	13
	31b	Authorship eligibility guidelines and any intended use of professional writers	N/A
	31c	Plans, if any, for granting public access to the full protocol, participant-level dataset, and statistical code	N/A
**Appendices**			
Informed consent materials	32	Model consent form and other related documentation given to participants and authorised surrogates	included
Biological specimens	33	Plans for collection, laboratory evaluation, and storage of biological specimens for genetic or molecular analysis in the current trial and for future use in ancillary studies, if applicable	N/A

*It is strongly recommended that this checklist be read in conjunction with the SPIRIT 2013 Explanation & Elaboration for important clarification on the items. Amendments to the protocol should be tracked and dated. The SPIRIT checklist is copyrighted by the SPIRIT Group under the Creative Commons "Attribution-NonCommercial-NoDerivs 3.0 Unported" license.

For peer review only - http://bmjopen.bmj.com/site/about/guidelines.xhtml

For peer review only

For peer review only - http://bmjopen.bmj.com/site/about/guidelines.xhtml

BMJ Open

One to one befriending for people with intellectual disability and symptoms of depression: A protocol for a pilot randomised controlled trial.

Journal:	*BMJ Open*
Manuscript ID	bmjopen-2019-033989.R2
Article Type:	Protocol
Date Submitted by the Author:	21-Jan-2020
Complete List of Authors:	Ali , Afia; University College London, Division of Psychiatry<br>McKenzie, Emma; North East London NHS Foundation Trust Goodmayes Hospital<br>HASSIOTIS, ANGELA; ROYAL FREEand UNIVERSITY COLLEGE MEDICAL SCHOOL, PSYCHIATRY and BEHAVIOURAL SCIENCES<br>Priebe, Stefan; Barts and the London School of Medicine and Dentistry, University of London, Unit of Social and Community Psychiatry<br>Lloyd-Evans, Brynmor ; UCL, Division of Psychiatry<br>Omar, Rumana; University College London<br>Jones, Rebecca; UCL<br>Panca, Monica; University College London, Primary Care and Population Health<br>Fernandez, Vincent; Outward<br>Finning, Sally; Outward<br>Moore, Shirley; The Befriending Scheme<br>O'Connor, Danielle; The befriending Scheme<br>Roe, Christine; The Befriending Scheme<br>King, Michael; University College Medical School, Division of Psychiatry
**Primary Subject Heading**:	Mental health
Secondary Subject Heading:	Public health
Keywords:	Depression & mood disorders < PSYCHIATRY, QUALITATIVE RESEARCH, Delirium & cognitive disorders < PSYCHIATRY

SCHOLARONE™
Manuscripts

I, the Submitting Author has the right to grant and does grant on behalf of all authors of the Work (as defined in the below author licence), an exclusive licence and/or a non-exclusive licence for contributions from authors who are: i) UK Crown employees; ii) where BMJ has agreed a CC-BY licence shall apply, and/or iii) in accordance with the terms applicable for US Federal Government officers or employees acting as part of their official duties; on a worldwide, perpetual, irrevocable, royalty-free basis to BMJ Publishing Group Ltd ("BMJ") its licensees and where the relevant Journal is co-owned by BMJ to the co-owners of the Journal, to publish the Work in this journal and any other BMJ products and to exploit all rights, as set out in our licence.

The Submitting Author accepts and understands that any supply made under these terms is made by BMJ to the Submitting Author unless you are acting as an employee on behalf of your employer or a postgraduate student of an affiliated institution which is paying any applicable article publishing charge ("APC") for Open Access articles. Where the Submitting Author wishes to make the Work available on an Open Access basis (and intends to pay the relevant APC), the terms of reuse of such Open Access shall be governed by a Creative Commons licence – details of these licences and which Creative Commons licence will apply to this Work are set out in our licence referred to above.

Other than as permitted in any relevant BMJ Author's Self Archiving Policies, I confirm this Work has not been accepted for publication elsewhere, is not being considered for publication elsewhere and does not duplicate material already published. I confirm all authors consent to publication of this Work and authorise the granting of this licence.

For peer review only

One to one befriending for people with intellectual disability and symptoms of depression: A protocol for a pilot randomised controlled trial.

Afia Ali, *[1] Emma McKenzie [2], Angela Hassiotis [1], Stefan Priebe [3], Brynmor Lloyd-Evans [1], Rumana Omar [4], Rebecca Jones [1,5], Monica Panca [5], Vincent Fernandez [6], Sally Finning[6], Shirley Moore [7], Danielle O'Connor [7] Christine Roe [7], Michael King [1]

[1]Division of Psychiatry,

University College of London, UK

afia.ali@ucl.ac.uk

michael.king@ucl.ac.uk

b.lloyd-evans@ucl.ac.uk

a.hassiotis@ucl.ac.uk

reb.jones@ucl.ac.uk

[2] Research and Development Office,

Goodmayes Hospital, North East London NHS Foundation Trust, UK

Emma.mckenzie@nelft.nhs.uk

[3]Unit for Social and Community Psychiatry, (WHO Collaborating Centre for Mental Health Services Development),

Queen Mary University of London, UK

s.priebe@qmul.ac.uk.

[4] Department of Statistical Science

University College London, UK

r.omar@ucl.ac.uk

[5] PRIMENT Clinical Trials Unit,

University College London, UK

reb.jones@ucl.ac.uk

For peer review only - http://bmjopen.bmj.com/site/about/guidelines.xhtml

m.panca@ucl.ac.uk

[6]Outward, Newlon House
4 Daneland Walk, Hale Village, London,
N17 9FE
VFernandez@outward.org.uk
sfinning@outward.org.uk

[7] The Befriending Scheme,
1, The Croft, Sudbury,
Co10 1HN
danielle.oconnor@thebefriendingscheme.org.uk
christine.roe@thebefriendingscheme.org.uk
shirley.moore@thebefriendingscheme.org.uk

* Corresponding author
Afia Ali
Division of Psychiatry,
University College of London,
6th Floor, Maple House,
149 Tottenham court Road,
W1T 7NF
Email: afia.ali@ucl.ac.uk
Telephone: 0207 679 9334

Word count: 4919

For peer review only - http://bmjopen.bmj.com/site/about/guidelines.xhtml

Abstract

Introduction

People with intellectual disability (ID) are more likely to experience loneliness and have smaller social networks, which increases vulnerability to depression. Befriending may reduce depressive symptoms in other populations but randomised controlled trials (RCTs) have not been carried out in this population. This pilot study aims to assess the acceptability and feasibility of carrying out a full RCT of one to one befriending by volunteers for people with ID, compared to an active control group.

Methods and Analysis

The trial aims to recruit 40 participants with ID. Participants in the intervention arm will receive weekly visits from a volunteer over six months. Community befriending schemes will recruit, train, supervise volunteers and match them to individuals with ID. Both groups will receive a booklet about local activities and have access to usual care. Health and social outcomes will be measured at the end of the intervention and 6 months follow up. The following outcomes will be assessed: 1. recruitment and retention of individuals with ID and volunteers in the trial; 2. adverse events related to the intervention; 3. the acceptability of the intervention; 4. whether the intervention is delivered as intended; 5. changes in health and social outcomes and 6. the feasibility of carrying out a cost effectiveness analysis in a full trial. Qualitative data from participants, volunteers, staff and carers will identify barriers and facilitators of a future full trial.

Ethics and Dissemination

The study has been approved by the London- City and East Research Ethics Committee (reference 18/LO/2188). The findings will be presented at conferences and published in a peer reviewed journal and the National Institute of Health Research journals library. A public engagement seminar will be held at the end of the study aimed at key stakeholders.

Trial registration Number: ISRCTN63779614

Strengths and Limitations of the study

- This is the first pilot randomised controlled trial of one to one befriending in people with ID.
- This pilot study will examine the feasibility and acceptability of carrying out a future randomised controlled trial of befriending compared to an active control group.
- Community befriending services will be responsible for all aspects of the delivery of intervention
- Availability of resources within befriending services may influence the delivery of the intervention
- The findings will inform whether a full trial is feasible and what modifications would need to be made to overcome potential barriers.

For peer review only - http://bmjopen.bmj.com/site/about/guidelines.xhtml

Introduction

Intellectual disability (ID) is a life-long condition characterised by impaired cognitive and adaptive functioning, arising before the age of 18 (1). The UK prevalence of ID is 1-2% (2). People with ID have the same or higher prevalence of depression than the general population, but are more likely to experience chronic depression (3-4). They have greater exposure to social disadvantage (5), experience social exclusion because of stigma (6), have markedly smaller social networks (7-8) and a higher prevalence of loneliness compared to the general population (9). These factors have been associated with depressive symptoms in this group (10-13).

Conceptualisation of befriending

Befriending is "a relationship between two or more individuals, initiated, supported and monitored by an agency. The relationship is non-judgmental, mutual, purposeful, and there is a commitment over time" (14). Key attributes are that it is a one to one friendship-like relationship, it is an organised intervention, and that there is a negotiation of power (15). There is a wide variation in the concept and practice of befriending (16). At one extreme, befriending is very close to a friendship, characterised as being reciprocal and is delivered by lay volunteers, and at the other end it is a professional and therapeutic relationship, focused on the befriendee attaining goals and aspirations (mentoring). Most types of befriending relationships lie midway on this spectrum. Schemes are usually run by voluntary organisations in the community and offer training, supervision and on-going support to volunteers (16).

Befriending aims to help people who are lonely, isolated and have limited opportunities for social participation by increasing social and emotional support and by enhancing social networks and community participation. The causal mechanisms of befriending are unclear but social support is thought to be important. Social support may act as a buffer to stress and it may mediate genetic and environmental vulnerabilities to depression through its effects on neurobiological factors and other psychosocial factors (e.g. coping strategies) (17)The Befriender may enhance social support and link the befriendee into social activities, which may be sustainable outside of, and beyond the end of the befriending relationship, which may result in longer term benefits. Befriending may also improve health outcomes thorough its effects on social networks (18).

There are also potential benefits for befrienders. Motivation for befriending often includes a desire for "giving" something back to the community (e.g. helping others) and "getting" something in return (e.g. acquiring new skills or meeting new people) (19, 20)). Positive benefits reported by befrienders include increased self-esteem and confidence, feeling that they have gained a genuine friend themselves and improved attitudes towards people with mental illness (19). Volunteering in general has beneficial effects on depression, psychological wellbeing and life satisfaction, and is associated with lower risk of mortality, although the causal mechanisms for these associations are unclear (21).

However, the benefits of befriending may be short-lived and people with ID have reported feeling distressed following the termination of their befriending relationship (22). Other risks include the emotional turbulence that is associated with a natural friendship, or harmful

For peer review only - http://bmjopen.bmj.com/site/about/guidelines.xhtml

effects if the befriender is not adequately trained or supervised. There may also be undue burden placed on the befriender to take on excessive responsibility (16).

Effectiveness of befriending

One meta-analysis found that befriending in people with mental or physical health problems, (delivered by social and health care professionals as well as lay volunteers) had a statistically significant but modest effect on reducing symptoms of depression when compared to no treatment or treatment as usual in both the short and long term (23). Another systematic review examined a range of health and social outcomes in studies where befriending was delivered by volunteers only (24). Befriending was associated with better patient reported outcomes when all primary outcomes were combined but the effect size was small (24). However, in contrast to the previous review, there was limited evidence for the effectiveness of befriending on individual outcomes such as depression, loneliness or quality of life when the studies were combined (24). A recently published randomised controlled trial (RCT) of befriending by volunteers in people with psychosis provides further evidence for the potential beneficial effects of befriending (25). Participants in the intervention arm had significantly more social contacts at the end of the 12 month intervention and at six months follow up, suggesting that befriending may help to reduce social isolation in this is group.

The effectiveness of befriending in people with ID has not been evaluated in a randomised trial. An unpublished single arm feasibility study of one to one befriending by volunteers, conducted by a voluntary organisation, (26) recruited 24 volunteers, of which 15 were matched with an individual with ID. Positive change was reported in 60% of individuals with ID; 53% reported a decrease in isolation and 40% reported an increase in confidence. One Australian study examined the feasibility of using "active mentoring", whereby members of existing community groups were trained to act as mentors to older adults with ID, in order to provide social support and to encourage participation in community groups (27). The intervention comprised 29 individuals receiving the intervention and a matched comparison group. The participants in the intervention reported better social satisfaction compared to the comparison group but there were no significant changes in the other outcomes.

Given the dearth of studies examining befriending in people with ID there is a clear rationale for carrying out a pilot study prior to a full randomised controlled trial.

Aims and Objectives

The main aim of the study is to determine the feasibility and acceptability of a full scale randomised controlled trial of one to one befriending by volunteers for people with ID in addition to usual care, compared to an active control arm.

The objectives are to:

- examine the recruitment and retention of individuals with ID and volunteers in the trial and the number of successfully matched pairs within the six month study recruitment period
- Record any negative consequences/adverse effects of befriending
- Measure the extent to which the intervention is delivered as intended by volunteers and the befriending schemes

For peer review only - http://bmjopen.bmj.com/site/about/guidelines.xhtml

- Examine the acceptability of the intervention and study procedures by exploring the views of individuals, volunteers, carers and befriending services.
- Examine changes in health and social outcomes by carrying out exploratory analyses of the impact of befriending on depressive symptoms measured by the Glasgow Depression Scale (GDS (28)) at 12 months and other outcomes (psychological distress, self-esteem, loneliness, quality of life, and social participation) at 6 and 12 months post randomisation.
- Carry out exploratory analyses of the impact of befriending on volunteers' wellbeing, self-esteem, loneliness and attitudes towards people with intellectual disability at 6 and 12 months.
- Estimate the sample size required and determine the final trial design for a full scale randomised controlled trial.
- Assess the feasibility of collecting data that would inform a future analysis of cost effectiveness.

Methods

Design

This is a two arm, parallel group, researcher blind pilot RCT with 1:1 allocation. Fifty participants with ID who are eligible for the study will be randomly allocated to either the intervention arm (one to one befriending by a volunteer) or an active control arm. Both groups will have access to usual care and a booklet of local resources. The duration of the intervention will be 6 months. Outcome assessments will be carried out at baseline, post intervention and at six months follow-up. A process evaluation, based on mixed methods, will be carried out to examine the delivery and adherence to intervention, and stakeholder views on the acceptability of the intervention and barriers and facilitators that may affect the implementation of a full scale trial.

Sample Size

We do not have any estimates of the number of people with ID who are eligible and are likely to consent to taking part in the trial. If we approach 50 participants who are eligible to take part, this will allow us to estimate an expected recruitment rate of 80% (40 people), with a 95% confidence interval (CI) of 68.9% to 91.1%. A sample size of 40 recruited people with ID would allow us to estimate a 30% drop out rate in the trial with a 95% CI of 25.7% to 54.3%. The recruitment period is 6 months. There are two participating befriending services and therefore we will need to recruit 3.3 participants with ID per month at each site.

Participants

Inclusion Criteria

We will include: individuals with ID aged 18 or over, who have mild or moderate intellectual disability (IQ of 35-60) assessed using the Wechsler Abbreviated Scale of Intelligence (WASI-II; (29)); should not be attending college/education or a day service for three or more days a week; will have a score of 5 or more on the Glasgow Depression Scale for People with Learning Disability (GDS-LD (28)), indicating the presence of depressive symptoms but

For peer review only - http://bmjopen.bmj.com/site/about/guidelines.xhtml

do not need to have a clinical diagnosis of depression. Participants will need to be able to speak English and provide informed consent.

Volunteers aged 18 or over, who can agree to being available once a week for at least one hour over a period of six months. Volunteers do not need to have any prior experience of supporting people with ID.

Exclusion Criteria

Individuals with ID will be excluded if they have limited communication and comprehension and therefore would not be able to complete the questionnaires.

Volunteers will be excluded if they have a criminal record (any documented offence due to the vulnerability of this population) recorded on their Disclosure and Barring Service (DBS) check or are unable to provide two references or have unsuitable references.

Recruitment

Befriending services

Two befriending services ("Outward", based in Hackney, North London and "the befriending scheme" in Suffolk) will be taking part in the study and will be responsible for overseeing the befriending intervention. Both services have experience supporting befriending relationships with people with ID including matching individuals with volunteers and monitoring relationships.

Participants with ID

Participants with ID will be recruited from existing and new referrals to the befriending services or recruited from the caseload of clinicians working in community learning disability services in North London and Suffolk. Volunteer coordinators and clinicians will screen referrals and caseloads for participants who may be eligible for the study and will make the initial approach. If the individual is interested in the study, and consents to his/her details being passed on to the research team, a referral form will be completed and emailed to the research team. The research assistant will then contact the individual and carry out an eligibility assessment. If the individual is eligible, they will need to provide written consent before completing the baseline assessment.

Volunteers

The befriending services will advertise and recruit volunteers through newspaper advertisements, befriending and job websites, social media and recruitment events at colleges and universities. Volunteers will be recruited directly from the study website hosted by the UCL Division of Psychiatry and through the Division's twitter account. Interested volunteers will complete an application form and will be invited to an informal interview to assess their suitability and motivation for taking part in the scheme. A DBS check will be completed to ensure that they have no criminal records and references will be obtained. People with ID are potentially a vulnerable group and therefore volunteers with any previous offence, including minor offences, will not be included in the study. Successful candidates

For peer review only - http://bmjopen.bmj.com/site/about/guidelines.xhtml

will be invited to take part in the study. They will receive an information sheet and asked to sign a consent form, followed by completion of the baseline assessment.

Randomisation

Randomisation will be carried out after the baseline assessments have taken place. Participants will be randomised into the study by an unblinded member of the research team who will enter the patients' details into a web based randomisation system, which is hosted on a secure server by Sealed Envelope. This system will randomly allocate the participant to either the intervention or control arm. The unblinded researcher will notify the befriending service and participants with ID of their allocation.

Randomisation will be blocked using randomly varying block sizes, stratified by centre. The allocation schedule will be concealed through the use of this central web-based randomisation service. The randomisation protocol will be created by the trial statistician and the set up of the service will be overseen by the Priment Clinical Trials Unit.

Intervention group

Participants with ID will meet with the volunteering coordinator to obtain information about their hobbies, interests and activities they would like support to participate in. Based on this information, participants with ID will be matched to a volunteer that can accommodate the person's interests.

i. The befriending intervention

The befriending intervention has been adapted from the existing models used by the two participating befriending services and from other studies of befriending (26, 30). The purpose of the befriending relationship will be to provide friendship and emotional support, and to assist the individual to access activities in the community that they may be unable to do alone. As participants become more confident, they will be encouraged to access activities in the community on their own, in order to promote sustained social activities beyond the befriending relationship.

The volunteer (befriender) and person with ID will meet once a week for at least one hour, for six months, although some breaks are anticipated due to holidays or illness. They will receive a booklet detailing local activities and amenities, which they can use to plan activities. The emphasis will be on assisting the individual to make choices about the activities that they wish to pursue. The volunteer is not expected to carry out personal care, administer medication or accompany the individual to medical appointments. Contacts by phone/social media can take place in addition to face to face contacts. The pair can spend some sessions in the person's home but this should not exceed 50% of the total number of sessions. Sessions may take place during evenings/weekends depending on the pair's availability. They will keep a record of their activities in a structured log that will be provided (whether they attended each session, reasons for cancellation, what they did in each session and duration of activity), and record of other types of contact. Volunteers will be reimbursed travel expenses but other expenses will need to be agreed with the befriending service. Participants with ID will not be reimbursed travel expenses or the costs of activities.

ii. Introduction and monitoring of the befriending relationship

For peer review only - http://bmjopen.bmj.com/site/about/guidelines.xhtml

The volunteering coordinator will arrange a face-to-face meeting where the pair will be introduced to each other. If they agree to continue, the pair will arrange to meet on their own; if they decide that the pairing is unsuitable, they will be re-matched. If the volunteer or individual with ID drops out of the relationship once it has become established, attempts will be made to re-match them. Volunteers may be matched to more than one participant with ID.

The volunteering coordinator will arrange a face-to-face meeting with the pair after six weeks and will maintain contact with each person by telephone/ face to face contact every four weeks thereafter to monitor the progress of the relationship. A further meeting will be held with the pair at the end of the six months to obtain general feedback about the befriending intervention, to discuss ending the relationship and to support the individual with ID with coming to terms with the ending. The pair may continue their relationship if they wish after the six month period but arrangements for monitoring the relationship will vary depending on the befriending service. Information will be obtained on any relationships continuing beyond six months and the monitoring that has been provided.

iii. Training and supervision of volunteers

The volunteers will attend training delivered face-to-face and as e-learning. The training will cover: the benefits of befriending and issues related to confidentiality and lone working; advice on how to plan meetings effectively; health and safety; safeguarding ; learning disability awareness, and professional boundaries, which covers dealing with sensitive issues, ending relationships and expectations of the role of the volunteer. Volunteers will also receive slides and a manual developed by the research team, with information about the study and additional information about communication, challenging behaviour and mental health problems.

Volunteers will have access to group or individual supervision delivered face to face or over the phone, once a month, by the volunteer coordinator, which will address issues that may have arisen from the relationship, for example, concerns about mental health or behaviour.

iv. Training and support for befriending services

Participating befriending services will complete Good Clinical Practice (GCP) Training and undergo training on the study research processes and procedures as part of the site initiation visit. Volunteer coordinators will receive support from the research team through regular email and telephone contact and they will attend Trial Management Group (TMG) meetings.

Control Group

Participants in the control arm will receive the activities booklet. They will meet with a member of the research team who will discuss the booklet with them (and their carer, if present) and encourage them to engage with activities. This is to control for the effects of participants in the treatment arm receiving more information about local activities.

For peer review only - http://bmjopen.bmj.com/site/about/guidelines.xhtml

For peer review only

Both the control and intervention arms will also have access to "usual care". This will include access to multidisciplinary input from community intellectual disability services. Participants can continue to take their usual medication and can access other community and hospital health services and day services. Information about usual care will be collected as part of the baseline assessment.

Outcomes

i. Recruitment and retention of participants:
We will examine the proportion of participants with ID recruited from among those eligible; and the proportion of volunteers recruited from among people expressing an interest over a six months period; the proportion of participants with ID who are successfully matched with a volunteer; the proportion of participants with ID and volunteers who drop out of the intervention arm and the proportion of participants and volunteers who complete subsequent follow up assessments.

ii. Adverse events
Adverse events will be collected at each follow up assessment using open-ended questions and will also be reported directly to the Chief Investigator by the befriending services, including concerns about safeguarding. Volunteers will follow a protocol if they have concerns about the participant's mental health, which will involve informing the volunteer coordinator, who will in turn notify the CI and the referring clinician or a health professional involved in the person's care. If a serious incident (e.g. self -harm, challenging behaviour) occurs out of hours, the carer will be notified and emergency services will be contacted if appropriate. All adverse events will be recorded in the medical records and CRFs. Serious adverse events (e.g. safeguarding concerns, hospitalisation) will be recorded in the Serious Adverse Effects (SAE) log.

iii. Acceptability of the intervention
This will be informed by data on retention/ drop out of volunteers and participants, the extent of engagement with the intervention by participants and volunteers (based on number of sessions attended) and qualitative data obtained from volunteers, participants with ID, carers of people with ID and staff from the befriending service.

i*v. Adherence to the intervention*
Data will be collected on volunteer training, uptake of supervision and the frequency of monitoring checks carried out by volunteer coordinators from routine records at each site in order to assess fidelity to the intervention by the befriending services. Structured logs provided by volunteers will be analysed to assess fidelity to the intervention by volunteers. We will examine: a) how many sessions were attended by each volunteer, reasons for non-attendance, and how many attended at least 10 sessions during the 6-month intervention period; and b) for how many participants the minimum threshold of at least 50% of meetings being outside the participant's home was achieved.

v. Depression and other health and social outcome measures in participants with ID

For peer review only - http://bmjopen.bmj.com/site/about/guidelines.xhtml

All the health and social outcome measures have been validated in people with ID and will be assessed with the participant at baseline, at the end of the intervention and at six months follow up. (see table 1: schedule of assessments). Assessments will be carried out by a research assistant (RA) blind to group allocation and will take place face to face at the befriending service or the participants' homes.

- Depressive symptoms will be measure using the GDS-LD (28). Scores at 12 months will be the main health outcome of interest.
- Self-esteem will be measured using the adapted Rosenberg self-esteem scale for people with intellectual disabilities (31)
- Quality of life will be measured using the Maslow Assessment of Needs Scale-Learning Disability (MANS-LD; (32)) and five items from the adapted World Health Organisation Quality of Life questionnaire (WHO-QOL-8; (33)).
- Loneliness and social satisfaction will be measured using the Modified Worker Loneliness Questionnaire (MWLQ (34),
- Social support will be measured using the Social Support Self Report for intellectually disabled adults (SSSR) (35)
- Social participation will be measured at using the Guernsey Community Participation and Leisure Assessment (GCPLA) (36).

vi. Volunteer outcome measures

The following outcome assessments will be carried out in volunteers at baseline (prior to matching) and post intervention (six months after the baseline) and at six months follow-up (see table 2 schedule of assessments):

- Self-esteem will be measured using the 10 item Rosenberg self-esteem scale (37)
- Psychological wellbeing and quality of life will be measured using the Warwick-Edinburgh mental wellbeing scale (WEMWBS; 38).
- Loneliness will be measured using the UCLA loneliness scale (39).
- Attitudes of volunteers will be assessed using the 67 item, Attitudes Towards Intellectual Disability Questionnaire (ATTID; 40)

vii. Feasibility of carrying out a cost effectiveness analysis

The preliminary health economic analysis will inform planning of future economic analyses, sources of data required and how best to collect these data. The following measures will be recorded at baseline, post intervention (six months after randomisation) and at six months follow-up.

- Health related quality of life will be measured using the EuroQol-Youth (EQ-5D-Y) (41). We will assess the feasibility of using the EQ-5D-Y to calculate Quality adjusted life years (QALYs). QALYs will be calculated using the EQ-5D-3 L tariff, as there are no value sets for the EQ-5 D-Y (42).
- Client Services Receipt Inventory (CSRI) for people with intellectual disabilities adapted for the study (43), completed with carers where possible, will assess the feasibility of collecting participant reported service use over the previous six months.

For peer review only - http://bmjopen.bmj.com/site/about/guidelines.xhtml

Process evaluation

A process evaluation will be undertaken, based on MRC guidance (44). The aim will be to examine whether the different components of the intervention were consistently followed; the extent to which volunteers delivered the intervention as intended; to understand the perceived value, benefits and unintended consequences of the intervention so that these are fully measured in a full trial, contextual factors influencing how the intervention was delivered; and to develop an understanding of the likely mechanisms of action of the intervention. We will interview 16 participants with ID after their 12 month follow up (8 per site) and carry out two focus groups at each site with 5-8 volunteers, staff from each befriending service, and 5-8 carers. We will use purposive sampling in order to include people with a range of demographic characteristics (e.g. gender, age, ethnicity, living arrangements), and both participants and volunteers for whom the befriending relationship broke down, as well as people who completed the intervention. All respondents will be asked about what aspects of the intervention were perceived to be helpful, what aspects require modification and suggestions for improvement, views about trial procedures and the perceived barriers and facilitators to delivering a larger trial. The interviews and focus groups will be audio-taped and transcribed.

In order to understand how the befriending intervention was delivered, the location, content of the meetings and the range of activities undertaken will be described based on an analysis of structured log books. A framework will be developed to categorise different types of activity to enable different types of befriending support to be distinguished and quantified. Test procedures will be developed for collecting quantitative process data, which, in a future larger trial, could be used to explore the relationships between process variables and outcomes. Findings from the process evaluation will inform any necessary refinements to the study intervention or procedures.

Analysis

Feasibility outcomes such as the number of participants who were screened and eligible, the proportion of eligible participants with ID and interested volunteers recruited to the study, and the proportion successfully matched in befriending relationships, will be reported. The number (proportion) of drop-outs of volunteers and participants from the intervention arm and from both arms at each follow up assessment, will also be reported, including reasons why where possible. This information will be presented in a Consort diagram describing the flow of participants through the study. (http://www.consort-statement.org/).

The characteristics and outcomes of participants by trial arm will be summarised using means and standard deviations or medians and inter-quartile ranges for continuous variables and count and percentages for categorical variables. Appropriate regression models depending on the type of outcome, adjusted for baseline values and centre will be used to estimate the intervention effect on health and social outcomes where possible. The results will be presented as estimates with 95% CI. All analyses will be carried out as randomised with available data (intention to treat principle) and will be used to inform the definitive trial. The characteristics of patients with missing outcome data will be investigated.

For peer review only - http://bmjopen.bmj.com/site/about/guidelines.xhtml

Economic Evaluation

We will assess the feasibility of gathering information for a cost-effectiveness analysis for a full RCT including testing the suitability of calculating quality-adjusted life years (QALYs) using the EQ-5D-Y. We will calculate the costs of delivering the befriending intervention (training, supervision and expenses), which will be obtained from each participating befriending service. Mean resource use/ costs (standard deviation (SD)) per participant at baseline and follow-up, based on completion of the CSRI, will be presented.

Qualitative analysis

Transcripts from the focus groups and interviews will be analysed by the study team using thematic analysis supported by computer software (NVivo 9). The analytic strategy will focus on identifying barriers and facilitators to implementing the intervention successfully, its benefits to participants and mechanisms of effect. Analysis will also allow consideration of themes that arise more inductively from the data. Validity will be enhanced by a collaborative analytic strategy involving members of the research team and the advisory group who will meet together to review the coding framework and agree on the themes.

Patient and Public Involvement

Volunteers, people with ID and befriending schemes were involved in the design of the study. During the study, there will be advisory groups comprising carers, current volunteers and individuals with ID who will provide advice about the study information sheets, consent forms, topic guides for the qualitative interviews and focus groups, results of the study findings and the final study report. Members will be invited to be part of the TMG and will be involved in carrying out the qualitative interviews and focus groups as part of the process evaluation. They will receive training and support for this role. We will also invite members to participate in the public engagement seminar at the end of the study.

Ethics, governance and dissemination

Amendments to the study protocol and documents will be approved by the sponsor (UCL) and the Ethics Committee. Priment Clinical Trials Unit (CTU) will ensure that the trial procedures meet the requirements of Good Clinical Practice (GCP) and will complete dataquality assurance checks. The RA will have experience working with people with ID and will receive training on assessing capacity and carrying out assessments. Supervision of the RA will be provided by the Chief Investigator (AA). The study team, co-applicants, PPI advisors and the CTU will be part of the TMG and will meet every 10-12 weeks to discuss the progress of the study. An independent trial steering committee will provide overall supervision of the trial and will report to the funders. Data confidentiality will be maintained by assigning participants study identification numbers and data will be entered anonymously and stored in a secure web-based database.

The findings of the study will be presented at conferences and published in an open access peer reviewed journal as well as the National Institute of Health Research (NIHR) Public Health Research Journal. A public engagement seminar targeted at relevant stakeholders will take place at the end of the study to discuss the study findings and implications. A summary report will be developed for participants and volunteers taking part in the study and will be published on the study website.

For peer review only - http://bmjopen.bmj.com/site/about/guidelines.xhtml

Study timeline

Recruitment of participants began in April 2019. There will be a six month recruitment period, a further 12 months to complete the follow up assessment and three months to complete the process evaluation, analyse the results and write up the study findings.

Discussion

This is the first pilot RCT of one to one befriending, monitored by community befriending services, in people with ID. The trial will provide data on whether a full trial will be feasible, in terms of recruitment and retention of volunteers and people with ID and data on potential beneficial and adverse effects, acceptability and the extent to which the intervention is delivered as intended by volunteers and the befriending services. It will help to inform modifications that need to be made to enable a future trial to overcome barriers and challenges that may be encountered in this pilot.

There is currently limited evidence supporting the choice of a primary health outcome but the use of depressive symptoms is supported by one systematic review (24). This pilot study will help to determine whether measuring depressive symptoms is the most appropriate primary outcome for a future trial.

One of the main challenges of this study will be to ensure that the intervention and the recruitment, training and supervision of volunteers are carried out according to the trial protocol as these aspects vary between befriending services, and was an issue in a recent trial of befriending in people with psychosis (25) While both befriending services have committed to the study, there are resource implications for these services as the intervention costs are not covered by the research funding. External factors such as funding cuts to these services could influence the delivery of the intervention and the success of the trial. The process data will allow us to examine whether a future trial would need to be more pragmatic and whether the befriending intervention should permit more flexibility.

Author Contributions: AA is the chief investigator and is responsible for the overall study design and conduct. AA, SP, MK, AH, BL-E and RO have contributed to the design of the study and methodology. SF, VF, DO, CR, SM are responsible for designing and delivering the intervention. EM will carry out the data collection. RO, RJ and MP have contributed to the analysis plan. All the authors have reviewed and approved the manuscript.

Funding statement: The study/ project is funded by the National Institute of Health Research (NIHR) (Public Health Research Funding Stream (project reference: 16/122/57). The views expressed are the views of the authors and are not necessarily those of the NIHR of the Department of Health and Social Care.

Competing Interests statement: There are no conflicting interests to declare.

For peer review only - http://bmjopen.bmj.com/site/about/guidelines.xhtml

Table 1: Schedule of assessments in participants with ID

Assessment/Outcome	Screening and baseline assessment	Post intervention assessment	6 months follow up
Eligibility confirmation:<br>WASI-II<br>GDS-LD	x		
Informed consent	x		
Socio-demographic questionnaire	x		
Adapted Rosenberg self-esteem scale	x	x	x
MANS-LD	x	x	x
WHO-QOL-8	x	x	x
MWLQ	x	x	x
SSSR	x	x	x
GCPLA	x	x	x
EQ-5D-Y	x	x	x
CSRI	x	x	x
Adverse effects review	x	x	x
Concomitant medication	x	x	x
Semi-structured interview (optional)			x

WASI-II (Wechsler Abbreviated Scale of Intelligence, 2nd edition); GDS-LD (Glasgow Depression Scale for People with Learning Disability); MANS-LD (Maslow Assessment of Needs Scale learning Disability); WHO-QOL-8 (World Health Organisation Quality of Life questionnaire); MWLQ (Modified Worker Loneliness Questionnaire; SSSR (Social Support Self Report for intellectually disabled adults); GCPLA (Guernsey Community Participation and Leisure Assessment); EQ-5D-Y (EuroQol-Youth); CSRI (Client Services Receipt Interview).

For peer review only

For peer review only - http://bmjopen.bmj.com/site/about/guidelines.xhtml

Table 2: Schedule of assessments with volunteers

Assessment/ outcome	Screening and baseline	Post intervention assessment	6 months follow up
Eligibility check:<br>Informal interview;<br>DBS check<br>references	x		
Informed consent	x		
Rosenberg self-esteem scale	x	x	x
WEMWBS	x	x	x
UCLA Loneliness Scale	x	x	x
ATTID	x	x	x
Focus group (optional)			x

WEMWBS Warwick- Edinburgh mental wellbeing scale; ATTID (Attitudes Towards Intellectual Disability Questionnaire)

For peer review only

References

1. *American Psychiatric Association. Diagnostic and Statistical Manual of Mental Disorders (5th ed.).* Arlington, VA: American psychiatric Publishing; 2013.

2. Emerson E, Hatton C, Robertson J, Roberts H, Baines S, Glover Gyles. *People with learning disabilities in England 2010: services and supports.* Improving Health and Lives Learning Disability Observatory, 2010 (accessed from. http://www.improvinghealthandlives.org.uk/uploads/doc/vid_9244_IHAL2011-02PWLD2010.pdf

3. Cooper S-A, Smiley E, Morrison J, Williamson A, Allan L. An epidemiological investigation of affective disorders with a population-based cohort of 1,023 adults with intellectual disabilities. *Psychological Medicine*, 2007;37:873–82.

4. Richards M, Maughan B, Hardy R, et al. Long-term affective disorder in people with mild learning disability. *British Journal of Psychiatry*, 2001;179:523-7.

5. Emerson E and Einfeld SL. *Challenging Behaviour*. Cambridge: Cambridge University Press; 2011

6. Ali A, Hassiots A, Strydom A, King M. Self stigma in people with intellectual disabilities and courtesy stigma in family carers: A systematic review. *Research in Developmental Disabilities*, 2012; 33: 2122-2140

7. Verdonschot M.M.L,deWitte L.P, Reichrath E, Buntinx W.H.E, &Curfs L.N.G. Impact of environmental factors on community participation of persons with an intellectual disability: A systematic review. *Journal of Intellectual Disability Research*, 2009; 53(1), 54–64.

8. Hill RA & Dunbar RIM. Social network size in humans. *Human Nature*, 2003; 14(1):53-72.

9. Petroutsou A, Ali A, Hassiotis A. Loneliness in people with intellectual and developmental disorders (IDD) across the life span: a systematic review of prevalence and interventions. *Journal of Applied Research in Intellectual Disability* (published online Feb 2018).

10. Ali A, King M, Strydom S, Hassiotis A. Self-reported stigma and symptoms of anxiety and depression in people with intellectual disabilities: Findings from a cross sectional study in England. *Journal of Affective Disorders*, 2015, 187:224-231

11. Emerson E. Self reported exposure to disablism is associated with poor self reported health and wellbeing among adults with intellectual disabilities in England: a cross sectional survey. *Public Health*, 2010; 124, 682-689

12. Ailey S H, Miller A M, Heller T, & Smith E V. Evaluating an interpersonal model of depression among adults with Down syndrome. *Research and Theory for Nursing Practice*,

For peer review only - http://bmjopen.bmj.com/site/about/guidelines.xhtml

2006; *20*(3), 229-246.

13. Merrick, J., Merrick, E., Lunsky, Y., & Kandel, I. A review of suicidality in persons with intellectual disability. *The Israel Journal of Psychiatry and Related Sciences*, 2006; *43*(4), 258-264.

14. Dean, J. & Goodlad, R.*Supporting Community Participation: the role and impact of befriending.* Brighton: Pavilion Publishing & Joseph Rowntree Foundation, 1998.

15. Balaam MC. A concept analysis of befriending. J Adv Nurs, 2015, 71(1): 24-34.

16. Thompson R, Valenti E, Siette J, Priebe S. To befriend or to be a friend: a systematic review of the meaning and practice of “befriending” in mental health care. *J Men Health*, 2016, 25(1):71-77.

17. Ozbay F, Johnson DC, Dimoulas E, Charney D, and Southwick S. Social Support and Resilience to Stress. From Neurobiology to Clinical Practice. *Psychiatry (Edgmont),* 2007; 4(5): 35–40.

18. Berkman L, Glass T, Brissette I, Seeman T. From social integration to health: Durkheim in the new millennium. *Social Science and Medicine*, 2000; 51; 6:843-57

19. Klug G, Klug G, Toner S, Fabisch K, Priebe S. Characteristics and motivations of volunteers providing one-to-one support for people with mental illness: a survey in Austria. *Social Psychiatry and Psychiatric Epidemiology*. 2018; 53:841-847

20 Toner S, Hickling LM, Pinto da Costa M, Cassidy M, Priebe S. Characteristics, motivations and experiences of volunteer befrienders for people with mental illness: a systematic review and narrative synthesis. *BMC Psychiatry*. 2018;18:378

21. Jenkinson CE, Dickens AP, Jones K, Thompson-Coon J, Taylor RS, Rogers M, Bambra CL, Lang I, Richards SH. Is volunteering a public health intervention? A systematic review and meta-analysis of the health and survival of volunteers. *BMC Public Health*. 2013; 13:773.

22. Heslop P. Good practice in befriending services for people with learning disability. *British Journal of Learning Disabilities*, 2005; 33:27-33.

23. Mead N, Lester H, Chew-Graham C, Gask L, Bower P et al. Effects of befriending on depressive symptoms and distress: systematic review and meta-analysis. *The British Journal of Psychiatry,* 2010, 196: 96-101.

24. Siette J, Cassidy M, Priebe S. Effectiveness of befriending interventions: a systematic review and meta-analysis. *BMJ Open*, 2017, 7:e014304.

For peer review only - http://bmjopen.bmj.com/site/about/guidelines.xhtml

25. Priebe S[1], Chevalier A[2], Hamborg T[3], Golden E[4], King M[5], Pistrang N[6]. Effectiveness of a volunteer befriending programme for patients with schizophrenia: randomised controlled trial. ;British Journal of Psychiatry. 2019; 4:1-7

26. Florides T. *Running a befriending project for people with learning disabilities. Final project report.* Circle Support; 2012 (accessed from http://www.mandbf.org/wp-content/uploads/2012/05/Running-a-Befriending-Project-for-Adults-with-Learning-Disabilites-The-Final-Report.pdf)

27. Stancliffe R J., Bigby C, Balandin S, Wilson N J, Craig D. Transition to retirement and participation in mainstream community groups using active mentoring: a feasibility and outcomes evaluation with a matched comparison group. *Journal of Intellectual Disability Research, 2015; 59*(8), 703–718.

28. Cuthill FM, Espie CA, Cooper SA. Development and psychometric properties of the Glasgow Depression Scale for people with a Learning Disability. Individual and carer supplement versions. *British Journal of Psychiatry*, 2003;182:347–53.

29. Wechsler D. Wechsler Abbreviated Scale of Intelligence, Second Edition (WASI-II). San Antonio, TX: NCS Pearson; 2011.

30. Priebe S, Pavlickova H, Eldridge S, Golden E, McCrone P, Ockenden N, Pistrang N, King M. Effectiveness of one-to-one volunteer support for patients with psychosis: protocol of a randomised controlled trial. *BMJ Open*, 2016, 6:e011582.

31. Dagnan D, Sandhu S. Social comparison, self esteem and depression in people with intellectual disability. *Journal of Intellectual Disability Research*, 1999: 372-379.

32. Skirrow P, Perry E. The Maslow Assessment of Needs Scales, 2009; Mersey Care NHS Trist, Liverpool.

33. World Health Organisation Quality of Life Assessment group. Development and general psychometric properties. Social Science and Medicine, 1998; 46: 1569-1585

34. Stancliffe RJ, Keane S. Outcomes and costs of community living: a matched comparison of group homes and semi-independent living. *Journal of Intellectual & developmental Disability,* 2000; 25: 281-305.

35. Lunsky Y, Benson B A, Reliability of ratings of consumers with mental retardation and their staff on multiple measures of social support. American Journal on Mental Retardation *1997;* 102, 280–4.

36. Baker PA. Measurement of community participation and use of leisure by service users with intellectual disabilities: The Guernsey community participation and leisure

For peer review only - http://bmjopen.bmj.com/site/about/guidelines.xhtml

assessment (GCPLA). *Journal of Applied Research in Intellectual Disabilities*, 2000; 13:169-185

37. Rosenberg M. *Society and the adolescent self-image*. Princeton, NJ: Princeton University Press; 1965

38. Tennant R, Hiller L, Fishwick R, Platt S, Joseph S, Weich S et al. The Warwick-Edinburgh mental well-being scale (WEMWBS): development and UK validation. *Health and Quality of Life Outcomes*, 2007, 5(1): 63.

39. Russell, D., Peplau, L.A., & Cutrona, C.E. (1980). The revised UCLA Loneliness Scale:Concurrent and discriminant validity evidence. *Journal of Personality and Social Psychology,* 39, 472-480.

40. Morin D, Crocker AG, Beaulieu-Bergeron R, Caron J. Validation of the attitudes toward intellectual disability: ATTID questionnaire. *Journal of Intellectual Disability Research,* 2013; 57(3):268-78

41. Wille N, Badia X, Bonsel G, Burstrom K, Cavrini G, Devlin N, Egmar AC, Greiner W, Gusi N, Herdman M, Jelsma J, Kind P, Scalone L, Ravens-Sieberer U. Development of the EQ-5D-Y: a child-friendly version of the EQ-5D. *Quality of Life Research*, 2010; 19:875–886. https://euroqol.org/eq-5d-instruments/eq-5d-y-about/

42. EQ-5D-Y (Youth) About. https://euroqol.org/eq-5d-instruments/eq-5d-y-about/. Accessed November 2019.

43 Beecham J, Knapp M. Costing psychiatric interventions. In Measuring Mental Health Needs 2nd ed (ed G Thornicroft): 200–24. Gaskell, 2001

44. Moore GF, Audrey S, Barker M, Bond L, Bonell C, Hardeman W, Moore L, O'Cathain A, Tinati T, Wight D, Baird J. Process Evaluation of complex interventions: Medical Research Council Guidance. *British Medical Journal*, 2015; 350:h1258

For peer review only

STANDARD PROTOCOL ITEMS: RECOMMENDATIONS FOR INTERVENTIONAL TRIALS

SPIRIT 2013 Checklist: Recommended items to address in a clinical trial protocol and related documents*

Section/item	Item No	Description	Addressed on page number
**Administrative information**			
Title	1	Descriptive title identifying the study design, population, interventions, and, if applicable, trial acronym	1
Trial registration	2a	Trial identifier and registry name. If not yet registered, name of intended registry	2
	2b	All items from the World Health Organization Trial Registration Data Set	
Protocol version	3	Date and version identifier	N/A
Funding	4	Sources and types of financial, material, and other support	14
Roles and responsibilities	5a	Names, affiliations, and roles of protocol contributors	1, 13/14
	5b	Name and contact information for the trial sponsor	13
	5c	Role of study sponsor and funders, if any, in study design; collection, management, analysis, and interpretation of data; writing of the report; and the decision to submit the report for publication, including whether they will have ultimate authority over any of these activities	14
	5d	Composition, roles, and responsibilities of the coordinating centre, steering committee, endpoint adjudication committee, data management team, and other individuals or groups overseeing the trial, if applicable (see Item 21a for data monitoring committee)	13

For peer review only

For peer review only - http://bmjopen.bmj.com/site/about/guidelines.xhtml

**Introduction**			
Background and rationale	6a	Description of research question and justification for undertaking the trial, including summary of relevant studies (published and unpublished) examining benefits and harms for each intervention	4, 5,
	6b	Explanation for choice of comparators	9
Objectives	7	Specific objectives or hypotheses	5,6
Trial design	8	Description of trial design including type of trial (eg, parallel group, crossover, factorial, single group), allocation ratio, and framework (eg, superiority, equivalence, noninferiority, exploratory)	7
**Methods: Participants, interventions, and outcomes**			
Study setting	9	Description of study settings (eg, community clinic, academic hospital) and list of countries where data will be collected. Reference to where list of study sites can be obtained	7
Eligibility criteria	10	Inclusion and exclusion criteria for participants. If applicable, eligibility criteria for study centres and individuals who will perform the interventions (eg, surgeons, psychotherapists)	6,7
Interventions	11a	Interventions for each group with sufficient detail to allow replication, including how and when they will be administered	8,9, 10
	11b	Criteria for discontinuing or modifying allocated interventions for a given trial participant (eg, drug dose change in response to harms, participant request, or improving/worsening disease)	-
	11c	Strategies to improve adherence to intervention protocols, and any procedures for monitoring adherence (eg, drug tablet return, laboratory tests)	-
	11d	Relevant concomitant care and interventions that are permitted or prohibited during the trial	10
Outcomes	12	Primary, secondary, and other outcomes, including the specific measurement variable (eg, systolic blood pressure), analysis metric (eg, change from baseline, final value, time to event), method of aggregation (eg, median, proportion), and time point for each outcome. Explanation of the clinical relevance of chosen efficacy and harm outcomes is strongly recommended	10-11
Participant timeline	13	Time schedule of enrolment, interventions (including any run-ins and washouts), assessments, and visits for participants. A schematic diagram is highly recommended (see Figure)	15/16

For peer review only - http://bmjopen.bmj.com/site/about/guidelines.xhtml

Sample size	14	Estimated number of participants needed to achieve study objectives and how it was determined, including clinical and statistical assumptions supporting any sample size calculations	6
Recruitment	15	Strategies for achieving adequate participant enrolment to reach target sample size	-
**Methods: Assignment of interventions (for controlled trials)**			
Allocation:			
Sequence generation	16a	Method of generating the allocation sequence (eg, computer-generated random numbers), and list of any factors for stratification. To reduce predictability of a random sequence, details of any planned restriction (eg, blocking) should be provided in a separate document that is unavailable to those who enrol participants or assign interventions	8
Allocation concealment mechanism	16b	Mechanism of implementing the allocation sequence (eg, central telephone; sequentially numbered, opaque, sealed envelopes), describing any steps to conceal the sequence until interventions are assigned	8
Implementation	16c	Who will generate the allocation sequence, who will enrol participants, and who will assign participants to interventions	8
Blinding (masking)	17a	Who will be blinded after assignment to interventions (eg, trial participants, care providers, outcome assessors, data analysts), and how	11
	17b	If blinded, circumstances under which unblinding is permissible, and procedure for revealing a participant's allocated intervention during the trial	N/A
**Methods: Data collection, management, and analysis**			
Data collection methods	18a	Plans for assessment and collection of outcome, baseline, and other trial data, including any related processes to promote data quality (eg, duplicate measurements, training of assessors) and a description of study instruments (eg, questionnaires, laboratory tests) along with their reliability and validity, if known. Reference to where data collection forms can be found, if not in the protocol	10-11
	18b	Plans to promote participant retention and complete follow-up, including list of any outcome data to be collected for participants who discontinue or deviate from intervention protocols	

Data management	19	Plans for data entry, coding, security, and storage, including any related processes to promote data quality (eg, double data entry; range checks for data values). Reference to where details of data management procedures can be found, if not in the protocol	13
Statistical methods	20a	Statistical methods for analysing primary and secondary outcomes. Reference to where other details of the statistical analysis plan can be found, if not in the protocol	12/13
	20b	Methods for any additional analyses (eg, subgroup and adjusted analyses)	N/A
	20c	Definition of analysis population relating to protocol non-adherence (eg, as randomised analysis), and any statistical methods to handle missing data (eg, multiple imputation)	N/A
**Methods: Monitoring**			
Data monitoring	21a	Composition of data monitoring committee (DMC); summary of its role and reporting structure; statement of whether it is independent from the sponsor and competing interests; and reference to where further details about its charter can be found, if not in the protocol. Alternatively, an explanation of why a DMC is not needed	
	21b	Description of any interim analyses and stopping guidelines, including who will have access to these interim results and make the final decision to terminate the trial	N/A
Harms	22	Plans for collecting, assessing, reporting, and managing solicited and spontaneously reported adverse events and other unintended effects of trial interventions or trial conduct	9/10
Auditing	23	Frequency and procedures for auditing trial conduct, if any, and whether the process will be independent from investigators and the sponsor	N/A
**Ethics and dissemination**			
Research ethics approval	24	Plans for seeking research ethics committee/institutional review board (REC/IRB) approval	13
Protocol amendments	25	Plans for communicating important protocol modifications (eg, changes to eligibility criteria, outcomes, analyses) to relevant parties (eg, investigators, REC/IRBs, trial participants, trial registries, journals, regulators)	

Consent or assent	26a	Who will obtain informed consent or assent from potential trial participants or authorised surrogates, and how (see Item 32)	7
	26b	Additional consent provisions for collection and use of participant data and biological specimens in ancillary studies, if applicable	N/A
Confidentiality	27	How personal information about potential and enrolled participants will be collected, shared, and maintained in order to protect confidentiality before, during, and after the trial	13
Declaration of interests	28	Financial and other competing interests for principal investigators for the overall trial and each study site	14
Access to data	29	Statement of who will have access to the final trial dataset, and disclosure of contractual agreements that limit such access for investigators	-
Ancillary and post-trial care	30	Provisions, if any, for ancillary and post-trial care, and for compensation to those who suffer harm from trial participation	N/A
Dissemination policy	31a	Plans for investigators and sponsor to communicate trial results to participants, healthcare professionals, the public, and other relevant groups (eg, via publication, reporting in results databases, or other data sharing arrangements), including any publication restrictions	13
	31b	Authorship eligibility guidelines and any intended use of professional writers	N/A
	31c	Plans, if any, for granting public access to the full protocol, participant-level dataset, and statistical code	N/A
**Appendices**			
Informed consent materials	32	Model consent form and other related documentation given to participants and authorised surrogates	included
Biological specimens	33	Plans for collection, laboratory evaluation, and storage of biological specimens for genetic or molecular analysis in the current trial and for future use in ancillary studies, if applicable	N/A

*It is strongly recommended that this checklist be read in conjunction with the SPIRIT 2013 Explanation & Elaboration for important clarification on the items. Amendments to the protocol should be tracked and dated. The SPIRIT checklist is copyrighted by the SPIRIT Group under the Creative Commons "Attribution-NonCommercial-NoDerivs 3.0 Unported" license.

For peer review only - http://bmjopen.bmj.com/site/about/guidelines.xhtml

For peer review only

One to one befriending for people with intellectual disability and symptoms of depression: A protocol for a pilot randomised controlled trial.

Journal:	*BMJ Open*
Manuscript ID	bmjopen-2019-033989.R3
Article Type:	Protocol
Date Submitted by the Author:	29-Feb-2020
Complete List of Authors:	Ali , Afia; University College London, Division of Psychiatry<br>McKenzie, Emma; North East London NHS Foundation Trust Goodmayes Hospital<br>HASSIOTIS, ANGELA; ROYAL FREEand UNIVERSITY COLLEGE MEDICAL SCHOOL, PSYCHIATRY and BEHAVIOURAL SCIENCES<br>Priebe, Stefan; Barts and the London School of Medicine and Dentistry, University of London, Unit of Social and Community Psychiatry<br>Lloyd-Evans, Brynmor ; UCL, Division of Psychiatry<br>Omar, Rumana; University College London<br>Jones, Rebecca; UCL<br>Panca, Monica; University College London, Primary Care and Population Health<br>Fernandez, Vincent; Outward<br>Finning, Sally; Outward<br>Moore, Shirley; The Befriending Scheme<br>O'Connor, Danielle; The befriending Scheme<br>Roe, Christine; The Befriending Scheme<br>King, Michael; University College Medical School, Division of Psychiatry
**Primary Subject Heading**:	Mental health
Secondary Subject Heading:	Public health
Keywords:	Depression & mood disorders < PSYCHIATRY, QUALITATIVE RESEARCH, Delirium & cognitive disorders < PSYCHIATRY

SCHOLARONE™
Manuscripts

I, the Submitting Author has the right to grant and does grant on behalf of all authors of the Work (as defined in the below author licence), an exclusive licence and/or a non-exclusive licence for contributions from authors who are: i) UK Crown employees; ii) where BMJ has agreed a CC-BY licence shall apply, and/or iii) in accordance with the terms applicable for US Federal Government officers or employees acting as part of their official duties; on a worldwide, perpetual, irrevocable, royalty-free basis to BMJ Publishing Group Ltd ("BMJ") its licensees and where the relevant Journal is co-owned by BMJ to the co-owners of the Journal, to publish the Work in this journal and any other BMJ products and to exploit all rights, as set out in our licence.

The Submitting Author accepts and understands that any supply made under these terms is made by BMJ to the Submitting Author unless you are acting as an employee on behalf of your employer or a postgraduate student of an affiliated institution which is paying any applicable article publishing charge ("APC") for Open Access articles. Where the Submitting Author wishes to make the Work available on an Open Access basis (and intends to pay the relevant APC), the terms of reuse of such Open Access shall be governed by a Creative Commons licence – details of these licences and which Creative Commons licence will apply to this Work are set out in our licence referred to above.

Other than as permitted in any relevant BMJ Author's Self Archiving Policies, I confirm this Work has not been accepted for publication elsewhere, is not being considered for publication elsewhere and does not duplicate material already published. I confirm all authors consent to publication of this Work and authorise the granting of this licence.

For peer review only

One to one befriending for people with intellectual disability and symptoms of depression: A protocol for a pilot randomised controlled trial.

Afia Ali, *[1] Emma McKenzie [2], Angela Hassiotis [1], Stefan Priebe [3], Brynmor Lloyd-Evans [1], Rumana Omar [4], Rebecca Jones [1,5], Monica Panca [5], Vincent Fernandez [6], Sally Finning[6], Shirley Moore [7], Danielle O'Connor [7] Christine Roe [7], Michael King [1]

[1]Division of Psychiatry,

University College of London, UK

afia.ali@ucl.ac.uk

michael.king@ucl.ac.uk

b.lloyd-evans@ucl.ac.uk

a.hassiotis@ucl.ac.uk

reb.jones@ucl.ac.uk

[2] Research and Development Office,

Goodmayes Hospital, North East London NHS Foundation Trust, UK

Emma.mckenzie@nelft.nhs.uk

[3]Unit for Social and Community Psychiatry, (WHO Collaborating Centre for Mental Health Services Development),

Queen Mary University of London, UK

s.priebe@qmul.ac.uk.

[4] Department of Statistical Science

University College London, UK

r.omar@ucl.ac.uk

[5] PRIMENT Clinical Trials Unit,

University College London, UK

reb.jones@ucl.ac.uk

For peer review only - http://bmjopen.bmj.com/site/about/guidelines.xhtml

m.panca@ucl.ac.uk

[6]Outward, Newlon House

4 Daneland Walk, Hale Village, London,

N17 9FE

VFernandez@outward.org.uk

sfinning@outward.org.uk

[7] The Befriending Scheme,

1, The Croft, Sudbury,

Co10 1HN

danielle.oconnor@thebefriendingscheme.org.uk

christine.roe@thebefriendingscheme.org.uk

shirley.moore@thebefriendingscheme.org.uk

* Corresponding author

Afia Ali

Division of Psychiatry,

University College of London,

6th Floor, Maple House,

149 Tottenham court Road,

W1T 7NF

Email: afia.ali@ucl.ac.uk

Telephone: 0207 679 9334

Word count: 4919

For peer review only

For peer review only - http://bmjopen.bmj.com/site/about/guidelines.xhtml

Abstract

Introduction

People with intellectual disability (ID) are more likely to experience loneliness and have smaller social networks, which increases vulnerability to depression. Befriending may reduce depressive symptoms in other populations but randomised controlled trials (RCTs) have not been carried out in this population. This pilot study aims to assess the acceptability and feasibility of carrying out a full RCT of one to one befriending by volunteers for people with ID, compared to an active control group.

Methods and Analysis

The trial aims to recruit 40 participants with ID. Participants in the intervention arm will receive weekly visits from a volunteer over six months. Community befriending schemes will recruit, train, supervise volunteers and match them to individuals with ID. Both groups will receive a booklet about local activities and have access to usual care. Health and social outcomes will be measured at the end of the intervention and 6 months follow up. The following outcomes will be assessed: 1. recruitment and retention of individuals with ID and volunteers in the trial; 2. adverse events related to the intervention; 3. the acceptability of the intervention; 4. whether the intervention is delivered as intended; 5. changes in health and social outcomes and 6. the feasibility of carrying out a cost effectiveness analysis in a full trial. Qualitative data from participants, volunteers, staff and carers will identify barriers and facilitators of a future full trial.

Ethics and Dissemination

The study has been approved by the London- City and East Research Ethics Committee (reference 18/LO/2188). The findings will be presented at conferences and published in a peer reviewed journal and the National Institute of Health Research journals library. A public engagement seminar will be held at the end of the study aimed at key stakeholders.

Trial registration Number: ISRCTN63779614

Strengths and Limitations of the study

- This is the first pilot randomised controlled trial of one to one befriending in people with ID.
- This pilot study will examine the feasibility and acceptability of carrying out a future randomised controlled trial of befriending compared to an active control group.
- Community befriending services will be responsible for all aspects of the delivery of intervention
- Availability of resources within befriending services may influence the delivery of the intervention
- The findings will inform whether a full trial is feasible and what modifications would need to be made to overcome potential barriers.

For peer review only - http://bmjopen.bmj.com/site/about/guidelines.xhtml

Introduction

Intellectual disability (ID) is a life-long condition characterised by impaired cognitive and adaptive functioning, arising before the age of 18 (1). The UK prevalence of ID is 1-2% (2). People with ID have the same or higher prevalence of depression than the general population, but are more likely to experience chronic depression (3-4). They have greater exposure to social disadvantage (5), experience social exclusion because of stigma (6), have markedly smaller social networks (7-8) and a higher prevalence of loneliness compared to the general population (9). These factors have been associated with depressive symptoms in this group (10-13).

Conceptualisation of befriending

Befriending is "a relationship between two or more individuals, initiated, supported and monitored by an agency. The relationship is non-judgmental, mutual, purposeful, and there is a commitment over time" (14). Key attributes are that it is a one to one friendship-like relationship, it is an organised intervention, and that there is a negotiation of power (15). There is a wide variation in the concept and practice of befriending (16). At one extreme, befriending is very close to a friendship, characterised as being reciprocal and is delivered by lay volunteers, and at the other end it is a professional and therapeutic relationship, focused on the befriendee attaining goals and aspirations (mentoring). Most types of befriending relationships lie midway on this spectrum. Schemes are usually run by voluntary organisations in the community and offer training, supervision and on-going support to volunteers (16).

Befriending aims to help people who are lonely, isolated and have limited opportunities for social participation by increasing social and emotional support and by enhancing social networks and community participation. The causal mechanisms of befriending are unclear but social support is thought to be important. Social support may act as a buffer to stress and it may mediate genetic and environmental vulnerabilities to depression through its effects on neurobiological factors and other psychosocial factors (e.g. coping strategies) (17)The Befriender may enhance social support and link the befriendee into social activities, which may be sustainable outside of, and beyond the end of the befriending relationship, which may result in longer term benefits. Befriending may also improve health outcomes thorough its effects on social networks (18).

There are also potential benefits for befrienders. Motivation for befriending often includes a desire for "giving" something back to the community (e.g. helping others) and "getting" something in return (e.g. acquiring new skills or meeting new people) (19, 20)). Positive benefits reported by befrienders include increased self-esteem and confidence, feeling that they have gained a genuine friend themselves and improved attitudes towards people with mental illness (19). Volunteering in general has beneficial effects on depression, psychological wellbeing and life satisfaction, and is associated with lower risk of mortality, although the causal mechanisms for these associations are unclear (21).

However, the benefits of befriending may be short-lived and people with ID have reported feeling distressed following the termination of their befriending relationship (22). Other risks include the emotional turbulence that is associated with a natural friendship, or harmful effects if the befriender is not adequately trained or supervised. There may also be undue burden placed on the befriender to take on excessive responsibility (16).

For peer review only - http://bmjopen.bmj.com/site/about/guidelines.xhtml

Effectiveness of befriending

One meta-analysis found that befriending in people with mental or physical health problems, (delivered by social and health care professionals as well as lay volunteers) had a statistically significant but modest effect on reducing symptoms of depression when compared to no treatment or treatment as usual in both the short and long term (23). Another systematic review examined a range of health and social outcomes in studies where befriending was delivered by volunteers only (24). Befriending was associated with better patient reported outcomes when all primary outcomes were combined but the effect size was small (24). However, in contrast to the previous review, there was limited evidence for the effectiveness of befriending on individual outcomes such as depression, loneliness or quality of life when the studies were combined (24). A recently published randomised controlled trial (RCT) of befriending by volunteers in people with psychosis provides further evidence for the potential beneficial effects of befriending (25). Participants in the intervention arm had significantly more social contacts at the end of the 12 month intervention and at six months follow up, suggesting that befriending may help to reduce social isolation in this is group.

The effectiveness of befriending in people with ID has not been evaluated in a randomised trial. An unpublished single arm feasibility study of one to one befriending by volunteers, conducted by a voluntary organisation, (26) recruited 24 volunteers, of which 15 were matched with an individual with ID. Positive change was reported in 60% of individuals with ID; 53% reported a decrease in isolation and 40% reported an increase in confidence. One Australian study examined the feasibility of using “active mentoring”, whereby members of existing community groups were trained to act as mentors to older adults with ID, in order to provide social support and to encourage participation in community groups (27). The intervention comprised 29 individuals receiving the intervention and a matched comparison group. The participants in the intervention reported better social satisfaction compared to the comparison group but there were no significant changes in the other outcomes.

Given the dearth of studies examining befriending in people with ID there is a clear rationale for carrying out a pilot study prior to a full randomised controlled trial.

Aims and Objectives

The main aim of the study is to determine the feasibility and acceptability of a full scale randomised controlled trial of one to one befriending by volunteers for people with ID in addition to usual care, compared to an active control arm.

The objectives are to:

- examine the recruitment and retention of individuals with ID and volunteers in the trial and the number of successfully matched pairs within the six month study recruitment period
- Record any negative consequences/adverse effects of befriending
- Measure the extent to which the intervention is delivered as intended by volunteers and the befriending schemes
- Examine the acceptability of the intervention and study procedures by exploring the views of individuals, volunteers, carers and befriending services.

For peer review only - http://bmjopen.bmj.com/site/about/guidelines.xhtml

For peer review only

- Examine changes in health and social outcomes by carrying out exploratory analyses of the impact of befriending on depressive symptoms measured by the Glasgow Depression Scale (GDS (28)) at 12 months and other outcomes (psychological distress, self-esteem, loneliness, quality of life, and social participation) at 6 and 12 months post randomisation.
- Carry out exploratory analyses of the impact of befriending on volunteers' wellbeing, self-esteem, loneliness and attitudes towards people with intellectual disability at 6 and 12 months.
- Estimate the sample size required and determine the final trial design for a full scale randomised controlled trial.
- Assess the feasibility of collecting data that would inform a future analysis of cost effectiveness.

Methods

Design

This is a two arm, parallel group, researcher blind pilot RCT with 1:1 allocation. Fifty participants with ID who are eligible for the study will be randomly allocated to either the intervention arm (one to one befriending by a volunteer) or an active control arm. Both groups will have access to usual care and a booklet of local resources. The duration of the intervention will be 6 months. Outcome assessments will be carried out at baseline, post intervention and at six months follow-up. A process evaluation, based on mixed methods, will be carried out to examine the delivery and adherence to intervention, and stakeholder views on the acceptability of the intervention and barriers and facilitators that may affect the implementation of a full scale trial.

Sample Size

We do not have any estimates of the number of people with ID who are eligible and are likely to consent to taking part in the trial. If we approach 50 participants who are eligible to take part, this will allow us to estimate an expected recruitment rate of 80% (40 people), with a 95% confidence interval (CI) of 68.9% to 91.1%. A sample size of 40 recruited people with ID would allow us to estimate a 30% drop out rate in the trial with a 95% CI of 25.7% to 54.3%. The recruitment period is 6 months. There are two participating befriending services and therefore we will need to recruit 3.3 participants with ID per month at each site. Twenty volunteers will need to be recruited and matched to each participant in the intervention arm.

Participants

Inclusion Criteria

We will include: individuals with ID aged 18 or over, who have mild or moderate intellectual disability (IQ of 35-60) assessed using the Wechsler Abbreviated Scale of Intelligence (WASI-II; (29)); should not be attending college/education or a day service for three or more days a week; will have a score of 5 or more on the Glasgow Depression Scale for People with Learning Disability (GDS-LD (28)), indicating the presence of depressive symptoms but

For peer review only - http://bmjopen.bmj.com/site/about/guidelines.xhtml

do not need to have a clinical diagnosis of depression. Participants will need to be able to speak English and provide informed consent.

Volunteers aged 18 or over, who can agree to being available once a week for at least one hour over a period of six months. Volunteers do not need to have any prior experience of supporting people with ID.

Exclusion Criteria

Individuals with ID will be excluded if they have limited communication and comprehension and therefore would not be able to complete the questionnaires.

Volunteers will be excluded if they have a criminal record (any documented offence due to the vulnerability of this population) recorded on their Disclosure and Barring Service (DBS) check or are unable to provide two references or have unsuitable references.

Recruitment

Befriending services

Two befriending services (“Outward”, based in Hackney, North London and “the befriending scheme” in Suffolk) will be taking part in the study and will be responsible for overseeing the befriending intervention. Both services have experience supporting befriending relationships with people with ID including matching individuals with volunteers and monitoring relationships.

Participants with ID

Participants with ID will be recruited from existing and new referrals to the befriending services or recruited from the caseload of clinicians working in community learning disability services in North London and Suffolk. Volunteer coordinators and clinicians will screen referrals and caseloads for participants who may be eligible for the study and will make the initial approach. If the individual is interested in the study, and consents to his/her details being passed on to the research team, a referral form will be completed and emailed to the research team. The research assistant will then contact the individual and carry out an eligibility assessment. If the individual is eligible, they will need to provide written consent before completing the baseline assessment.

Volunteers

The befriending services will advertise and recruit volunteers through newspaper advertisements, befriending and job websites, social media and recruitment events at colleges and universities. Volunteers will be recruited directly from the study website hosted by the UCL Division of Psychiatry and through the Division’s twitter account. Interested volunteers will complete an application form and will be invited to an informal interview to assess their suitability and motivation for taking part in the scheme. A DBS check will be completed to ensure that they have no criminal records and references will be obtained. People with ID are potentially a vulnerable group and therefore volunteers with any previous offence, including minor offences, will not be included in the study. Successful candidates will be invited to take

For peer review only - http://bmjopen.bmj.com/site/about/guidelines.xhtml

part in the study. They will receive an information sheet and asked to sign a consent form, followed by completion of the baseline assessment.

Randomisation

Randomisation will be carried out after the baseline assessments have taken place. Participants will be randomised into the study by an unblinded member of the research team who will enter the patients' details into a web based randomisation system, which is hosted on a secure server by Sealed Envelope. This system will randomly allocate the participant to either the intervention or control arm. The unblinded researcher will notify the befriending service and participants with ID of their allocation.

Randomisation will be blocked using randomly varying block sizes, stratified by centre. The allocation schedule will be concealed through the use of this central web-based randomisation service. The randomisation protocol will be created by the trial statistician and the set up of the service will be overseen by the Priment Clinical Trials Unit.

Intervention group

Participants with ID will meet with the volunteering coordinator to obtain information about their hobbies, interests and activities they would like support to participate in. Based on this information, participants with ID will be matched to a volunteer that can accommodate the person's interests.

i. The befriending intervention

The befriending intervention has been adapted from the existing models used by the two participating befriending services and from other studies of befriending (26, 30). The purpose of the befriending relationship will be to provide friendship and emotional support, and to assist the individual to access activities in the community that they may be unable to do alone. As participants become more confident, they will be encouraged to access activities in the community on their own, in order to promote sustained social activities beyond the befriending relationship.

The volunteer (befriender) and person with ID will meet once a week for at least one hour, for six months, although some breaks are anticipated due to holidays or illness. They will receive a booklet detailing local activities and amenities, which they can use to plan activities. The emphasis will be on assisting the individual to make choices about the activities that they wish to pursue. The volunteer is not expected to carry out personal care, administer medication or accompany the individual to medical appointments. Contacts by phone/social media can take place in addition to face to face contacts. The pair can spend some sessions in the person's home but this should not exceed 50% of the total number of sessions. Sessions may take place during evenings/weekends depending on the pair's availability. They will keep a record of their activities in a structured log that will be provided (whether they attended each session, reasons for cancellation, what they did in each session and duration of activity), and record of other types of contact. Volunteers will be reimbursed travel expenses but other expenses will need to be agreed with the befriending service. Participants with ID will not be reimbursed travel expenses or the costs of activities.

ii. Introduction and monitoring of the befriending relationship

For peer review only - http://bmjopen.bmj.com/site/about/guidelines.xhtml

The volunteering coordinator will arrange a face-to-face meeting where the pair will be introduced to each other. If they agree to continue, the pair will arrange to meet on their own; if they decide that the pairing is unsuitable, they will be re-matched. If the volunteer or individual with ID drops out of the relationship once it has become established, attempts will be made to re-match them. Volunteers may be matched to more than one participant with ID.

The volunteering coordinator will arrange a face-to-face meeting with the pair after six weeks and will maintain contact with each person by telephone/ face to face contact every four weeks thereafter to monitor the progress of the relationship. A further meeting will be held with the pair at the end of the six months to obtain general feedback about the befriending intervention, to discuss ending the relationship and to support the individual with ID with coming to terms with the ending. The pair may continue their relationship if they wish after the six month period but arrangements for monitoring the relationship will vary depending on the befriending service. Information will be obtained on any relationships continuing beyond six months and the monitoring that has been provided.

iii. Training and supervision of volunteers

The volunteers will attend training delivered face-to-face and as e-learning. The training will cover: the benefits of befriending and issues related to confidentiality and lone working; advice on how to plan meetings effectively; health and safety; safeguarding ; learning disability awareness, and professional boundaries, which covers dealing with sensitive issues, ending relationships and expectations of the role of the volunteer. Volunteers will also receive slides and a manual developed by the research team, with information about the study and additional information about communication, challenging behaviour and mental health problems.

Volunteers will have access to group or individual supervision delivered face to face or over the phone, once a month, by the volunteer coordinator, which will address issues that may have arisen from the relationship, for example, concerns about mental health or behaviour.

iv. Training and support for befriending services

Participating befriending services will complete Good Clinical Practice (GCP) Training and undergo training on the study research processes and procedures as part of the site initiation visit. Volunteer coordinators will receive support from the research team through regular email and telephone contact and they will attend Trial Management Group (TMG) meetings.

Control Group

Participants in the control arm will receive the activities booklet. They will meet with a member of the research team who will discuss the booklet with them (and their carer, if present) and encourage them to engage with activities. This is to control for the effects of participants in the treatment arm receiving more information about local activities.

Both the control and intervention arms will also have access to “usual care”. This will include access to multidisciplinary input from community intellectual disability services. Participants

For peer review only - http://bmjopen.bmj.com/site/about/guidelines.xhtml

For peer review only

can continue to take their usual medication and can access other community and hospital health services and day services. Information about usual care will be collected as part of the baseline assessment.

Outcomes

i. Recruitment and retention of participants:

We will examine the proportion of participants with ID recruited from among those eligible; and the proportion of volunteers recruited from among people expressing an interest over a six months period; the proportion of participants with ID who are successfully matched with a volunteer; the proportion of participants with ID and volunteers who drop out of the intervention arm and the proportion of participants and volunteers who complete subsequent follow up assessments.

ii. Adverse events

Adverse events will be collected at each follow up assessment using open-ended questions and will also be reported directly to the Chief Investigator by the befriending services, including concerns about safeguarding. Volunteers will follow a protocol if they have concerns about the participant's mental health, which will involve informing the volunteer coordinator, who will in turn notify the CI and the referring clinician or a health professional involved in the person's care. If a serious incident (e.g. self -harm, challenging behaviour) occurs out of hours, the carer will be notified and emergency services will be contacted if appropriate. All adverse events will be recorded in the medical records and CRFs. Serious adverse events (e.g. safeguarding concerns, hospitalisation) will be recorded in the Serious Adverse Effects (SAE) log.

iii. Acceptability of the intervention

This will be informed by data on retention/ drop out of volunteers and participants, the extent of engagement with the intervention by participants and volunteers (based on number of sessions attended) and qualitative data obtained from volunteers, participants with ID, carers of people with ID and staff from the befriending service.

iv. Adherence to the intervention

Data will be collected on volunteer training, uptake of supervision and the frequency of monitoring checks carried out by volunteer coordinators from routine records at each site in order to assess fidelity to the intervention by the befriending services. Structured logs provided by volunteers will be analysed to assess fidelity to the intervention by volunteers. We will examine: a) how many sessions were attended by each volunteer, reasons for non-attendance, and how many attended at least 10 sessions during the 6-month intervention period; and b) for how many participants the minimum threshold of at least 50% of meetings being outside the participant's home was achieved.

v. Depression and other health and social outcome measures in participants with ID

For peer review only - http://bmjopen.bmj.com/site/about/guidelines.xhtml
For peer review only

All the health and social outcome measures have been validated in people with ID and will be assessed with the participant at baseline, at the end of the intervention and at six months follow up. (see table 1: schedule of assessments). Assessments will be carried out by a research assistant (RA) blind to group allocation and will take place face to face at the befriending service or the participants' homes.

- Depressive symptoms will be measure using the GDS-LD (28). Scores at 12 months will be the main health outcome of interest.
- Self-esteem will be measured using the adapted Rosenberg self-esteem scale for people with intellectual disabilities (31)
- Quality of life will be measured using the Maslow Assessment of Needs Scale-Learning Disability (MANS-LD; (32)) and five items from the adapted World Health Organisation Quality of Life questionnaire (WHO-QOL-8; (33)).
- Loneliness and social satisfaction will be measured using the Modified Worker Loneliness Questionnaire (MWLQ (34),
- Social support will be measured using the Social Support Self Report for intellectually disabled adults (SSSR) (35)
- Social participation will be measured at using the Guernsey Community Participation and Leisure Assessment (GCPLA) (36).

vi. Volunteer outcome measures

The following outcome assessments will be carried out in volunteers at baseline (prior to matching) and post intervention (six months after the baseline) and at six months follow-up (see table 2 schedule of assessments):

- Self-esteem will be measured using the 10 item Rosenberg self-esteem scale (37)
- Psychological wellbeing and quality of life will be measured using the Warwick-Edinburgh mental wellbeing scale (WEMWBS; 38).
- Loneliness will be measured using the UCLA loneliness scale (39).
- Attitudes of volunteers will be assessed using the 67 item, Attitudes Towards Intellectual Disability Questionnaire (ATTID; 40)

vii. Feasibility of carrying out a cost effectiveness analysis

The preliminary health economic analysis will inform planning of future economic analyses, sources of data required and how best to collect these data. The following measures will be recorded at baseline, post intervention (six months after randomisation) and at six months follow-up.

- Health related quality of life will be measured using the EuroQol-Youth (EQ-5D-Y) (41). We will assess the feasibility of using the EQ-5D-Y to calculate Quality adjusted life years (QALYs). QALYs will be calculated using the EQ-5D-3 L tariff, as there are no value sets for the EQ-5 D-Y (42).
- Client Services Receipt Inventory (CSRI) for people with intellectual disabilities adapted for the study (43), completed with carers where possible, will assess the feasibility of collecting participant reported service use over the previous six months.

For peer review only - http://bmjopen.bmj.com/site/about/guidelines.xhtml

Process evaluation

A process evaluation will be undertaken, based on MRC guidance (44). The aim will be to examine whether the different components of the intervention were consistently followed; the extent to which volunteers delivered the intervention as intended; to understand the perceived value, benefits and unintended consequences of the intervention so that these are fully measured in a full trial, contextual factors influencing how the intervention was delivered; and to develop an understanding of the likely mechanisms of action of the intervention. We will interview 16 participants with ID after their 12 month follow up (8 per site) and carry out two focus groups at each site with 5-8 volunteers, staff from each befriending service, and 5-8 carers. We will use purposive sampling in order to include people with a range of demographic characteristics (e.g. gender, age, ethnicity, living arrangements), and both participants and volunteers for whom the befriending relationship broke down, as well as people who completed the intervention. All respondents will be asked about what aspects of the intervention were perceived to be helpful, what aspects require modification and suggestions for improvement, views about trial procedures and the perceived barriers and facilitators to delivering a larger trial. The interviews and focus groups will be audio-taped and transcribed.

In order to understand how the befriending intervention was delivered, the location, content of the meetings and the range of activities undertaken will be described based on an analysis of structured log books. A framework will be developed to categorise different types of activity to enable different types of befriending support to be distinguished and quantified. Test procedures will be developed for collecting quantitative process data, which, in a future larger trial, could be used to explore the relationships between process variables and outcomes. Findings from the process evaluation will inform any necessary refinements to the study intervention or procedures.

Analysis

Feasibility outcomes such as the number of participants who were screened and eligible, the proportion of eligible participants with ID and interested volunteers recruited to the study, and the proportion successfully matched in befriending relationships, will be reported. The number (proportion) of drop-outs of volunteers and participants from the intervention arm and from both arms at each follow up assessment, will also be reported, including reasons why where possible. This information will be presented in a Consort diagram describing the flow of participants through the study. (http://www.consort-statement.org/).

The characteristics and outcomes of participants by trial arm will be summarised using means and standard deviations or medians and inter-quartile ranges for continuous variables and count and percentages for categorical variables. Appropriate regression models depending on the type of outcome, adjusted for baseline values and centre will be used to estimate the intervention effect on health and social outcomes where possible. The results will be presented as estimates with 95% CI. All analyses will be carried out as randomised with available data (intention to treat principle) and will be used to inform the definitive trial. The characteristics of patients with missing outcome data will be investigated.

Economic Evaluation

For peer review only - http://bmjopen.bmj.com/site/about/guidelines.xhtml

We will assess the feasibility of gathering information for a cost-effectiveness analysis for a full RCT including testing the suitability of calculating quality-adjusted life years (QALYs) using the EQ-5D-Y. We will calculate the costs of delivering the befriending intervention (training, supervision and expenses), which will be obtained from each participating befriending service. Mean resource use/ costs (standard deviation (SD)) per participant at baseline and follow-up, based on completion of the CSRI, will be presented.

Qualitative analysis

Transcripts from the focus groups and interviews will be analysed by the study team using thematic analysis supported by computer software (NVivo 9). The analytic strategy will focus on identifying barriers and facilitators to implementing the intervention successfully, its benefits to participants and mechanisms of effect. Analysis will also allow consideration of themes that arise more inductively from the data. Validity will be enhanced by a collaborative analytic strategy involving members of the research team and the advisory group who will meet together to review the coding framework and agree on the themes.

Patient and Public Involvement

Volunteers, people with ID and befriending schemes were involved in the design of the study. During the study, there will be advisory groups comprising carers, current volunteers and individuals with ID who will provide advice about the study information sheets, consent forms, topic guides for the qualitative interviews and focus groups, results of the study findings and the final study report. Members will be invited to be part of the TMG and will be involved in carrying out the qualitative interviews and focus groups as part of the process evaluation. They will receive training and support for this role. We will also invite members to participate in the public engagement seminar at the end of the study.

Ethics, governance and dissemination

Amendments to the study protocol and documents will be approved by the sponsor (UCL) and the Ethics Committee. Priment Clinical Trials Unit (CTU) will ensure that the trial procedures meet the requirements of Good Clinical Practice (GCP) and will complete dataquality assurance checks. The RA will have experience working with people with ID and will receive training on assessing capacity and carrying out assessments. Supervision of the RA will be provided by the Chief Investigator (AA). The study team, co-applicants, PPI advisors and the CTU will be part of the TMG and will meet every 10-12 weeks to discuss the progress of the study. An independent trial steering committee will provide overall supervision of the trial and will report to the funders. Data confidentiality will be maintained by assigning participants study identification numbers and data will be entered anonymously and stored in a secure web-based database.

The findings of the study will be presented at conferences and published in an open access peer reviewed journal as well as the National Institute of Health Research (NIHR) Public Health Research Journal. A public engagement seminar targeted at relevant stakeholders will take place at the end of the study to discuss the study findings and implications. A summary report will be developed for participants and volunteers taking part in the study and will be published on the study website.

Study timeline

For peer review only - http://bmjopen.bmj.com/site/about/guidelines.xhtml

Recruitment of participants began in April 2019. There will be a six month recruitment period, a further 12 months to complete the follow up assessment and three months to complete the process evaluation, analyse the results and write up the study findings.

Discussion

This is the first pilot RCT of one to one befriending, monitored by community befriending services, in people with ID. The trial will provide data on whether a full trial will be feasible, in terms of recruitment and retention of volunteers and people with ID and data on potential beneficial and adverse effects, acceptability and the extent to which the intervention is delivered as intended by volunteers and the befriending services. It will help to inform modifications that need to be made to enable a future trial to overcome barriers and challenges that may be encountered in this pilot.

There is currently limited evidence supporting the choice of a primary health outcome but the use of depressive symptoms is supported by one systematic review (24). This pilot study will help to determine whether measuring depressive symptoms is the most appropriate primary outcome for a future trial.

One of the main challenges of this study will be to ensure that the intervention and the recruitment, training and supervision of volunteers are carried out according to the trial protocol as these aspects vary between befriending services, and was an issue in a recent trial of befriending in people with psychosis (25) While both befriending services have committed to the study, there are resource implications for these services as the intervention costs are not covered by the research funding. External factors such as funding cuts to these services could influence the delivery of the intervention and the success of the trial. The process data will allow us to examine whether a future trial would need to be more pragmatic and whether the befriending intervention should permit more flexibility.

Author Contributions: AA is the chief investigator and is responsible for the overall study design and conduct. AA, SP, MK, AH, BL-E and RO have contributed to the design of the study and methodology. SF, VF, DO, CR, SM are responsible for designing and delivering the intervention. EM will carry out the data collection. RO, RJ and MP have contributed to the analysis plan. All the authors have reviewed and approved the manuscript.

Funding statement: The study/ project is funded by the National Institute of Health Research (NIHR) (Public Health Research Funding Stream (project reference: 16/122/57). The views expressed are the views of the authors and are not necessarily those of the NIHR of the Department of Health and Social Care.

Competing Interests statement: There are no conflicting interests to declare.

For peer review only - http://bmjopen.bmj.com/site/about/guidelines.xhtml

Table 1: Schedule of assessments in participants with ID

Assessment/Outcome	Screening and baseline assessment	Post intervention assessment	6 months follow up
Eligibility confirmation:<br>WASI-II<br>GDS-LD	x		
Informed consent	x		
Socio-demographic questionnaire	x		
Adapted Rosenberg self-esteem scale	x	x	x
MANS-LD	x	x	x
WHO-QOL-8	x	x	x
MWLQ	x	x	x
SSSR	x	x	x
GCPLA	x	x	x
EQ-5D-Y	x	x	x
CSRI	x	x	x
Adverse effects review	x	x	x
Concomitant medication	x	x	x
Semi-structured interview (optional)			x

WASI-II (Wechsler Abbreviated Scale of Intelligence, 2nd edition); GDS-LD (Glasgow Depression Scale for People with Learning Disability); MANS-LD (Maslow Assessment of Needs Scale learning Disability); WHO-QOL-8 (World Health Organisation Quality of Life questionnaire); MWLQ (Modified Worker Loneliness Questionnaire; SSSR (Social Support Self Report for intellectually disabled adults); GCPLA (Guernsey Community Participation and Leisure Assessment); EQ-5D-Y (EuroQol-Youth); CSRI (Client Services Receipt Interview).

For peer review only

Table 2: Schedule of assessments with volunteers

**Assessment/ outcome**	**Screening and baseline**	**Post intervention assessment**	**6 months follow up**
Eligibility check:<br>Informal interview;<br>DBS check<br>references	x		
Informed consent	x		
Rosenberg self-esteem scale	x	x	x
WEMWBS	x	x	x
UCLA Loneliness Scale	x	x	x
ATTID	x	x	x
Focus group (optional)			x

WEMWBS Warwick- Edinburgh mental wellbeing scale; ATTID (Attitudes Towards Intellectual Disability Questionnaire)

For peer review only

References

1. *American Psychiatric Association. Diagnostic and Statistical Manual of Mental Disorders (5th ed.).* Arlington, VA: American psychiatric Publishing; 2013.

2. Emerson E, Hatton C, Robertson J, Roberts H, Baines S, Glover Gyles. *People with learning disabilities in England 2010: services and supports.* Improving Health and Lives Learning Disability Observatory, 2010 (accessed from. http://www.improvinghealthandlives.org.uk/uploads/doc/vid_9244_IHAL2011-02PWLD2010.pdf

3. Cooper S-A, Smiley E, Morrison J, Williamson A, Allan L. An epidemiological investigation of affective disorders with a population-based cohort of 1,023 adults with intellectual disabilities. *Psychological Medicine*, 2007;37:873–82.

4. Richards M, Maughan B, Hardy R, et al. Long-term affective disorder in people with mild learning disability. *British Journal of Psychiatry*, 2001;179:523-7.

5. Emerson E and Einfeld SL. *Challenging Behaviour*. Cambridge: Cambridge University Press; 2011

6. Ali A, Hassiots A, Strydom A, King M. Self stigma in people with intellectual disabilities and courtesy stigma in family carers: A systematic review. *Research in Developmental Disabilities*, 2012; 33: 2122-2140

7. Verdonschot M.M.L,deWitte L.P, Reichrath E, Buntinx W.H.E, &Curfs L.N.G. Impact of environmental factors on community participation of persons with an intellectual disability: A systematic review. *Journal of Intellectual Disability Research*, 2009; 53(1), 54–64.

8. Hill RA & Dunbar RIM. Social network size in humans. *Human Nature*, 2003; 14(1):53-72.

9. Petroutsou A, Ali A, Hassiotis A. Loneliness in people with intellectual and developmental disorders (IDD) across the life span: a systematic review of prevalence and interventions. *Journal of Applied Research in Intellectual Disability* (published online Feb 2018).

10. Ali A, King M, Strydom S, Hassiotis A. Self-reported stigma and symptoms of anxiety and depression in people with intellectual disabilities: Findings from a cross sectional study in England. *Journal of Affective Disorders*, 2015, 187:224-231

11. Emerson E. Self reported exposure to disablism is associated with poor self reported health and wellbeing among adults with intellectual disabilities in England: a cross sectional survey. *Public Health*, 2010; 124, 682-689

12. Ailey S H, Miller A M, Heller T, & Smith E V. Evaluating an interpersonal model of depression among adults with Down syndrome. *Research and Theory for Nursing Practice*, 2006; *20*(3), 229-246.

For peer review only - http://bmjopen.bmj.com/site/about/guidelines.xhtml

13. Merrick, J., Merrick, E., Lunsky, Y., & Kandel, I. A review of suicidality in persons with intellectual disability. *The Israel Journal of Psychiatry and Related Sciences*, 2006; *43*(4), 258-264.

14. Dean, J. & Goodlad, R.*Supporting Community Participation: the role and impact of befriending.* Brighton: Pavilion Publishing & Joseph Rowntree Foundation, 1998.

15. Balaam MC. A concept analysis of befriending. J Adv Nurs, 2015, 71(1): 24-34.

16. Thompson R, Valenti E, Siette J, Priebe S. To befriend or to be a friend: a systematic review of the meaning and practice of "befriending" in mental health care. *J Men Health*, 2016, 25(1):71-77.

17. Ozbay F, Johnson DC, Dimoulas E, Charney D, and Southwick S. Social Support and Resilience to Stress. From Neurobiology to Clinical Practice. *Psychiatry (Edgmont),* 2007; 4(5): 35–40.

18. Berkman L, Glass T, Brissette I, Seeman T. From social integration to health: Durkheim in the new millennium. *Social Science and Medicine*, 2000; 51; 6:843-57

19. Klug G, Klug G, Toner S, Fabisch K, Priebe S. Characteristics and motivations of volunteers providing one-to-one support for people with mental illness: a survey in Austria. *Social Psychiatry and Psychiatric Epidemiology*. 2018; 53:841-847

20 Toner S, Hickling LM, Pinto da Costa M, Cassidy M, Priebe S. Characteristics, motivations and experiences of volunteer befrienders for people with mental illness: a systematic review and narrative synthesis. *BMC Psychiatry*. 2018;18:378

21. Jenkinson CE, Dickens AP, Jones K, Thompson-Coon J, Taylor RS, Rogers M, Bambra CL, Lang I, Richards SH. Is volunteering a public health intervention? A systematic review and meta-analysis of the health and survival of volunteers. *BMC Public Health*. 2013; 13:773.

22. Heslop P. Good practice in befriending services for people with learning disability. *British Journal of Learning Disabilities*, 2005; 33:27-33.

23. Mead N, Lester H, Chew-Graham C, Gask L, Bower P et al. Effects of befriending on depressive symptoms and distress: systematic review and meta-analysis. *The British Journal of Psychiatry,* 2010, 196: 96-101.

24. Siette J, Cassidy M, Priebe S. Effectiveness of befriending interventions: a systematic review and meta-analysis. *BMJ Open*, 2017, 7:e014304.

For peer review only - http://bmjopen.bmj.com/site/about/guidelines.xhtml

25. Priebe S[1], Chevalier A[2], Hamborg T[3], Golden E[4], King M[5], Pistrang N[6]. Effectiveness of a volunteer befriending programme for patients with schizophrenia: randomised controlled trial. ;British Journal of Psychiatry. 2019; 4:1-7

26. Florides T. *Running a befriending project for people with learning disabilities. Final project report.* Circle Support; 2012 (accessed from http://www.mandbf.org/wp-content/uploads/2012/05/Running-a-Befriending-Project-for-Adults-with-Learning-Disabilites-The-Final-Report.pdf)

27. Stancliffe R J., Bigby C, Balandin S, Wilson N J, Craig D. Transition to retirement and participation in mainstream community groups using active mentoring: a feasibility and outcomes evaluation with a matched comparison group. *Journal of Intellectual Disability Research, 2015; 59*(8), 703–718.

28. Cuthill FM, Espie CA, Cooper SA. Development and psychometric properties of the Glasgow Depression Scale for people with a Learning Disability. Individual and carer supplement versions. *British Journal of Psychiatry*, 2003;182:347–53.

29. Wechsler D. Wechsler Abbreviated Scale of Intelligence, Second Edition (WASI-II). San Antonio, TX: NCS Pearson; 2011.

30. Priebe S, Pavlickova H, Eldridge S, Golden E, McCrone P, Ockenden N, Pistrang N, King M. Effectiveness of one-to-one volunteer support for patients with psychosis: protocol of a randomised controlled trial. *BMJ Open*, 2016, 6:e011582.

31. Dagnan D, Sandhu S. Social comparison, self esteem and depression in people with intellectual disability. *Journal of Intellectual Disability Research*, 1999: 372-379.

32. Skirrow P, Perry E. The Maslow Assessment of Needs Scales, 2009; Mersey Care NHS Trist, Liverpool.

33. World Health Organisation Quality of Life Assessment group. Development and general psychometric properties. Social Science and Medicine, 1998; 46: 1569-1585

34. Stancliffe RJ, Keane S. Outcomes and costs of community living: a matched comparison of group homes and semi-independent living. *Journal of Intellectual & developmental Disability,* 2000; 25: 281-305.

35. Lunsky Y, Benson B A, Reliability of ratings of consumers with mental retardation and their staff on multiple measures of social support. American Journal on Mental Retardation *1997;* 102, 280–4.

36. Baker PA. Measurement of community participation and use of leisure by service users with intellectual disabilities: The Guernsey community participation and leisure assessment (GCPLA). *Journal of Applied Research in Intellectual Disabilities*, 2000; 13:169-185

For peer review only

37. Rosenberg M. *Society and the adolescent self-image*. Princeton, NJ: Princeton University Press; 1965

38. Tennant R, Hiller L, Fishwick R, Platt S, Joseph S, Weich S et al. The Warwick-Edinburgh mental well-being scale (WEMWBS): development and UK validation. *Health and Quality of Life Outcomes*, 2007, 5(1): 63.

39. Russell, D., Peplau, L.A., & Cutrona, C.E. (1980). The revised UCLA Loneliness Scale:Concurrent and discriminant validity evidence. *Journal of Personality and Social Psychology,* 39, 472-480.

40. Morin D, Crocker AG, Beaulieu-Bergeron R, Caron J. Validation of the attitudes toward intellectual disability: ATTID questionnaire. *Journal of Intellectual Disability Research,* 2013; 57(3):268-78

41. Wille N, Badia X, Bonsel G, Burstrom K, Cavrini G, Devlin N, Egmar AC, Greiner W, Gusi N, Herdman M, Jelsma J, Kind P, Scalone L, Ravens-Sieberer U. Development of the EQ-5D-Y: a child-friendly version of the EQ-5D. *Quality of Life Research*, 2010; 19:875–886. https://euroqol.org/eq-5d-instruments/eq-5d-y-about/

42. EQ-5D-Y (Youth) About. https://euroqol.org/eq-5d-instruments/eq-5d-y-about/. Accessed November 2019.

43 Beecham J, Knapp M. Costing psychiatric interventions. In Measuring Mental Health Needs 2nd ed (ed G Thornicroft): 200–24. Gaskell, 2001

44. Moore GF, Audrey S, Barker M, Bond L, Bonell C, Hardeman W, Moore L, O'Cathain A, Tinati T, Wight D, Baird J. Process Evaluation of complex interventions: Medical Research Council Guidance. *British Medical Journal*, 2015; 350:h1258

For peer review only - http://bmjopen.bmj.com/site/about/guidelines.xhtml

STANDARD PROTOCOL ITEMS: RECOMMENDATIONS FOR INTERVENTIONAL TRIALS

SPIRIT 2013 Checklist: Recommended items to address in a clinical trial protocol and related documents*

Section/item	Item No	Description	Addressed on page number
**Administrative information**			
Title	1	Descriptive title identifying the study design, population, interventions, and, if applicable, trial acronym	1
Trial registration	2a	Trial identifier and registry name. If not yet registered, name of intended registry	2
	2b	All items from the World Health Organization Trial Registration Data Set	
Protocol version	3	Date and version identifier	N/A
Funding	4	Sources and types of financial, material, and other support	14
Roles and responsibilities	5a	Names, affiliations, and roles of protocol contributors	1, 13/14
	5b	Name and contact information for the trial sponsor	13
	5c	Role of study sponsor and funders, if any, in study design; collection, management, analysis, and interpretation of data; writing of the report; and the decision to submit the report for publication, including whether they will have ultimate authority over any of these activities	14
	5d	Composition, roles, and responsibilities of the coordinating centre, steering committee, endpoint adjudication committee, data management team, and other individuals or groups overseeing the trial, if applicable (see Item 21a for data monitoring committee)	13

For peer review only

For peer review only - http://bmjopen.bmj.com/site/about/guidelines.xhtml

**Introduction**			
Background and rationale	6a	Description of research question and justification for undertaking the trial, including summary of relevant studies (published and unpublished) examining benefits and harms for each intervention	4, 5,
	6b	Explanation for choice of comparators	9
Objectives	7	Specific objectives or hypotheses	5,6
Trial design	8	Description of trial design including type of trial (eg, parallel group, crossover, factorial, single group), allocation ratio, and framework (eg, superiority, equivalence, noninferiority, exploratory)	7
**Methods: Participants, interventions, and outcomes**			
Study setting	9	Description of study settings (eg, community clinic, academic hospital) and list of countries where data will be collected. Reference to where list of study sites can be obtained	7
Eligibility criteria	10	Inclusion and exclusion criteria for participants. If applicable, eligibility criteria for study centres and individuals who will perform the interventions (eg, surgeons, psychotherapists)	6,7
Interventions	11a	Interventions for each group with sufficient detail to allow replication, including how and when they will be administered	8,9, 10
	11b	Criteria for discontinuing or modifying allocated interventions for a given trial participant (eg, drug dose change in response to harms, participant request, or improving/worsening disease)	-
	11c	Strategies to improve adherence to intervention protocols, and any procedures for monitoring adherence (eg, drug tablet return, laboratory tests)	-
	11d	Relevant concomitant care and interventions that are permitted or prohibited during the trial	10
Outcomes	12	Primary, secondary, and other outcomes, including the specific measurement variable (eg, systolic blood pressure), analysis metric (eg, change from baseline, final value, time to event), method of aggregation (eg, median, proportion), and time point for each outcome. Explanation of the clinical relevance of chosen efficacy and harm outcomes is strongly recommended	10-11
Participant timeline	13	Time schedule of enrolment, interventions (including any run-ins and washouts), assessments, and visits for participants. A schematic diagram is highly recommended (see Figure)	15/16

For peer review only - http://bmjopen.bmj.com/site/about/guidelines.xhtml

Sample size	14	Estimated number of participants needed to achieve study objectives and how it was determined, including clinical and statistical assumptions supporting any sample size calculations	6
Recruitment	15	Strategies for achieving adequate participant enrolment to reach target sample size	-
**Methods: Assignment of interventions (for controlled trials)**			
Allocation:			
Sequence generation	16a	Method of generating the allocation sequence (eg, computer-generated random numbers), and list of any factors for stratification. To reduce predictability of a random sequence, details of any planned restriction (eg, blocking) should be provided in a separate document that is unavailable to those who enrol participants or assign interventions	8
Allocation concealment mechanism	16b	Mechanism of implementing the allocation sequence (eg, central telephone; sequentially numbered, opaque, sealed envelopes), describing any steps to conceal the sequence until interventions are assigned	8
Implementation	16c	Who will generate the allocation sequence, who will enrol participants, and who will assign participants to interventions	8
Blinding (masking)	17a	Who will be blinded after assignment to interventions (eg, trial participants, care providers, outcome assessors, data analysts), and how	11
	17b	If blinded, circumstances under which unblinding is permissible, and procedure for revealing a participant's allocated intervention during the trial	N/A
**Methods: Data collection, management, and analysis**			
Data collection methods	18a	Plans for assessment and collection of outcome, baseline, and other trial data, including any related processes to promote data quality (eg, duplicate measurements, training of assessors) and a description of study instruments (eg, questionnaires, laboratory tests) along with their reliability and validity, if known. Reference to where data collection forms can be found, if not in the protocol	10-11
	18b	Plans to promote participant retention and complete follow-up, including list of any outcome data to be collected for participants who discontinue or deviate from intervention protocols	

Data management	19	Plans for data entry, coding, security, and storage, including any related processes to promote data quality (eg, double data entry; range checks for data values). Reference to where details of data management procedures can be found, if not in the protocol	13
Statistical methods	20a	Statistical methods for analysing primary and secondary outcomes. Reference to where other details of the statistical analysis plan can be found, if not in the protocol	12/13
	20b	Methods for any additional analyses (eg, subgroup and adjusted analyses)	N/A
	20c	Definition of analysis population relating to protocol non-adherence (eg, as randomised analysis), and any statistical methods to handle missing data (eg, multiple imputation)	N/A
**Methods: Monitoring**			
Data monitoring	21a	Composition of data monitoring committee (DMC); summary of its role and reporting structure; statement of whether it is independent from the sponsor and competing interests; and reference to where further details about its charter can be found, if not in the protocol. Alternatively, an explanation of why a DMC is not needed	
	21b	Description of any interim analyses and stopping guidelines, including who will have access to these interim results and make the final decision to terminate the trial	N/A
Harms	22	Plans for collecting, assessing, reporting, and managing solicited and spontaneously reported adverse events and other unintended effects of trial interventions or trial conduct	9/10
Auditing	23	Frequency and procedures for auditing trial conduct, if any, and whether the process will be independent from investigators and the sponsor	N/A
**Ethics and dissemination**			
Research ethics approval	24	Plans for seeking research ethics committee/institutional review board (REC/IRB) approval	13
Protocol amendments	25	Plans for communicating important protocol modifications (eg, changes to eligibility criteria, outcomes, analyses) to relevant parties (eg, investigators, REC/IRBs, trial participants, trial registries, journals, regulators)	

Consent or assent	26a	Who will obtain informed consent or assent from potential trial participants or authorised surrogates, and how (see Item 32)	7
	26b	Additional consent provisions for collection and use of participant data and biological specimens in ancillary studies, if applicable	N/A
Confidentiality	27	How personal information about potential and enrolled participants will be collected, shared, and maintained in order to protect confidentiality before, during, and after the trial	13
Declaration of interests	28	Financial and other competing interests for principal investigators for the overall trial and each study site	14
Access to data	29	Statement of who will have access to the final trial dataset, and disclosure of contractual agreements that limit such access for investigators	-
Ancillary and post-trial care	30	Provisions, if any, for ancillary and post-trial care, and for compensation to those who suffer harm from trial participation	N/A
Dissemination policy	31a	Plans for investigators and sponsor to communicate trial results to participants, healthcare professionals, the public, and other relevant groups (eg, via publication, reporting in results databases, or other data sharing arrangements), including any publication restrictions	13
	31b	Authorship eligibility guidelines and any intended use of professional writers	N/A
	31c	Plans, if any, for granting public access to the full protocol, participant-level dataset, and statistical code	N/A
**Appendices**			
Informed consent materials	32	Model consent form and other related documentation given to participants and authorised surrogates	included
Biological specimens	33	Plans for collection, laboratory evaluation, and storage of biological specimens for genetic or molecular analysis in the current trial and for future use in ancillary studies, if applicable	N/A

*It is strongly recommended that this checklist be read in conjunction with the SPIRIT 2013 Explanation & Elaboration for important clarification on the items. Amendments to the protocol should be tracked and dated. The SPIRIT checklist is copyrighted by the SPIRIT Group under the Creative Commons "Attribution-NonCommercial-NoDerivs 3.0 Unported" license.

For peer review only

For peer review only - http://bmjopen.bmj.com/site/about/guidelines.xhtml
